# Pentapeptides for the treatment of small cell lung cancer: Optimisation by N^ind^-alkyl modification of the tryptophan side chain

**DOI:** 10.1016/j.ejmech.2017.05.053

**Published:** 2017-09-08

**Authors:** Osama Haitham Abusara, Sally Freeman, Harmesh Singh Aojula

**Affiliations:** Division of Pharmacy and Optometry, School of Health Sciences, Faculty of Biology, Medicine and Health, University of Manchester, Manchester M13 9PT, UK

**Keywords:** Lung cancer, Cytotoxicity, *N*-alkylated indoles, Pentapeptides

## Abstract

The pentapeptide, *tert*-Prenyl^4th^-NH_2_ (DMePhe-DTrp-Phe-DTrp(*N*-*tert*-prenyl)-Leu-NH_2_), has recently been reported by our group to exhibit properties of substance P (SP) antagonist G against small cell lung cancer (SCLC). In this study, we undertook a systematic structure activity investigation to optimise this lead compound to improve its *in vitro* anti-tumour activity and biocompatibility. A series of d-tryptophan (D-Trp) derivatives were synthesised, with a range of aliphatic *N*-alkyl chains (methyl to pentyl) on the indole nitrogen (N^ind^). These were incorporated into the pentapeptide sequence by substitution of the N^ind^-*tert*-prenylated D-Trp 4th residue with the N^ind^-alkylated D-Trp derivatives. These pentapeptides were significantly more potent than *tert*-Prenyl^4th^-NH_2_, with the N^ind^-butyl modification generating the most cytotoxic peptides. Compared to *tert*-Prenyl^4th^-NH_2_, a single butyl modification on the 4th D-Trp residue (Butyl^4th^-NH_2_) showed a ∼3 fold enhancement in cytotoxicity in either the chemo-naive H69 or the DMS79 (originating from a patient treated with chemotherapeutics and radiation therapy) SCLC cell lines. In addition, the di-butylated sequence on the 2nd and 4th D-Trp residues (Butyl^2nd,4th^-NH_2_) gave ∼4.5 times higher cytotoxicity against the H69 cell line and a ∼2 fold increase against the DMS79 cell line, compared to *tert*-Prenyl^4th^-NH_2_. The favoured position for butyl modification was the 4th D-Trp residue, as the Butyl^2nd^-NH_2_ peptide gave lower cytotoxicity on both cell lines. Butylated peptide sequences, when exposed to neat mouse plasma for 24 h at 37 °C, were found to resist degradation with >80% remaining intact compared to ∼58% for *tert*-Prenyl^4th^-NH_2_. The degradation pathway in plasma occurs *via* de-amidation of the C-terminus, confirmed by mass spectrometry and RP-HPLC analysis. The butyl modification also conferred resistance to metabolism when tested using S9 liver fraction from mouse. The optimum analogue responsive against the DMS79 cell line was the Butyl^4th^-NH_2_ pentapeptide, which revealed a concentration dependent increase in apoptosis: the level of late apoptotic cells rose from ∼36% at 2 μM to ∼96% at 6 μM, as determined by flow cytometry, compared to the unmodified peptide that showed no such effect. Concluding, the butyl substitutions offered the best perspective for high cytotoxicity, induction of apoptosis and metabolic compatibility thereby comprising an improved broad spectrum SP antagonist candidate for treatment of SCLC.

## Highlights

• 8 novel pentapeptide analogues of SPG with *N*-indole alkylation were synthesised.
• Butylation enhanced cytotoxicity against H69 and DMS79 cells; IC_50_ = 0.63–2.31 μM.
• DTrp(*N*-butyl) at 4th position (**16**) was effective at inducing apoptosis; 96% at 6 μM.
• Butylation enhanced peptides' stability in plasma and S9 liver fraction.
• To date, **16** & **19** (dibutylated) are the most potent peptides against SCLC.

## Graphical abstract

**Figure:**
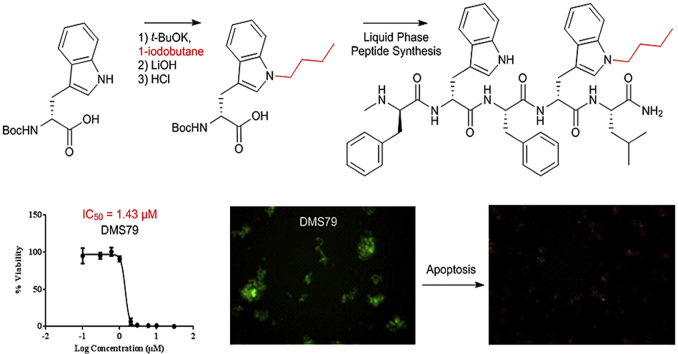

## 1 Introduction

Affecting mainly smokers, small cell lung cancer (SCLC) accounts for almost one quarter of all lung cancer deaths worldwide [1], [2], [3], [4]. First-line treatment, entailing a combination of chemotherapy (etoposide and cisplatin or carboplatin) and radiotherapy, is initially efficacious but then short-lived [3], [5]. The vast majority of patients relapse and develop resistance [3], [6]. Moreover, SCLC manifests early widespread development of metastases, often before the patient has been diagnosed with lung cancer [7]. Hence, it remains one of the most aggressive form of cancer and any new advances in developing chemotherapeutics have made little impact on patient outcome [1]. Existing cytotoxic agents that target a highly specific receptor suffer from rendering the cells chemo-resistant. To overcome this obstacle, scientific approaches based on inhibiting multi-targets are needed to alleviate drug resistance and achieve better efficacy.

SCLC is a pulmonary neuroendocrine carcinoid tumour. These cells produce a wide variety of neuropeptide growth factors and their cognate receptors. Several neuropeptide growth factors, including gastrin releasing peptide (GRP), neuromedin-B, gastrin, cholecystokinin, neurotensin, vasopressin, galanin and bradykinin, have been implicated in self-promoting tumour growth by acting through autocrine growth loops [8], [9], [10]. For example, they cause intracellular Ca^2+^ mobilisation that activates further signal transduction pathways [11], [12], [13]. In addition, the expression of these neuropeptide receptors increases as cells become resistant to first line chemotherapy [14]. Therefore, it is conceivable to interfere with the growth of SCLC using drugs which may block the action of mitogenic neuropeptides. Antagonists, referred to as substance P (SP) analogues, that intercept multiple neuropeptide signalling pathways to inhibit the proliferation of SCLC have been explored for their potential as broad spectrum anticancer agents. These SP analogues are well recognised competitive inhibitors of the mitogenic effects of several different neuropeptides. Among these analogues are: [D-Arg^1^,DPhe^5^,D-Trp^7,9^,Leu^11^]SP (SPD) [15], [16], [17], [Arg^6^,D-Trp^7,9^,MePhe^8^]SP (6–11) (SPG) [17], [18], [19], [D-Arg^1^,D-Trp^5,7,9^,Leu^11^]SP [20], NY3460 and NY3521 [21], and [D-Arg^1^,D-Trp^5,7,9^,D-Leu^10^,Leu^11^]SP-OH [22]. The exact mode of action of the SP analogues remains unclear and may involve several G-protein coupled receptors (GPCRs), for example, GRP receptor, vasopressin receptor and tachykinin NK-1 receptor [15], [17], [19], [23].

Cancer stem-like cells (CSC) belong to a small subpopulation of persistent cancer cells with acquired resistance to standard chemotherapy and research [24] points to CSCs playing an important role in the pathogenesis of SCLC. CD133 is a known bio-marker of CSC in several cancers. Sarvi and co-workers [22] found that CD133 positive cells have a higher level of GRP and vasopressin neuropeptide receptors and yet these cells also exhibit increased sensitivity to an SP analogue which is related in sequence to SPG. SPG is the only peptide to have been tested on human subjects in a phase 1 clinical trial [25]. These findings suggest a potential for dual efficacy by not only intercepting tumour growth *per se*, but also overcoming drug resistance. While many synthetic SP analogues have been designed and tested on a number of SCLC cell lines and tumour models [15], [16], [17], [18], [19], [20], [21], [22], the *in vitro* potency and hence *in vivo* tumour response at low doses remains far from satisfactory.

Recently, we developed a novel peptide derived from SPG but with a shorter amino acid sequence, 5-mer-NH_2_ (**1**) (DMePhe-DTrp-Phe-DTrp-Leu-NH_2_), with IC_50_ values in the range of 23–31 μM against H69 (chemo-naive SCLC cell line) and DMS79 (SCLC cell line originating from patient treated with chemotherapeutics and radiation therapy), superior to SPG [26]. Cytotoxicity was greatly enhanced by chemical *tert*-prenyl modification on the indole nitrogen (N^ind^) of the 4th d-tryptophan (D-Trp) residue to comprise *tert*-Prenyl^4th^-NH_2_ (**2**) (DMePhe-DTrp-Phe-DTrp(*N*-*tert*-prenyl)-Leu-NH_2_) [26]. The latter compound exhibited IC_50_ values of 2.84 ± 0.14 μM and 4.37 ± 0.44 μM on H69 and DMS79 cells, respectively [26]. Under the same conditions, SPG did not show cytotoxicity up to the maximum tested concentration of 30 μM on either cell line [26]. Using DMS79 xenografts and applying a low dose of 1.5 mg/kg, a 30% reduction in tumour volume was evident [26]. The objective of this research is to further enhance the cytotoxicity of these peptide analogues. Hence, inspired by the remarkable effect of the introduction of the *tert*-prenyl moiety to increase the potency of the peptide sequence against SCLC, we aimed to optimise the lead structure by considering increasing lengths of aliphatic alkyl modifications on N^ind^ of the D-Trp residues. Here, we have generated a further eight pentapeptides and explored the possibility of having the 2nd D-Trp residue modified. The most cytotoxic candidates were tested for their stability in plasma and S9 liver fraction from mouse before assessing their ability to induce apoptosis.

## 2 Results and discussion

### 2.1 Synthesis of N^ind^-alkylated d-tryptophan derivatives

**Scheme 1:**
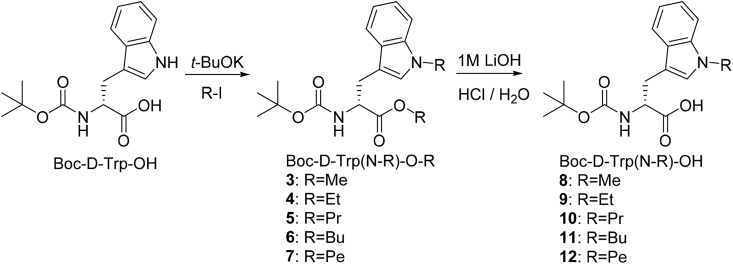
Synthesis of the carboxylic acid derivatives of N^ind^-alkylated Boc-D-Tryptophan (**8**–**12**).

The syntheses of N^ind^-alkylated D-Trp derivatives are illustrated in Scheme 1. The *tert*-butyloxycarbonyl D-Trp (Boc-D-Trp-OH) was treated with two equivalents of potassium *tert*-butoxide to de-protonate N^ind^ and the carboxylic acid, followed by reaction with alkyl (methyl, ethyl, propyl, butyl and pentyl) iodide. This resulted in alkylation of N^ind^ whilst also forming the alkyl ester (Boc-D-Trp(N-R)-O-R) (**3**–**7**). Subsequently, the ester was hydrolysed forming the alkylated building blocks required for peptides synthesis (Boc-D-Trp(N-R)-OH) (**8**–**12**). The structures were confirmed by ^1^H and ^13^C NMR spectroscopy and mass spectrometry (MS) as reported in the experimental, with NMR spectra provided in the Supplementary Data (SD) (Fig. S1-S11c).

The ^1^H NMR (400 MHz, CDCl_3_) spectra for the modified derivatives showed the disappearance of the N^ind^-H (observed at *δ*: 8.09 in the un-modified Boc-D-Trp-OH - Fig. S1), and the appearance of the peaks corresponding to the added alkyl groups. For example, the spectrum for **3** showed the appearance for two peaks corresponding to the two added methyl groups at 3.75 and 3.69 ppm, and the disappearance of the N^ind^-H peak (Fig. S2a). The spectrum for the carboxylic acid (**8**) showed only a single peak for the methyl group on N^ind^ at 3.74 ppm (Fig. S7a).

### 2.2 Peptides synthesis

**Fig. 1:**
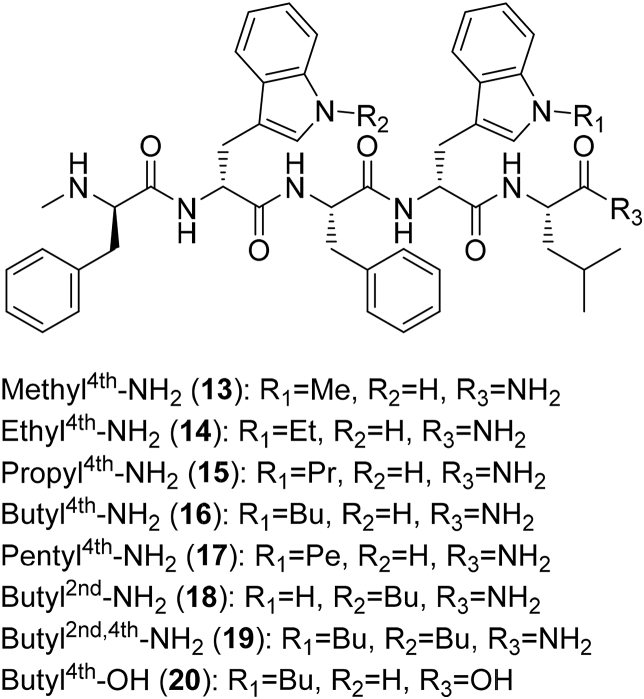
Structures of the novel peptides (**13**–**20**) synthesised by LPPS.

**Scheme 2:**
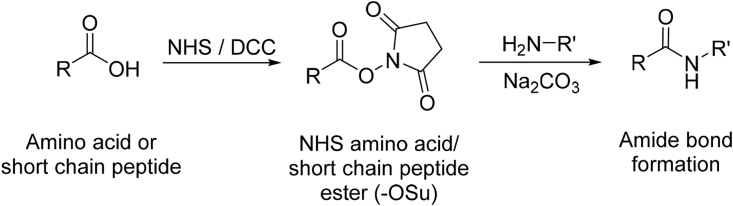
Synthesis of *N*-hydroxysuccinimide (NHS) amino acid/peptide ester utilized in the synthesis of peptides **13**–**20**. R = -CHR_1_-NH_2_/short peptide, R’ = -CHR_2_-COOH, R_1_/R_2_ = side chains.

The synthesis of peptides (**13–20**/Fig. 1) was completed using liquid phase peptide synthesis (LPPS) as previously described [26]. The procedure entailed activating the free carboxylic group to produce *N*-hydroxysuccinimide (NHS) amino acid ester (or short chain NHS peptide ester after amino acids coupling), followed by coupling with free amino acid (Scheme 2). Peptides were purified by reversed phase (RP)-HPLC (>95% purity) and structures (**13**–**20**) were verified by MS (data in experimental section and RP-HPLC chromatograms in Fig. S12-S19).

### 2.3 Cell viability assays

Cell viability was evaluated by treating H69 and DMS79 cells with peptides **13**–**20** in the 0.1–30 μM range for 48 h, with subsequent use of the resazurin dye cell viability assay. IC_50_ values (Table 1**)** for all the peptides were derived from the dose response curves (Fig. S24). Table 1 also presents the IC_50_ values obtained for **1** and **2** previously [26].

**Table 1:** Cytotoxicity (IC_50_ ± SE) of **1**, **2** and **13**–**20** on H69 and DMS79 cell lines after treatment for 48 h.

Peptide	H69 Cells	DMS79 Cells
	IC_50_ ± SE (μM) n = 3	IC_50_ ± SE (μM) n = 3
**1**	30.74 ± 0.30	23.00 ± 2.07
**2**	2.84 ± 0.14	4.37 ± 0.44
**13**	3.98 ± 0.22	4.03 ± 0.31
**14**	1.83 ± 0.03	1.92 ± 0.03
**15**	1.57 ± 0.10	1.91 ± 0.03
**16**	**1.01** ± 0.02	**1.43** ± 0.14
**17**	1.80 ± 0.10	2.37 ± 0.06
**18**	4.05 ± 0.17	3.88 ± 0.15
**19**	**0.63** ± 0.05	**2.31** ± 0.12
**20**	>30	>30

In the first series of peptides (**13**–**17**), the N^ind^-alkylated D-Trp is located near the C-terminus of the peptide sequence (4th residue), analogous to the previous lead sequence (**2**) [26] that was modified with *tert*-prenyl at the same position. Benchmarking the observed IC_50_ values (Table 1) against **2**, all of the N^ind^-alkyl modified peptides in this series (**13**–**17**) showed higher cytotoxicity on both cell lines, except for **13** (N^ind^-methyl D-Trp) on H69, which was ∼1.4 times less active. There is a general trend in improved cytotoxicity as the alkyl chain length increases from C1 to C4. The best analogue comprises N^ind^-butyl D-Trp at the 4th residue (**16**). Overall, **16** was ∼3 fold more cytotoxic, *in vitro*, on both cell lines than **2**.

To explore whether D-Trp on the 2nd residue of the sequence made the peptide more cytotoxic, **18** was synthesised and screened. This positional change of N^ind^-butyl D-Trp in the peptide sequence reduced the potency by approximately 3–4 fold, in comparison to **16**, on both cell lines. Its cytotoxicity was comparable to the methylated analogue (**13**). However, **16** and **18** are considered potent in comparison to **1** that has un-modified D-Trp residues (Table 1). In view of this, a further peptide was designed to take advantage of observed enhanced cytotoxicity at both D-Trp positions relative to **1**. Peptide **19**, with both the 2nd and the 4th residues of D-Trp modified with the N^ind^-butyl group, resulted in the highest cytotoxicity of all peptides on the H69 cell line. Indeed, for the first time, a sub-micromolar value has been achieved for any SP analogue for SCLC treatment. DMS79 cell line originates from a patient with SCLC who had undergone treatment with cytoxan, vincristine, methotrexate and radiation therapy [26]. Such treatment could reduce the sensitivity of the cell line to chemotherapy. The dual modified peptide was found to retain higher cytotoxicity against DMS79 compared to **2**, albeit with a ∼1.6 fold reduction compared to **16**.

A further analogue of the most potent peptide (**16**) against DMS79 was produced to study the influence of the C-terminus amide group. Peptide **20** was thus synthesised as a free carboxylic acid and screened. The de-amidation of the C-terminus completely inactivated the peptide against both cell lines, with no growth inhibition at 30 μM. This indicates that the C-terminal amide is crucial for cytotoxicity.

Broad spectrum antagonists may have multiple targets belonging to the G-protein linked receptors, many of which are membrane bound with unknown crystal structures. In the absence of any specific target and structural information, it remains difficult to rationalise the exact role played by the N^ind^-butyl and the C-terminal amide groups on the basis of molecular modelling. However, since the cytotoxicity peaked at the same chain length (butyl) for both the cell lines, it could be argued that there is some common binding site requiring strong hydrophobic interaction with an alkyl chain and such site has steric constraints to optimally accommodate butyl group. This may also explain the loss of activity upon de-amidation, as the carboxylic group in its ionised form, would significantly reduce the hydrophobicity gain from the alkyl chain that is critical to render the peptide cytotoxic. The hydrophilic-lipohilic balance is particularly effective as the 4th residue is close to the peptide's C-terminus.

### 2.4 The stability of peptides **2**, **16**, **19** and **20** in plasma and S9 liver fraction from mouse

**Fig. 2:**
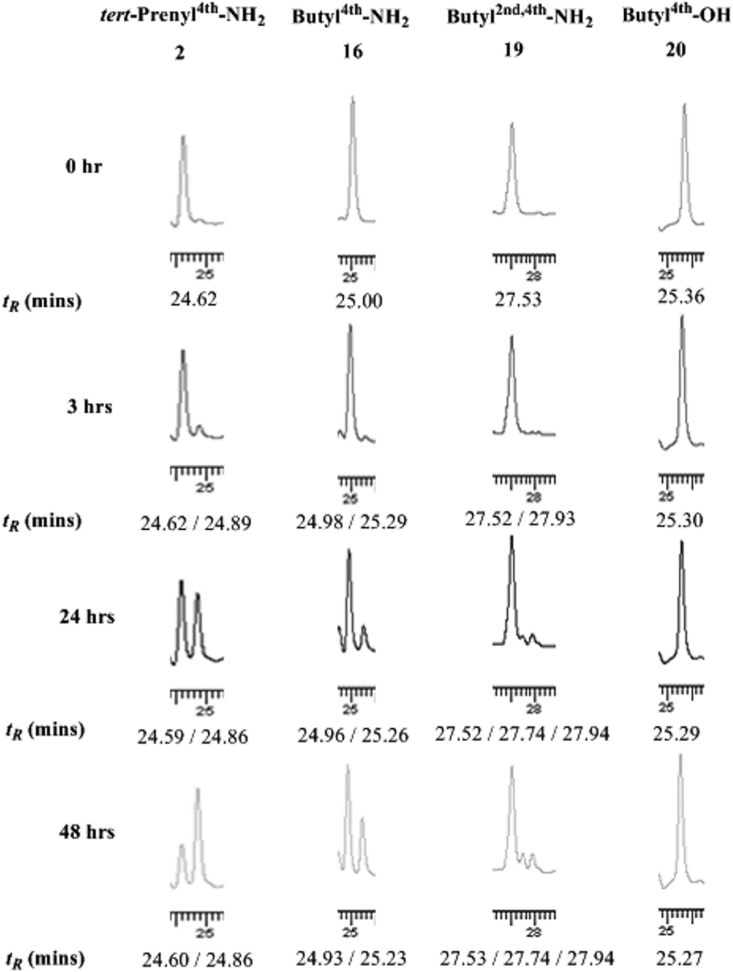
Sections of RP-HPLC chromatograms obtained from the plasma stability studies for **2**, **16**, **19** and **20**. Chromatograms are from 0, 3, 24 and 48 h post-incubation of peptides at 37 °C with retention times (*t*_*R*_) of peaks observed presented beneath the chromatograms. The full chromatograms are presented in SD (Fig. S25-S28).

When optimising for functional activity there is also a need to assess whether the desired modification will negatively affect the stability of the peptide. Characterisation of the metabolism of peptides is useful to improve their stability *in vivo*. The finding that a carboxylic acid group, instead of the usual amide, at the C-terminus inactivates the cytotoxicity of **16** (results of **20** - Table 1) may compromise its use *in vivo* as protein de-amidation reactions are widespread in plasma. Peptides **16** and **19**, being the most cytotoxic peptides, were exposed to the conditions of *in vitro* metabolism in plasma. Peptide **20** lacking the amide group was used as a control. Peptides **16**, **19** and **20** were incubated in neat mouse plasma at 37 °C for 48 h. Peptide **2**, previously tested over a shorter incubation time [26], was included for benchmarking purpose. Relevant sections of typical chromatograms obtained are shown in Fig. 2.

**Fig. 3:**
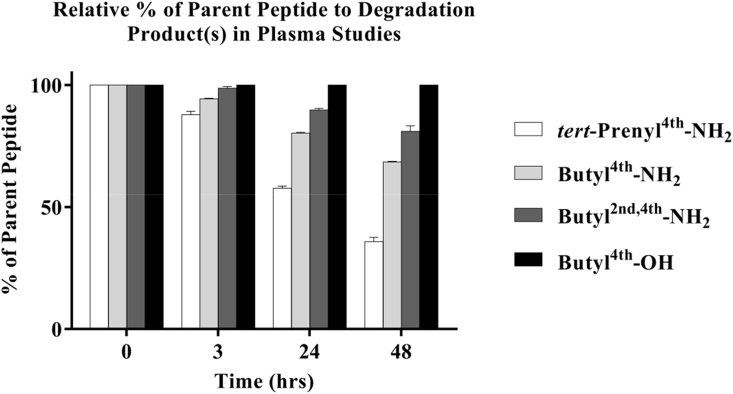
Bar-chart presentation for the relative percentage (±SEM) of **2**, **16**, **19**, and **20** to degradation product(s) in plasma studies at 0, 3, 24 and 48 h post-incubation; n = 3.

The chromatograms for **2**, **16** and **19** showed a gradual loss of the parent peptide with the concomitant increase in intensity of closely eluting peak(s) with a slightly higher retention time (*t*_*R*_) suggesting time dependent degradation in plasma. Quantitative assessment (Fig. 3) revealed that the butylated sequences (**16** and **19**) were far more stable with >80% integrity compared to the *tert*-prenylated (**2**) form, which degraded by almost ∼42%, after 24 h exposure. The di-butylated peptide (**19**) was the most stable with 81% integrity after 48 h exposure to neat plasma. Peptide **20**, similar to **16** but with a carboxylic acid at the C-terminus, showed no sign of degradation. The percentages of the parent peptides remaining after each time point in plasma studies are presented in SD in Table S1.

**Fig. 4:**
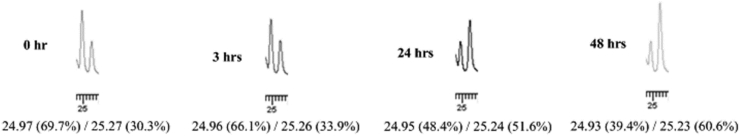
Sections of RP-HPLC chromatograms for the plasma stability study performed on the mixture containing **16** and a lower amount of **20**, over time (0–48 h). *t*_*R*_ and percentage of each peak is presented beneath each chromatogram. The full chromatograms are presented in SD (Fig. S29).

From the closely matching retention times for the degradation product of the amidated peptide **16** (*t*_*R*_ = 25.23 mins after 48 h) and that of the carboxylic acid peptide **20** (*t*_*R*_ = 25.27 mins after 48 h), it seemed plausible to infer that the degradation of **16** could arise by a de-amidation reaction in plasma. To substantiate this further, both peptides (**16** and **20**) were mixed and processed using identical sampling procedure. The amount of **20** in the mixture was kept at 2.5 times lower than **16** to simulate higher level of the amidated product as noted for the 48 h plasma treated sample (Fig. 2, bottom RP-HPLC trace section for **16**) and to help assign the two peaks following elution. The mixture of the two peptides was exposed to neat plasma as above and the samples analysed. Fig. 4 clearly showed that the de-amidated form had a peak of lower intensity which eluted later than its amide form. The relative percentage of each compound and their specific *t*_*R*_ is also presented beneath the chromatogram sections. The profiles and degradation pattern appears similar to those obtained for the degradation of **16** (Fig. 2) having almost identical *t*_*R*_ values. The peak area assigned to the de-amidated peptide (**20**) increases concurrently with a decline of the amide peptide peak (**16**). The process of degradation in plasma is thus likely to be due to de-amidation of the C-terminus residue.

Samples at 0 and 48 h incubation with plasma (Fig. 4) were analysed by MS. The MS spectra (presented in SD) also supports the de-amidation of **16** to give **20**. The most abundant ion observed for the 48 h sample was for the de-amidated compound at *m/z*: 868.5 (Fig. S30) compared to *m/z*: 867.5 (Fig. S30) for the amidated compound for the 0 h sample. Results were similar for the plasma stability study for **2** also showing a one unit higher mass after incubation in plasma. The predominant ion at *m/z*: 880.5 (Fig. S31) was present at 48 h for the de-amidated compound and at 0 h for the parent amide peptide (**2**) at *m/z*: 879.5 (Fig. S31). Peptide **20**, devoid of a C-terminal amide, showed the same predominant ion at *m/z*: 868.5 (Fig. S32) indicating complete stability in plasma, in agreement with the RP-HPLC data (Fig. 2). For **16** and **19**, the MS results showed a predominant ion for the parent amidated form at 0 and 48 h (Fig. S33 and S34). This is consistent with the observed RP-HPLC profiles (Fig. 2) also indicating that the major component remaining is the native butylated and amidated sequences. The N^ind^-butyl modifications have thus significantly reduced the susceptibility of these analogues to plasma, to a point that there is little peptide degradation up to 24 h.

Peptides **16** and **19** were further subjected to the S9 liver fraction from mouse in the presence of cofactors [27] for a period of 3 h. This would give further insight into the stability of the peptides towards Phase I and Phase II metabolising enzymes. Peptides **16** and **19** were shown to be completely stable in S9 liver fraction (Fig. S35 and S36).

Our results are consistent with another related SP analogue known as SPD, which most likely targets the same receptors as SPG [28]. One of the two major metabolites for SPD (DArg-Pro-Lys-Pro-DPhe-Gln-DTrp-Phe-DTrp-Leu-Leu-NH_2_) was isolated and confirmed by MS as the C-terminus de-amidated product (DArg-Pro-Lys-Pro-DPhe-Gln-DTrp-Phe-DTrp-Leu-Leu-OH) as a result of serine protease action. The *in vitro* biological activity of this metabolite was poor as the action of neuropeptides, bombesin, vasopressin or bradykinin, could not be antagonised with it. This is in contrast to the antagonist effect observed with the amidated parent peptide when using the same neuropeptides. It was implied that receptors for these growth factors could be more selective in binding to the C-terminus structure of their antagonists. Our results support this proposal as modifications near the C-terminus, D-Trp at 4th position, were identified as the most effective sites to maximise the cytotoxicity and resistance to plasma and S9 liver fraction degradation. Therefore the hypothesis made by Jones et al. [28] that “development of more potent broad-spectrum antagonists may be possible by slight modifications of the C-terminus” has now been substantiated with our analogues in this study.

### 2.5 Assessment of apoptosis

#### 2.5.1 Acridine orange/ethidium bromide dual staining

**Fig. 5:**
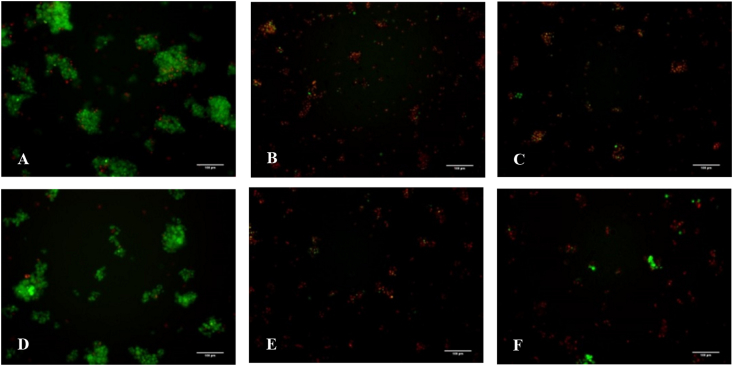
H69 cells (top) and DMS79 cells (bottom), untreated (**A** and **D**) and incubated with 6 μM of **16** (**B** and **E**) and **19** (**C** and **F**) for 48 h in complete media in 96-well plates. The cells were stained with 5 μl AO/EB mix and viewed under an inverted fluorescence microscope. The scale bar on each photomicrograph is 100 μm.

The most cytotoxic peptides, singly (**16**) and di-butylated (**19**) peptides, were selected for testing their ability to induce apoptosis in H69 and DMS79 cell lines. Photomicrographs of cells stained with acridine orange (AO) and ethidium bromide (EB) are presented in Fig. 5. Untreated cells (Fig. 5 A and D) showed predominantly green fluorescence due to intact plasma membrane allowing AO staining only [29]. However, above the IC_50_ values for both peptides at 6 μM concentration (Fig. 5 B,C,E and F) of peptides, mainly red/orange fluorescence attributed to loss of plasma membrane integrity is seen [29]. Hence, EB gains entry into cells to intercalate with the DNA, highlighting the late apoptotic and necrotic cells [29], [30]. In the latter case (at 6 μM) cell shrinkage was also observed when compared to the controls, suggesting apoptosis [31]. A few bright green regions were still observed on cells treated with 6 μM, suggesting chromatin condensation of cells undergoing apoptosis [30].

#### 2.5.2 Annexin V conjugate and flow cytometric analysis

The novel potent N^ind^-butylated peptide, **16**, and its unmodified version **1** [26] were incubated for 24 h with DMS79 cells and double stained using Annexin V Fluor^®^ 555 (AnnV) conjugate and SYTOX^®^ Blue (SyB) dead cell stain [29], [32]. The former stain binds to phosphatidyl serine (PS) residues that become exposed at the outer surface of the plasma membrane of cells undergoing apoptosis. DMS79 cell line was selected as a variant cell line model more resistant to chemotherapy since it is established from a SCLC patient treated with three different chemotherapeutics and radiation therapy [26]. Peptide **16** was the most cytotoxic against this cell line (Table 1).

**Fig. 6:**
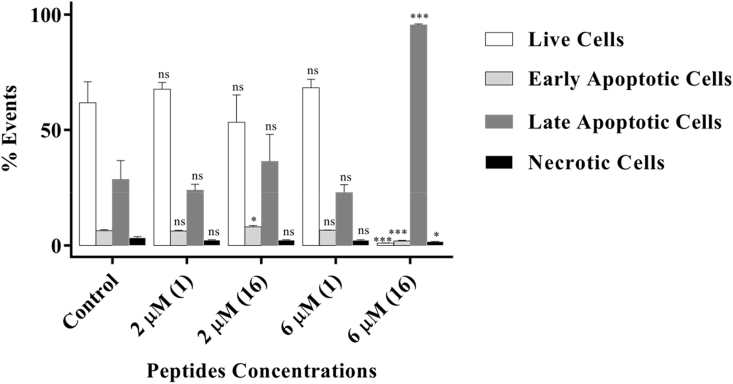
A bar-chart presentation for the flow cytometric analysis showing the % events (±SEM) of live, early apoptotic, late apoptotic and necrotic cells for DMS79 cells treated with 2 and 6 μM of peptides **1** and **16**, compared to untreated sample; (n = 3); *(P < 0.05); ***(P < 0.0005).

Quantitative assessment of apoptosis was evaluated at low (2 μM) and high (6 μM) peptide concentrations using flow cytometry. A bar-chart (Fig. 6) presents the levels of cells classified into four stages; live cells (AnnV-/SyB-), early apoptotic cells (AnnV+/SyB-), late apoptotic cells (AnnV+/SyB+) and necrotic cells (AnnV-/SyB+). Dot blots are presented in the SD (Fig. S37). Apart from a slight increase in live cells, treatment with **1** had similar levels of cells in each of the four stages as the untreated sample, irrespective of the concentrations used. This is an expected observation as **1** has a high IC_50_ value (23 μM) [26] on this cell line and will be non-cytotoxic at these concentrations. In contrast, the singly butylated peptide (**16**) caused highly significant (P < 0.0005) apoptosis in a concentration dependent manner. Even at a low concentration of peptide (2 μM), a small increase (P < 0.05) of early apoptotic cells was observed relative to the control sample. Raising the concentration of **16** to 6 μM caused the level of late apoptotic cells, to increase massively up to 95.5% (Fig. S37 E − top right quadrant) and concurrently the level of viable cells (Fig. S37 E − bottom left quadrant) dropped to 1.0%. These results show that the optimised peptide **16** was highly effective to induce cell death in what would otherwise be a very difficult cell line (DMS79) to treat using conventional chemotherapeutics.

## 3 Conclusion

Novel pentapeptides based on the short SPG sequence were synthesised by incorporating D-Trp derivatives modified with N^ind^-alkyl chains. Screening against SCLC cell lines revealed that the N^ind^-butyl substituent provided the most cytotoxic peptides with improved resistance to de-amidation by plasma and metabolism in the S9 liver fraction. A single N^ind^-butyl modification on the 4th D-Trp residue enhanced the cytotoxicity by a ∼3 fold with H69 and DMS79 cells, compared to our recently discovered sequence (*tert*-Prenyl^4th^-NH_2_) [26] where N^ind^-*tert*-prenyl was incorporated on the indole ring. The most active pentapeptide against the human SCLC cell line DMS79 (derived from a patient who had been treated with chemotherapeutics and radiotherapy), was **16** (DMePhe-DTrp-Phe-DTrp(*N*-butyl)-Leu-NH_2_). Given that this peptide sequence was also metabolically stable for sufficient time, future tumour regression studies for SCLC are planned. This may provide an improved treatment for chemo-resistant SCLC, through the broad spectrum action of this peptide analogue to inhibit a number of receptors and multiple signalling pathways [33], [34].

## 4 Experimental

### 4.1 Materials and instrumentation for the synthesis of N^ind^-alkylated d-tryptophan derivatives

Chemicals and solvents were obtained from Novabiochem, Sigma-Aldrich and Fisher Scientific. Silica gel, ZEOprep 60/40–63 μm, was obtained from Apollo Scientific. Thin Layer Chromatography (TLC) plates, TLC Silica gel 60 F_254_ Aluminium sheets, were obtained from Merck Millipore. Reaction progression was monitored by TLC and spots on TLC plates were visualised using UV Mineralight lamp (254/365) UVGL-58. ^1^H and ^13^C NMR Spectroscopy were performed using a Bruker Avance 400 MHz (^1^H) and 75 MHz (^13^C) spectrometer and chemical shifts (*δ*) are quoted in parts per million and referenced to solvent residual peak. NMR spectra were generated using Topspin 2.1 software. Electrospray Ionisation MS (ESIMS) (Waters SQD-2 Single Quadrupole Mass Spectrometer attached to Acquity UHPLC) and Atmospheric Pressure Chemical Ionisation MS (APCIMS) (Agilent Technologies 6120 Quadrupole LC/MS) were performed at the School of Chemistry, the University of Manchester and molecular ions peaks are reported as mass/charge (*m/z*) ratios. Melting points (mp) were measured using a Stuart Scientific melting point apparatus SMP10.

#### 4.1.1 Boc-D-Trp(*N*-methyl)-O-methyl **(3)**

A solution of *t*-BuOK (0.74 g, 6.58 mmol) in dry tetrahydrofuran (THF) (10 ml) was added dropwise to a solution of Boc-D-Trp-OH (1.0 g, 3.29 mmol) in dry *N,N*-dimethylformamide (10 ml) under N_2_ atmosphere at 0 °C. The mixture was stirred for 20 min at room temperature (RT). Iodomethane (410 μl, 6.58 mmol) was then added to the above mixture under N_2_ atmosphere at 0 °C. The mixture was stirred at RT for 8 h. The reaction completion was checked by TLC (ethyl acetate/hexane, 1:1). Citric acid aqueous solution (30% w/v) (200 ml) was then added and the mixture was extracted with ethyl acetate (4 × 50 ml). The organic layers were combined, dried over anhydrous sodium sulphate (Na_2_SO_4_), and concentrated under reduced pressure. The crude product was purified by flash column chromatography (ethyl acetate/hexane, 1:4), to give 0.75 g (68.7%) of **3** as a yellow oil. ^1^H NMR (400 MHz, CDCl_3_) *δ*: 7.54 (d, 1H, *J* 7.6 Hz, Ar-H), 7.29 (d, 1H, *J* 8.0 Hz, Ar-H), 7.23 (dd ∼ t, 1H, *J* 7.6 Hz, Ar-H), 7.11 (dd ∼ t, 1H, *J* 7.4 Hz, Ar-H), 6.86 (s, 1H, Ind-2-H), 5.07 (br-d, 1H, *J* 7.6 Hz, NHBoc), 4.64 (dd, 1H, *J* 13.2, 5.2 Hz, CH), 3.75 (s, 3H, N-CH_3_ on Ind), 3.69 (s, 3H, OCH_3_ ester), 3.33–3.24 (m, 2H, CH_2_), 1.44 (s, 9H, *t*-Bu). ^13^C NMR (75 MHz, CDCl_3_, assignments made using DEPT-135) *δ*: 172.9 (C, C12), 155.4 (C, C13), 137.1 (C, Ar-C), 128.3 (C, Ar-C), 127.6 (CH, Ar-C), 121.9 (CH, Ar-C), 119.2 (CH, Ar-C), 119.0 (CH, Ar-C), 109.4 (CH, Ar-C), 108.7 (C, Ar-C), 79.9 (C, C14), 54.4 (CH, C11), 52.3 (OCH_3_ ester), 32.8 (N-CH_3_ on Ind), 28.4 (3 × CH_3_, C15, C16, C17), 28.0 (CH_2_, C10). MS (ESI) *m/z* [M+Na]^+^ 355.2. Accurate mass calculated for C_18_H_24_N_2_O_4_Na: 355.1628. Found: MS (ESI) 355.1637.

#### 4.1.2 Boc-D-Trp(*N*-ethyl)-O-ethyl **(4)**

The method of synthesis was similar to that described for **3**, except that iodoethane (526 μl, 6.58 mmol) was used. A yellow oil (0.67 g, 56.6%) of **4** was isolated after flash chromatography. ^1^H NMR (400 MHz, CDCl_3_) *δ*: 7.56 (d, 1H, *J* 7.6 Hz, Ar-H), 7.31 (d, 1H, *J* 8.4 Hz, Ar-H), 7.21 (dd ∼ t, 1H, *J* 8.0 Hz, Ar-H), 7.10 (dd ∼ t, 1H, *J* 7.4 Hz, Ar-H), 6.93 (s, 1H, Ind-2-H), 5.07 (br-d, 1H, *J* 7.6 Hz, NHBoc), 4.62 (dd, 1H, *J* 13.6, 5.6 Hz, CH), 4.13 (q, 2H, *J* 7.1 Hz, CH_2_CH_3_), 4.13 (q, 2H, *J* 7.3 Hz, CH_2_CH_3_), 3.32–3.23 (m, 2H, CH_2_), 1.44 (s, 9H, *t*-Bu), 1.44 (t, 3H, *J* 7.2 Hz, 3H of OCH_2_CH_3_ ester), 1.20 (t, 3H, *J* 7.2 Hz, N-CH_2_CH_3_ on Ind). ^13^C NMR (75 MHz, CDCl_3_, assignments made using DEPT-135) *δ*: 172.5 (C, C12), 155.4 (C, C13), 136.1 (C, Ar-C), 128.6 (C, Ar-C), 125.8 (CH, Ar-C), 121.7 (CH, Ar-C), 119.1 (2 × CH, Ar-C), 109.4 (CH, Ar-C), 109.0 (C, Ar-C), 79.8 (C, C14), 61.3 (CH_2_, OCH_2_CH_3_ ester), 54.5 (CH, C11), 40.9 (CH_2_, N-CH_2_CH_3_ on Ind), 28.5 (3 × CH_3_, C15, C16, C17), 28.2 (CH_2_, C10), 15.5 (CH_3_, N-CH_2_CH_3_ on Ind), 14.2 (CH_3_, CH_2_CH_3_ on ester). MS (ESI) *m/z* [M+K]^+^ 399.2. Accurate mass calculated for C_20_H_28_N_2_O_4_K: 399.1681. Found: MS (ESI) 399.1673.

#### 4.1.3 Boc-D-Trp(*N*-propyl)-O-propyl **(5)**

The method of synthesis was similar to that described for **3**, except that 1-iodopropane (642 μl, 6.58 mmol) was used. A yellow oil (0.75 g, 58.8%) of **5** was isolated after flash chromatography. ^1^H NMR (400 MHz, CDCl_3_) *δ*: 7.56 (d, 1H, *J* 8.0 Hz, Ar-H), 7.30 (d, 1H, *J* 8.0 Hz, Ar-H), 7.20 (dd ∼ t, 1H, *J* 7.6 Hz, Ar-H), 7.10 (dd ∼ t, 1H, *J* 7.4 Hz, Ar-H), 6.91 (s, 1H, Ind-2-H), 5.07 (br-d, 1H, *J* 7.6 Hz, NHBoc), 4.63 (dd, 1H, *J* 13.2, 5.6 Hz, CH), 4.05–3.99 (m, 4H, CH_2_CH_2_CH_3_ on Ind and ester), 3.30 (dd, 1H, *J* 14.2, 5.0 Hz, 10-CH_A_), 3.26 (dd, 1H, *J* 14.6, 5.0 Hz, 10-CH_B_), 1.84 (sextet, 2H, *J* 7.2 Hz, N-CH_2_CH_2_CH_3_ on Ind), 1.59 (sextet, 2H, *J* 7.0 Hz, OCH_2_CH_2_CH_3_ ester), 1.44 (s, 9H, *t*-Bu), 0.91 (t, 3H, *J* 7.4 Hz, OCH_2_CH_2_CH_3_ ester), 0.86 (t, 3H, *J* 7.4 Hz, N-CH_2_CH_2_CH_3_ on Ind). ^13^C NMR (75 MHz, CDCl_3_, assignments made using DEPT-135) *δ*: 172.5 (C, C12), 155.4 (C, C13), 136.4 (C, Ar-C), 128.5 (C, Ar-C), 126.6 (CH, Ar-C), 121.7 (CH, Ar-C), 119.1 (2 × CH, Ar-C), 109.5 (CH, Ar-C), 108.8 (C, Ar-C), 79.8 (C, C14), 66.9 (CH_2_, OCH_2_CH_2_CH_3_ ester), 54.5 (CH, C11), 48.0 (CH_2_, N-CH_2_CH_2_CH_3_ on Ind), 28.4 (3 × CH_3_, C15, C16, C17), 28.2 (CH_2_, C10), 23.6 (CH_2_, N-CH_2_CH_2_CH_3_ on Ind), 22.0 (CH_2_, OCH_2_CH_2_CH_3_ ester), 11.6 (CH_3_, N-CH_2_CH_2_CH_3_ on Ind), 10.4 (CH_3_, OCH_2_CH_2_CH_3_ ester). MS (ESI) *m/z* [M+Na]^+^ 411.2. Accurate mass calculated for C_22_H_32_N_2_O_4_Na: 411.2254. Found: MS (ESI) 411.2248.

#### 4.1.4 Boc-D-Trp(*N*-butyl)-O-butyl **(6)**

The method of synthesis was similar to that described for **3**, except that 1-iodobutane (749 μl, 6.58 mmol) was used. A yellow oil (1.03 g, 75.3%) of **6** was isolated after flash chromatography. ^1^H NMR (400 MHz, CDCl_3_) *δ*: 7.55 (d, 1H, *J* 7.6 Hz, Ar-H), 7.30 (d, 1H, *J* 8.4 Hz, Ar-H), 7.20 (dd ∼ t, 1H, *J* 7.6 Hz, Ar-H), 7.10 (dd ∼ t, 1H, *J* 7.2 Hz, Ar-H), 6.90 (s, 1H, Ind-2-H), 5.07 (br-d, 1H, *J* 8.0 Hz, NHBoc), 4.63 (dd, 1H, *J* 13.6, 5.6 Hz, CH), 4.10–4.03 (m, 4H, CH_2_CH_2_CH_2_CH_3_ on Ind and ester), 3.32–3.23 (m, 2H, CH_2_), 1.79 (pentet, 2H, *J* 7.3 Hz, N-CH_2_CH_2_CH_2_CH_3_ on Ind), 1.53 (pentet, 2H, *J* 7.2 Hz, OCH_2_CH_2_CH_2_CH_3_ ester), 1.44 (s, 9H, *t*-Bu), 1.30 (sextet, 4H, *J* 7.5 Hz, CH_2_CH_2_CH_2_CH_3_ on Ind and ester), 0.94 (t, 3H, *J* 7.4 Hz, OCH_2_CH_2_CH_2_CH_3_ ester), 0.89 (t, 3H, *J* 7.4 Hz, N-CH_2_CH_2_CH_2_CH_3_ on Ind). ^13^C NMR (75 MHz, CDCl_3_, assignments made using DEPT-135) *δ*: 172.5 (C, C12), 155.4 (C, C13), 136.4 (C, Ar-C), 128.5 (C, Ar-C), 126.6 (CH, Ar-C), 121.7 (CH, Ar-C), 119.1 (2 × CH, Ar-C), 109.5 (CH, Ar-C), 108.8 (C, Ar-C), 79.8 (C, C14), 65.2 (CH_2_, OCH_2_CH_2_CH_2_CH_3_ ester), 54.5 (CH, C11), 46.1 (CH_2_, N-CH_2_CH_2_CH_2_CH_3_ on Ind), 32.4 (CH_2_, N-CH_2_CH_2_CH_2_CH_3_ on Ind), 30.6 (CH_2_, OCH_2_CH_2_CH_2_CH_3_ ester), 28.5 (3 × CH_3_, C15, C16, C17), 28.2 (CH_2_, C10), 20.3 (CH_2_, N-CH_2_CH_2_CH_2_CH_3_ on Ind), 19.1 (CH_2_, OCH_2_CH_2_CH_2_CH_3_ ester), 13.80 (CH_3_, N-CH_2_CH_2_CH_2_CH_3_ on Ind), 13.76 (CH_3_, OCH_2_CH_2_CH_2_CH_3_ ester). MS (ESI) *m/z* [M+K]^+^ 455.2. Accurate mass calculated C_24_H_36_N_2_O_4_K: 455.2307. Found: MS (ESI) 455.2308.

#### 4.1.5 Boc-D-Trp(*N*-pentyl)-O-pentyl **(7)**

The method of synthesis was similar to that described for **3**, except that 1-iodopentane (856 μl, 6.58 mmol) was used. A yellow oil (0.53 g, 36.3%) of **7** was isolated after flash chromatography. ^1^H NMR (400 MHz, CDCl_3_) *δ*: 7.54 (d, 1H, *J* 8.0 Hz, Ar-H), 7.30 (d, 1H, *J* 8.0 Hz, Ar-H), 7.19 (dd ∼ t, 1H, *J* 7.6 Hz, Ar-H), 7.09 (dd ∼ t, 1H, *J* 7.3 Hz, Ar-H), 6.90 (s, 1H, Ind-2-H), 5.06 (br-d, 1H, *J* 8.0 Hz, NHBoc), 4.62 (dd, 1H, *J* 13.6, 5.6 Hz, CH), 4.09–3.99 (m, 4H, CH_2_CH_2_CH_2_CH_2_CH_3_ on Ind and ester), 3.32–3.21 (m, 2H, CH_2_), 1.80 (pentet, 2H, *J* 7.3 Hz, N-CH_2_CH_2_CH_2_CH_2_CH_3_ on Ind), 1.55 (pentet, 2H, *J* 7.1 Hz, OCH_2_CH_2_CH_2_CH_2_CH_3_ ester), 1.43 (s, 9H, *t*-Bu), 1.37–1.21 (m, 8H, CH_2_CH_2_CH_2_CH_2_CH_3_ on Ind and ester), 0.90–0.86 (m, 6H, CH_2_CH_2_CH_2_CH_2_CH_3_ on Ind and ester). ^13^C NMR (75 MHz, CDCl_3_, assignments made using DEPT-135) *δ*: 172.6 (C, C12), 155.4 (C, C13), 136.3 (C, Ar-C), 128.5 (C, Ar-C), 126.5 (CH, Ar-C), 121.7 (CH, Ar-C), 119.1 (2 × CH, Ar-C), 109.5 (CH, Ar-C), 108.8 (C, Ar-C), 79.8 (C, C14), 65.6 (CH_2_, OCH_2_CH_2_CH_2_CH_2_CH_3_ ester), 54.5 (CH, C11), 46.4 (CH_2_, N-CH_2_CH_2_CH_2_CH_2_CH_3_ on Ind), 30.1 (CH_2_, N-CH_2_CH_2_CH_2_CH_2_CH_3_ on Ind), 29.3 (CH_2_, N-CH_2_CH_2_CH_2_CH_2_CH_3_ on Ind), 28.5 (CH_2_, OCH_2_CH_2_CH_2_CH_2_CH_3_ ester), 28.3 (3 × CH_3_, C15, C16, C17), 28.2 (CH_2_, C10), 28.1 (CH_2_, OCH_2_CH_2_CH_2_CH_2_CH_3_ ester), 22.5 (CH_2_, N-CH_2_CH_2_CH_2_CH_2_CH_3_ on Ind), 22.4 (CH_2_, OCH_2_CH_2_CH_2_CH_2_CH_3_ ester), 14.07 (CH_3_, N-CH_2_CH_2_CH_2_CH_2_CH_3_ on Ind), 14.04 (CH_3_, OCH_2_CH_2_CH_2_CH_2_CH_3_ ester). MS (ESI) *m/z* [M+H]^+^ 445.3. Accurate mass calculated for C_26_H_40_N_2_O_4_H: 445.3061. Found: MS (ESI) 445.3058.

#### 4.1.6 Boc-D-Trp(*N*-methyl)-OH **(8)**

An aqueous solution of 1 M LiOH (25 ml) was added to 0.7 g of **3** dissolved in THF (25 ml) and the reaction was monitored by TLC (ethyl acetate/hexane, 1:1). After completion of hydrolysis, the reaction was acidified by an aqueous solution of 1 M HCl and the mixture was extracted with ethyl acetate (3 × 50 ml). The organic layers were combined, washed with H_2_O (2 × 20 ml), dried over Na_2_SO_4_, concentrated under reduced pressure, and lyophilised to give 0.60 g (89.5%) of **8** as a white solid; mp: 154–156 °C. ^1^H NMR (400 MHz, CDCl_3_) *δ*: 7.59 (d, 1H, *J* 8.0 Hz, Ar-H), 7.29 (d, 1H, *J* 8.0 Hz, Ar-H), 7.23 (dd ∼ t, 1H, *J* 7.4 Hz, Ar-H), 7.11 (dd ∼ t, 1H, *J* 7.4 Hz, Ar-H), 6.90 (s, 1H, Ind-2-H), 5.03 (br-d, 1H, *J* 7.2 Hz, NHBoc), 4.7–4.6 (br-m, 1H, CH), 3.74 (s, 3H, N-CH_3_), 3.38–3.26 (m, 2H, CH_2_), 1.43 (s, 9H, *t*-Bu). ^13^C NMR (75 MHz, CDCl_3_, assignments made using DEPT-135) *δ*: 176.6 (C, C12), 155.8 (C, C13), 137.1 (C, Ar-C), 128.4 (C, Ar-C), 127.8 (CH, Ar-C), 122.0 (CH, Ar-C), 119.4 (CH, Ar-C), 119.0 (CH, Ar-C), 109.4 (CH, Ar-C), 108.5 (C, Ar-C), 80.4 (C, C14), 54.4 (CH, C11), 32.8 (CH_3_, N-CH_3_), 28.4 (3 × CH_3_, C15, C16, C17), 27.6 (CH_2_, C10). MS (ESI) *m/z* [M+Na]^+^ 341.1. Accurate mass calculated for C_17_H_22_N_2_O_4_Na: 341.1472. Found: MS (ESI) 341.1493.

#### 4.1.7 Boc-D-Trp(*N*-ethyl)-OH **(9)**

The method of synthesis was similar to that described for **8**; 1 M LiOH was added to 0.6 g of **4** to give 0.51 g (92.2%) of **9** as a white solid; mp: 111–113 °C. ^1^H NMR (400 MHz, CDCl_3_) *δ*: 7.59 (d, 1H, *J* 7.6 Hz, Ar-H), 7.32 (d, 1H, *J* 8.4 Hz, Ar-H), 7.21 (dd ∼ t, 1H, *J* 7.6 Hz, Ar-H), 7.10 (dd ∼ t, 1H, *J* 7.4 Hz, Ar-H), 6.98 (s, 1H, Ind-2-H), 5.02 (br-d, 1H, *J* 7.2 Hz, NHBoc), 4.7–4.6 (br-m, 1H, CH), 4.12 (q, 2H, *J* 6.8 Hz, N-CH_2_CH_3_), 3.36 (dd, 1H, *J* 14.4, 5.2 Hz, 10-CH_A_), 3.29 (dd, 1H, *J* 14.8, 5.2 Hz, 10-CH_B_), 1.43 (s, 9H, *t*-Bu), 1.26 (br, 3H, N-CH_2_CH_3_). ^13^C NMR (75 MHz, CDCl_3_, assignments made using DEPT-135) *δ*: 176.6 (C, C12), 155.9 (C, C13), 136.1 (C, Ar-C), 128.5 (C, Ar-C), 126.1 (CH, Ar-C), 121.8 (CH, Ar-C), 119.3 (CH, Ar-C), 119.1 (CH, Ar-C), 109.5 (CH, Ar-C), 108.6 (C, Ar-C), 80.4 (C, C14), 54.4 (CH, C11), 41.0 (CH_2_, N-CH_2_CH_3_), 28.4 (3 × CH_3_, C15, C16, C17), 27.7 (CH_2_, C10), 15.5 (CH_3_, N-CH_2_CH_3_). MS (ESI) *m/z* [M+K]^+^ 371.1. Accurate mass calculated for C_18_H_24_N_2_O_4_K: 371.1368. Found: MS (ESI) 371.1387.

#### 4.1.8 Boc-D-Trp(*N*-propyl)-OH **(10)**

The method of synthesis was similar to that described for **8**; 1 M LiOH was added to 0.7 g of **5** to give 0.56 g (89.7%) of **10** as a white solid; mp: 129–131 °C. ^1^H NMR (400 MHz, CDCl_3_) *δ*: 7.59 (d, 1H, *J* 7.6 Hz, Ar-H), 7.31 (d, 1H, *J* 8.0 Hz, Ar-H), 7.20 (dd ∼ t, 1H, *J* 7.4 Hz, Ar-H), 7.10 (dd ∼ t, 1H, *J* 7.4 Hz, Ar-H), 6.96 (s, 1H, Ind-2-H), 5.02 (br-d, 1H, *J* 7.6 Hz, NHBoc), 4.7–4.6 (br-m, 1H, CH), 4.03 (t, 2H, *J* 6.8 Hz, N-CH_2_CH_2_CH_3_), 3.36 (dd, 1H, *J* 14.6, 5.0 Hz, 10-CH_A_), 3.28 (dd, 1H, *J* 14.8, 5.6 Hz, 10-CH_B_), 1.82 (sextet, 2H, *J* 7.1 Hz, N-CH_2_CH_2_CH_3_), 1.43 (s, 9H, *t*-Bu), 0.89 (t, 3H, *J* 7.4 Hz, N-CH_2_CH_2_CH_3_). ^13^C NMR (75 MHz, CDCl_3_, assignments made using DEPT-135) *δ*: 176.9 (C, C12), 155.8 (C, C13), 136.4 (C, Ar-C), 128.4 (C, Ar-C), 127.0 (CH, Ar-C), 121.8 (CH, Ar-C), 119.3 (CH, Ar-C), 119.0 (CH, Ar-C), 109.6 (CH, Ar-C), 108.4 (C, Ar-C), 80.3 (C, C14), 54.4 (CH, C11), 48.1 (CH_2_, N-CH_2_CH_2_CH_3_), 28.4 (3 × CH_3_, C15, C16, C17), 27.7 (CH_2_, C10), 23.6 (CH_2_, N-CH_2_CH_2_CH_3_), 11.6 (CH_3_, N-CH_2_CH_2_CH_3_). MS (ESI) *m/z* [M+H]^+^ 347.2. Accurate mass calculated for C_19_H_26_N_2_O_4_H: 347.1965. Found: MS (ESI) 347.1979.

#### 4.1.9 Boc-D-Trp(*N*-butyl)-OH **(11)**

The method of synthesis was similar to that described for **8**; 1 M LiOH was added to 0.95 g of **6** to give 0.73 g (88.8%) of **11** as a white solid; mp: 147–149 °C. ^1^H NMR (400 MHz, CDCl_3_) *δ*: 7.59 (d, 1H, *J* 7.6 Hz, Ar-H), 7.31 (d, 1H, *J* 8.4 Hz, Ar-H), 7.20 (dd ∼ t, 1H, *J* 7.4 Hz, Ar-H), 7.10 (dd ∼ t, 1H, *J* 7.4 Hz, Ar-H), 6.96 (s, 1H, Ind-2-H), 5.02 (br-d, 1H, *J* 7.6 Hz, NHBoc), 4.7–4.6 (br-m, 1H, CH), 4.06 (t, 2H, *J* 6.6 Hz, N-CH_2_CH_2_CH_2_CH_3_), 3.36 (dd, 1H, *J* 14.8, 5.2 Hz, 10-CH_A_), 3.28 (dd, 1H, *J* 14.8, 5.6 Hz, 10-CH_B_), 1.78 (pentet, 2H, *J* 7.3 Hz, N-CH_2_CH_2_CH_2_CH_3_), 1.43 (s, 9H, *t*-Bu), 1.35–1.23 (m, 2H, N-CH_2_CH_2_CH_2_CH_3_), 0.92 (t, 3H, *J* 7.4 Hz, N-CH_2_CH_2_CH_2_CH_3_). ^13^C NMR (75 MHz, CDCl_3_, assignments made using DEPT-135) *δ*: 176.9 (C, C12), 155.8 (C, C13), 136.4 (C, Ar-C), 128.4 (C, Ar-C), 126.9 (CH, Ar-C), 121.8 (CH, Ar-C), 119.2 (CH, Ar-C), 119.0 (CH, Ar-C), 109.6 (CH, Ar-C), 108.4 (C, Ar-C), 80.3 (C, C14), 54.4 (CH, C11), 46.1 (CH_2_, N-CH_2_CH_2_CH_2_CH_3_), 32.4 (CH_2_, N-CH_2_CH_2_CH_2_CH_3_), 28.4 (3 × CH_3_, C15, C16, C17), 27.7 (CH_2_, C10), 20.3 (CH_2_, N-CH_2_CH_2_CH_2_CH_3_), 13.8 (CH_3_, N-CH_2_CH_2_CH_2_CH_3_). MS (ESI) *m/z* [M − H]^-^ 359.2. Accurate mass calculated for C_20_H_28_N_2_O_4_-H: 359.1976. Found: MS (ESI) 359.1965.

#### 4.1.10 Boc-D-Trp(*N*-pentyl)-OH **(12)**

The method of synthesis was similar to that described for **8**; 1 M LiOH was added to 0.45 g of **7** to give 0.31 g (81.8%) of **12** as a white solid; mp: 99–101 °C. ^1^H NMR (400 MHz, CDCl_3_) *δ*: 7.59 (d, 1H, *J* 7.6 Hz, Ar-H), 7.31 (d, 1H, *J* 8.0 Hz, Ar-H), 7.20 (dd ∼ t, 1H, *J* 7.4 Hz, Ar-H), 7.09 (dd ∼ t, 1H, *J* 7.4 Hz, Ar-H), 6.96 (s, 1H, Ind-2-H), 5.01 (br-d, 1H, *J* 7.6 Hz, NHBoc), 4.7–4.6 (br-m, 1H, CH), 4.06 (t, 2H, *J* 7.0 Hz, N-CH_2_CH_2_CH_2_CH_2_CH_3_), 3.35 (dd, 1H, *J* 14.8, 5.2 Hz, 10-CH_A_), 3.28 (dd, 1H, *J* 14.8, 5.6 Hz, 10-CH_B_), 1.79 (pentet, 2H, *J* 7.2 Hz, N-CH_2_CH_2_CH_2_CH_2_CH_3_), 1.42 (s, 9H, *t*-Bu), 1.36–1.22 (m, 4H, N-CH_2_CH_2_CH_2_CH_2_CH_3_), 0.87 (t, 3H, *J* 7.0 Hz, N-CH_2_CH_2_CH_2_CH_2_CH_3_). ^13^C NMR (75 MHz, CDCl_3_, assignments made using DEPT-135) *δ*: 176.4 (C, C12), 155.8 (C, C13), 136.4 (C, Ar-C), 128.4 (C, Ar-C), 126.9 (CH, Ar-C), 121.8 (CH, Ar-C), 119.3 (CH, Ar-C), 119.0 (CH, Ar-C), 109.6 (CH, Ar-C), 108.4 (C, Ar-C), 80.4 (C, C14), 54.4 (CH, C11), 46.5 (CH_2_, N-CH_2_CH_2_CH_2_CH_2_CH_3_), 30.0 (CH_2_, N-CH_2_CH_2_CH_2_CH_2_CH_3_), 29.3 (CH_2_, N-CH_2_CH_2_CH_2_CH_2_CH_3_), 28.4 (3 × CH_3_, C15, C16, C17), 27.7 (CH_2_, C10), 22.4 (CH_2_, N-CH_2_CH_2_CH_2_CH_2_CH_3_), 14.1 (CH_3_, N-CH_2_CH_2_CH_2_CH_2_CH_3_). MS (ESI) *m/z* [M+H]^+^ 375.2. Accurate mass calculated for C_21_H_30_N_2_O_4_H: 375.2278. Found: MS (APCI) 375.2277.

### 4.2 Materials and instrumentation for synthesis of peptides

Chemicals and solvents were obtained from Alfa Aesar, AGTC Bioproducts, Sigma-Aldrich and Fisher Scientific. The synthesised N^ind^-modified Boc-D-Trp-OH derivatives were used in the peptides synthesis. RP-HPLC was performed using PerkinElmer Series 200 instruments. The peptides were purified by RP-HPLC using C4 column (ACE 10C4 – 250 × 21.2 mm id) at *λ*_*max*_ 220 nm using solution A (0.1% v/v trifluoroacetic acid (TFA)/H_2_O) and solution B (0.1% v/v TFA/acetonitrile (MeCN)) with a flow rate of 8 ml/min. The peptides were isolated as their TFA salt, with protonation of the terminal NMe groups. Peptides *t*_*R*_ reported are from the quality control assessment of the purity of the peptides performed using C8 column (ACE 5C8 – 250 × 4.6 mm id) at *λ*_*max*_ 220 nm with same solutions (A and B) and flow rate of 1 ml/min; chromatograms were generated using TotalChrom 6.3.1 software. ESIMS, APCIMS and ^1^H NMR spectroscopy were performed using the instruments stated in Section 4.1. ^1^H NMR spectroscopy was performed for peptides **16** and **18**–**20** and their corresponding ^1^H NMR spectra are presented in SD (Fig. S20a-S23b).

#### 4.2.1 General procedure for the synthesis of peptides

The first amino acid in the peptide sequence or the short chain peptide was dissolved in THF and cooled under ice. Molar equivalents of NHS and DCC were dissolved in THF and added to the amino acid or short chain peptide. The mixture was left overnight cooled under ice to RT. The by-product dicyclohexylurea (DCU) precipitated out in the mixture, and could be removed by filtration. The filtrate was cooled under ice. The next amino acid in the peptide sequence (2 molar equivalents) and 8 molar equivalents of Na_2_CO_3_ were dissolved in minimal amount of water and added to the filtrate. The mixture was left overnight at RT. Then the mixture was acidified with excess 1 M HCl and the precipitate formed was extracted with ethyl acetate. The ethyl acetate layer was evaporated and the solid remaining was either dissolved in THF, if further amino acid in the peptide sequence need to be coupled, or lyophilised to remove any remaining solvent. The amino acids being coupled in the LPPS have their amine group unprotected, except for the first amino acid (N-Me-D-Phe-OH) and the N^ind^-modified D-Trp-OH derivatives in which they have Boc protection on the amino group. Before coupling the N^ind^-modified amino acid, the Boc group was removed with 50% v/v TFA/dichloromethane (DCM) for 1 h and then solvents evaporated. When the coupling of the amino acids ends and the peptide is lyophilised, it is still Boc protected. The Boc protected peptide was dissolved in 50% v/v TFA/DCM for 1 h to remove the Boc group and then the solvents were evaporated to give the crude peptide, which was purified by preparative RP-HPLC and characterised MS and/or ^1^H NMR.

#### 4.2.2 Methyl^4th^-NH_2_**(13)**

The crude product was purified using preparative RP-HPLC with the following linear gradient elution: 0–35% solution B from 0 to 5 mins, 35–70% solution B from 5 to 45 mins, and 70–100% solution B from 45 to 50 mins, along with solution A. The peptide (**13**) was collected and lyophilised. RP-HPLC *t*_*R*_ 22.51 min. MS (APCI) *m/z* [M+H]^+^ 825.3. Accurate mass calculated for C_48_H_56_N_8_O_5_H: 825.4446. Found: MS (APCI) 825.4458.

#### 4.2.3 Ethyl^4th^-NH_2_**(14)**

The crude product was purified as in **13**, to give **14**. RP-HPLC *t*_*R*_ 23.16 min. MS (APCI) *m/z* [M+H]^+^ 839.7. Accurate mass calculated for C_49_H_58_N_8_O_5_H: 839.4603. Found: MS (APCI) 839.4602.

#### 4.2.4 Propyl^4th^-NH_2_**(15)**

The crude product was purified as in **13**, to give **15**. RP-HPLC *t*_*R*_ 23.95 min. MS (APCI) *m/z* [M+H]^+^ 853.9. Accurate mass calculated for C_50_H_60_N_8_O_5_H: 853.4759. Found: MS (APCI) 853.4755.

#### 4.2.5 Butyl^4th^-NH_2_**(16)**

The crude product was purified as in **13**, to give **16**. RP-HPLC *t*_*R*_ 24.74 min ^1^H NMR (400 MHz, DMSO-*d*_6_) *δ*: 10.71 (s, 1H, Ind-1′-NH), 8.62 (d, 2H, *J* 9.2 Hz, NH_2_-amide), 8.55 (d, 1H, *J* 8.0 Hz, NH-amide), 8.44 (d, 2H, *J* 8.8 Hz, NH-amide), 8.34 (d, 1H, *J* 8.4 Hz, NH-amide), 7.74 (d, 1H, *J* 8.0 Hz, Ar-H), 7.65 (d, 1H, *J* 7.6 Hz, Ar-H), 7.38 (d, 1H, *J* 8.0 Hz, Ar-H), 7.29–7.25 (m, 2H, Ar-H), 7.24 (s, 1H, Ind-2′-H), 7.21–6.90 (m, 14H, Ar-H), 4.78–4.67 (m, 3H, CαH), 4.28–4.22 (m, 1H, CαH), 4.04 (t, 2H, *J* 7.0, N-CH_2_CH_2_CH_2_CH_3_), 3.77 (br-d, 1H, *J* 6.4 Hz, CαH), 3.11 (dd, 1H, *J* 14.2, 5.4 Hz, CβH_2_), 2.96–2.81 (m, 3H, CβH_2_), 2.67–2.64 (m, 2H, CβH_2_), 2.45–2.39 (m, 2H, CβH_2_), 1.75 (t, 3H, *J* 5.2 Hz, ^+^NH_2_CH_3_), 1.64 (pentet, 2H, *J* 7.4 Hz, N-CH_2_CH_2_CH_2_CH_3_), 1.41–1.34 (m, 3H, 3″-CH and 4″-CH_2_), 1.19 (sextet, 2H, *J* 7.4 Hz, N-CH_2_CH_2_CH_2_CH_3_), 0.80–0.71 (m, 9H, 6H of 1″-CH_3_ and 2″-CH_3_ and 3H of N-CH_2_CH_2_CH_2_CH_3_). MS (APCI) *m/z* [M+H]^+^ 867.7. Accurate mass calculated for C_51_H_62_N_8_O_5_H: 867.4916. Found: MS (APCI) 867.4927.

#### 4.2.6 Pentyl^4th^-NH_2_**(17)**

The crude product was purified using preparative RP-HPLC with the following linear gradient elution: 0–50% solution B from 0 to 5 mins, 50–85% solution B from 5 to 45 mins, and 85–100% solution B from 45 to 50 mins, along with solution A. The peptide (**17**) was collected and lyophilised. RP-HPLC *t*_*R*_ 25.61 min. MS (APCI) *m/z* [M+H]^+^ 881.5068. Accurate mass calculated for C_52_H_64_N_8_O_5_H: 881.5072. Found: MS (APCI) 881.5068.

#### 4.2.7 Butyl^2nd^-NH_2_**(18)**

The crude product was purified as in **13**, to give **18**. RP-HPLC *t*_*R*_ 24.17 min ^1^H NMR (400 MHz, DMSO-*d*_6_) *δ*: 10.82 (s, 1H, Ind-1-NH), 8.66 (d, 2H, *J* 8.8 Hz, NH_2_-amide), 8.52 (d, 1H, *J* 7.6 Hz, NH-amide), 8.40 (d, 2H, *J* 8.8 Hz, NH-amide), 8.30 (d, 1H, *J* 8.4 Hz, NH-amide), 7.73 (d, 1H, *J* 7.6 Hz, Ar-H), 7.64 (d, 1H, *J* 7.6 Hz, Ar-H), 7.34–6.89 (m, 18H, Ar-H), 4.79–4.64 (m, 3H, CαH), 4.27–4.21 (m, 1H, CαH), 4.06–3.90 (m, 2H, N-CH_2_CH_2_CH_2_CH_3_), 3.77 (br-d, 1H, *J* 6.8 Hz, CαH), 3.12 (dd, 1H, *J* 14.4, 5.6 Hz, CβH_2_), 2.97–2.81 (m, 3H, CβH_2_), 2.71–2.63 (m, 2H, CβH_2_), 2.46–2.28 (m, 2H, CβH_2_), 1.77 (t, 3H, *J* 5.0 Hz, ^+^NH_2_CH_3_), 1.62 (pentet, 2H, *J* 7.3 Hz, N-CH_2_CH_2_CH_2_CH_3_), 1.38–1.33 (m, 3H, 3″-CH and 4″-CH_2_), 1.23 (sextet, 2H, *J* 7.4 Hz, N-CH_2_CH_2_CH_2_CH_3_), 0.86 (t, 3H, *J* 7.2 Hz, N-CH_2_CH_2_CH_2_CH_3_), 0.79 (d, 3H, *J* 6.4 Hz, 1″-CH_3_), 0.72 (d, 3H, *J* 6.0 Hz, 2″CH_3_). MS (ESI) *m/z* [M+H]^+^ 867.4836. Accurate mass calculated for C_51_H_62_N_8_O_5_H: 867.4916. Found: MS (ESI) 867.4912.

#### 4.2.8 Butyl^2nd,4th^-NH_2_**(19)**

The crude product was purified using preparative RP-HPLC with the following linear gradient elution: 0–45% solution B from 0 to 5 mins, 45–90% solution B from 5 to 45 mins, and 90–100% solution B from 45 to 50 mins, along with solution A. The peptide (**19**) was collected and lyophilised. RP-HPLC *t*_*R*_ 27.75 min ^1^H NMR (400 MHz, DMSO-*d*_6_) *δ*: 8.64 (d, 2H, *J* 9.2 Hz, NH_2_-amide), 8.54 (d, 2H, *J* 8.0 Hz, NH-amide), 8.41 (d, 1H, *J* 8.8 Hz, NH-amide), 8.33 (d, 1H, *J* 8.8 Hz, NH-amide), 7.74 (d, 1H, *J* 7.6 Hz, Ar-H), 7.64 (d, 1H, *J* 8.0 Hz, Ar-H), 7.37 (d, 1H, *J* 8.4 Hz, Ar-H), 7.33 (d, 1H, *J* 8.4 Hz, Ar-H), 7.29–6.95 (m, 14H, Ar-H), 6.90 (s, 2H, Ind-2-H and Ind-2′-H), 4.78–4.67 (m, 3H, CαH), 4.27–4.22 (m, 1H, CαH), 4.05–3.95 (m, 4H, 1N-CH_2_CH_2_CH_2_CH_3_ and 1′N-CH_2_CH_2_CH_2_CH_3_), 3.76 (br-s, 1H, CαH), 3.11 (dd, 1H, *J* 14.2, 5.4 Hz, CβH_2_), 2.96–2.79 (m, 3H, CβH_2_), 2.70–2.63 (m, 2H, CβH_2_), 2.47–2.38 (m, 2H, CβH_2_), 1.77 (br-s, 3H, ^+^NH_2_CH_3_), 1.67–1.58 (m, 4H, 1N-CH_2_CH_2_CH_2_CH_3_ and 1′N-CH_2_CH_2_CH_2_CH_3_), 1.39–1.34 (m, 3H, 3″-CH and 4″-CH_2_), 1.28–1.16 (m, 4H, 1N-CH_2_CH_2_CH_2_CH_3_ and 1′N-CH_2_CH_2_CH_2_CH_3_), 0.85 (t, 3H, *J* 7.2 Hz, 1′N-CH_2_CH_2_CH_2_CH_3_), 0.79–0.71 (m, 9H, 6H of 1″-CH_3_ and 2″-CH_3_, 3H of 1N-CH_2_CH_2_CH_2_CH_3_). MS (ESI) *m/z* [M+H]^+^ 923.5230. Accurate mass calculated for C_55_H_70_N_8_O_5_H: 923.5542. Found: MS (ESI) 923.5537.

#### 4.2.9 Butyl^4th^-OH **(20)**

The crude product was purified using preparative RP-HPLC with the following linear gradient elution: 0–40% solution B from 0 to 5 mins, 40–75% solution B from 5 to 45 mins, and 75–100% solution B from 45 to 50 mins, along with solution A. The peptide (**20**) was collected and lyophilised. RP-HPLC *t*_*R*_ 25.49 min ^1^H NMR (400 MHz, DMSO-*d*_6_) *δ*: 12.52 (br-s, 1H, -COOH), 10.69 (br-s, 1H, Ind-1′-NH), 8.60–8.52 (m, 3H, NH-amide), 8.44 (d, 1H, *J* 8.8 Hz, NH-amide), 7.77 (d, 1H, *J* 7.6 Hz, Ar-H), 7.65 (d, 1H, *J* 8.0 Hz, Ar-H), 7.37 (d, 1H, *J* 8.0 Hz, Ar-H), 7.26 (d, 1H, *J* 8.0 Hz, Ar-H), 7.23 (s, 1H, Ind-2′-H), 7.18–6.91 (m, 15H, Ar-H), 4.85–4.69 (m, 3H, CαH), 4.31–4.25 (m, 1H, CαH), 4.03 (t, 2H, *J* 7.2 Hz, N-CH_2_CH_2_CH_2_CH_3_), 3.75 (br-d ∼ s, 1H, CαH), 3.11 (dd, 1H, *J* 14.4, 4.8 Hz, CβH_2_), 2.96–2.80 (m, 3H, CβH_2_), 2.67–2.60 (m, 2H, CβH_2_), 2.41–2.32 (m, 2H, CβH_2_), 1.72 (t, 3H, *J* 5.0 Hz, ^+^NH_2_CH_3_, on addition of D_2_O t is converted to s), 1.62 (pentet, 2H, *J* 7.4 Hz, N-CH_2_CH_2_CH_2_CH_3_), 1.49–1.47 (m, 3H, 3″-CH and 4″-CH_2_), 1.20 (sextet, 2H, *J* 7.5 Hz, N-CH_2_CH_2_CH_2_CH_3_), 0.82 (d, 3H, *J* 6 Hz, N-CH_2_CH_2_CH_2_CH_3_), 0.78–0.73 (m, 6H, 1″-CH_3_ and 2″-CH_3_). MS (ESI) *m/z* [M+H]^+^ 868.4495. Accurate mass calculated for C_51_H_61_N_7_O_6_H: 868.4756. Found: MS (ESI) 868.4752.

## 5 Methodologies

### 5.1 Cell subculture and growth conditions

RPMI-1640 (with l-glutamine and NaHCO_3_) was obtained from Sigma-Aldrich. Heat Inactivated Fetal Bovine Serum (FBS) was obtained from Life Technologies. H69 and DMS79 SCLC cell lines were obtained from as previously stated [26]. Cells were grown in 10% v/v FBS/RPMI-1640 (complete medium) and incubated at 37 °C in humidified air atmosphere of 5% CO_2_. Both, H69 and DMS79 cells are suspension cells and grow in aggregates. For the experimental part, cells were re-suspended in fresh complete medium.

### 5.2 Cell viability assays using resazurin dye

Dulbecco's PBS (without calcium chloride and magnesium chloride) and Resazurin sodium salt were obtained from Sigma-Aldrich. The peptide concentrations were calculated accurately using UV/VIS Spectrometer Perkin Elmer instrument and dilution of the stock concentration was made using 50% v/v FBS/sterile saline. Concentrations prepared were ranged from 0.6 to 0.002 mM. Cells were seeded in 96-well plates at a seeding density of 1 × 10^4^ cells/190 μl/well in triplicates. Peptides were then added on the same day and incubated as the above conditions stated in Section 5.1. The amount of peptide solution added into each well was 10 μl, resulting in a final well concentration of 30 to 0.1 μM. Untreated control samples were also prepared. Samples for background fluorescence for the resazurin dye were also prepared using only complete medium. After 48 h incubation, resazurin dye (20 μl; 0.125 mg/ml in PBS) was added to each well and incubated as the above conditions stated in Section 5.1 for 5 h. Then, fluorescence readings were recorded using a Tecan Safire plate reader and % viability calculated. Assays were repeated three independent times. Graphs were generated using GraphPad Prism 7.00 software.

### 5.3 Stability studies

Eppendorf^®^ tubes were used for sample preparations. Chemicals, HPLC microvials, S9 liver fraction (from mouse (CD-1), male, 20 mg/ml protein basis, vial of 1.0 mL) and co-factors: beta-nictoinamide adenine dinucleotide disodium salt hydrate (NADPH), uridine 5’-diphosphoglucuronic acid trisodium salt (UDPGA), l-glutathione (GSH) and adenosine 3’-phosphate 5’-phosphosulfate lithium salt hydrate (PAPS) were obtained from Sigma-Aldrich. RP-HPLC analysis was performed using the instrument, C8 column and solutions (A and B) stated in Section 4.2.

Peptides were added to neat mouse plasma at a concentration of 400 μg/ml and incubated as the above conditions stated in Section 5.1. Aliquots (20 μl) were taken at 0, 3, 24 and 48 h. To the 20 μl sample, 80 μl of solution B was added and left for 5 min, prior to 1 min sonication to precipitate plasma proteins. The sample was then centrifuged for 5 min at full speed. Then 80 μl of the supernatant was added to 120 μl of solution A and transferred into a HPLC microvial to be analysed using the C8 column. A linear gradient elution was made starting with 100% of solution A and ending after 30 mins with 100% of solution B, with a flow rate of 1 ml/min and followed at *λ*_*max*_ 220 nm. The injection volume was 36 μl. Assays were repeated three independent times. HPLC microvials at 0 and 48 h were characterised by ESIMS using the instrument stated in Section 4.1.

The preparation and analysis of the samples for peptides stability in the S9 liver fraction from mouse was similar to that for the mouse plasma studies, except that addition of cofactors for enzymes activation was required and the aliquots were taken at 0, 1, 2 and 3 h. The cofactors were added to initiate the study as previously described [27]. Briefly, stock concentrations of NADPH (40 mM), UDPGA (20 mM), GSH (2 mM) and PAPS (2 mg/ml) were prepared using Tris buffer (200 mM) (pH 7.4) containing 2 mM magnesium chloride. A fresh mixture of the four cofactors was prepared in volume ratio of 1:1:1:1, which was added to the sample containing the peptide and the S9 liver fraction to result in final concentrations for the cofactors: NADPH (1 mM), UDPGA (0.5 mM), GSH (0.05 mM) and PAPS (0.05 mg/ml).

### 5.4 Assessment of apoptosis

#### 5.4.1 Acridine orange/ethidium bromide dual staining

H69 and DMS79 cells were seeded in 96-well plates at a seeding density of 1 × 10^4^ cells/well as in the cell viability assays (Section 5.2). Negative control (untreated) and peptides at a final well concentration of 6 μM were prepared and incubated as the above conditions stated in Section 5.1. After 48 h incubation, 5 μl aliquot of 1:1 AO/EB mix (100 μg/ml in PBS of each dye) were added to each well and viewed under an inverted fluorescence microscope. Images were collected on an Olympus IX83 inverted microscope using a 10×/0.3 UPlanFL N objective lens and captured using an Ocra ER camera (Hamamatsu) through CellSens software (Olympus). Specific band pass filter sets for FITC and Texas Red were used to prevent bleed through from one channel to the next. Images were then processed and analysed using Fiji ImageJ.

#### 5.4.2 Annexin V conjugate and flow cytometric analysis

The experiment was performed according to manufacturer's instructions. Briefly, DMS79 cells were seeded in 12-well plates at a seeding density of 1 × 10^5^ cells/well/1980 μl complete medium. Negative control (untreated), peptides at final well concentrations of 2 and 6 μM were prepared and incubated as the above conditions stated in Section 5.1 for 24 h (total volume of wells was 2 ml). The contents of the wells were then carefully transferred into Eppendorf^®^ tubes and centrifuged for 5 min at 500 rpm and the media discarded. Cells pellets were washed with cold PBS. Cells were gently re-suspended in 100 μl annexin-binding buffer followed by the addition of 5 μl of the Annexin V conjugate (Alexa Fluor^®^ 555) and 0.5 μl of dead cell stain (SYTOX^®^ Blue). Tubes were left for 10 min at RT, then 400 μl of annexin-binding buffer were added and samples were analysed on a flow cytometer (BD Biosciences - LSR Fortessa; Diva 8.0.1 software; Annexin V conjugate was excited with 561 nm laser and fluorescence measured using a 586/15 nm bandpass filter; dead cell stain was excited with 405 nm laser and fluorescence measured using 450/50 nm bandpass filter). Assays were repeated three independent times.

## Funding

This work was supported by the Medical Research Council Confidence in Concept scheme (grant ref. no.: MC_PC_13070).
