# Supplementary material for: Pentapeptides for the treatment of small cell lung cancer: Optimisation by Nind-alkyl modification of the tryptophan side chain
Source: Eur J Med Chem. 2017 Sep 8;137:221–32. doi: 10.1016/j.ejmech.2017.05.053 (PMC5500990; doi:10.1016/j.ejmech.2017.05.053)
Supplement: Online data [file mmc1.docx]

**Supplementary Data**

**Pentapeptides for the Treatment of Small Cell Lung Cancer: Optimisation by N^ind^-alkyl Modification of the Tryptophan Side Chain**

**Osama Haitham Abusara^a^, Sally Freeman^a^ and Harmesh Singh Aojula^a^***

*^a^Division of Pharmacy and Optometry, School of Health Sciences, Faculty of Biology, Medicine and Health, University of Manchester, Manchester M13 9PT, UK*

**Table of contents:**

| ^1^H NMR spectrum for the un-modified Boc-D-Trp-OH and ^1^H/^13^C NMR spectra for the N^ind^-alkylated Boc-D-Trp derivatives | **Figures S1** – **S11c** | **Pages SD2 – SD32** |
| --- | --- | --- |
| RP-HPLC Chromatograms for the pure peptides | **Figures S12** – **S19** | **Pages SD33 – SD36** |
| ^1^H NMR Spectra for the butylated peptides | **Figures S20a** – **S23b** | **Pages SD37 – SD44** |
| Dose-response curves | **Figure S24** | **Pages SD45 – SD46** |
| Quantitative assessment, RP-HPLC Chromatograms and MS Spectra for the stability studies | **Table S1**  **and**  **Figures S25** – **S36** | **Pages SD47 – SD59** |
| Dot blots of flow cytometric analysis | **Figure S37** | **Page SD60** |

*Corresponding author email address: [Harmesh.Aojula@manchester.ac.uk](mailto:Harmesh.Aojula@manchester.ac.uk)


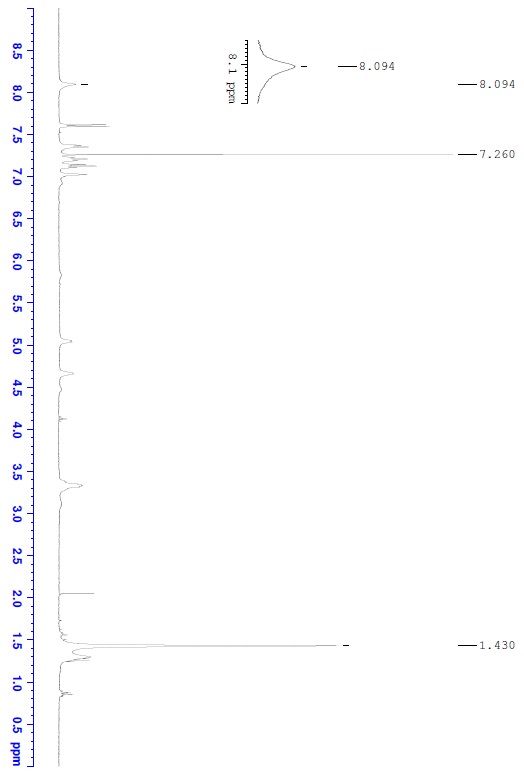

Figure S1. ^1^H NMR (400 MHz, CDCl_3_) spectrum for Boc-D-Trp-OH


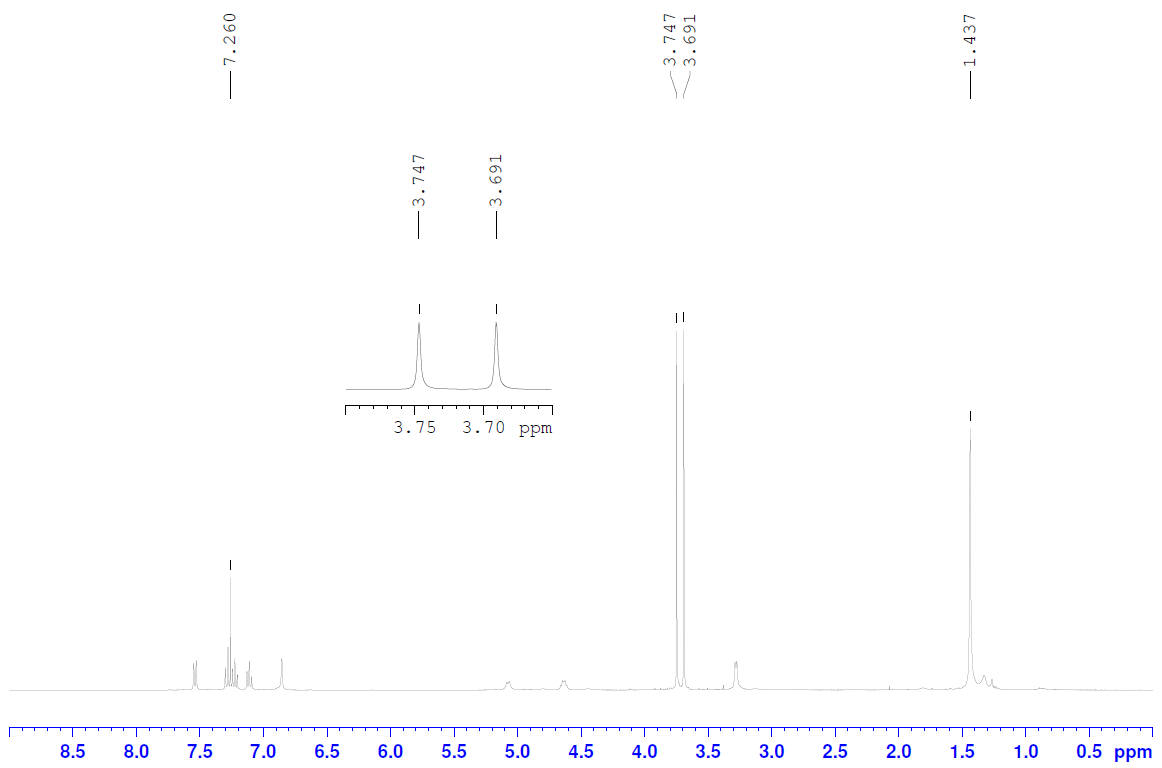

Figure S2a. ^1^H NMR (400 MHz, CDCl_3_) spectra for Boc-D-Trp(N-Methyl)-O-Methyl (3)


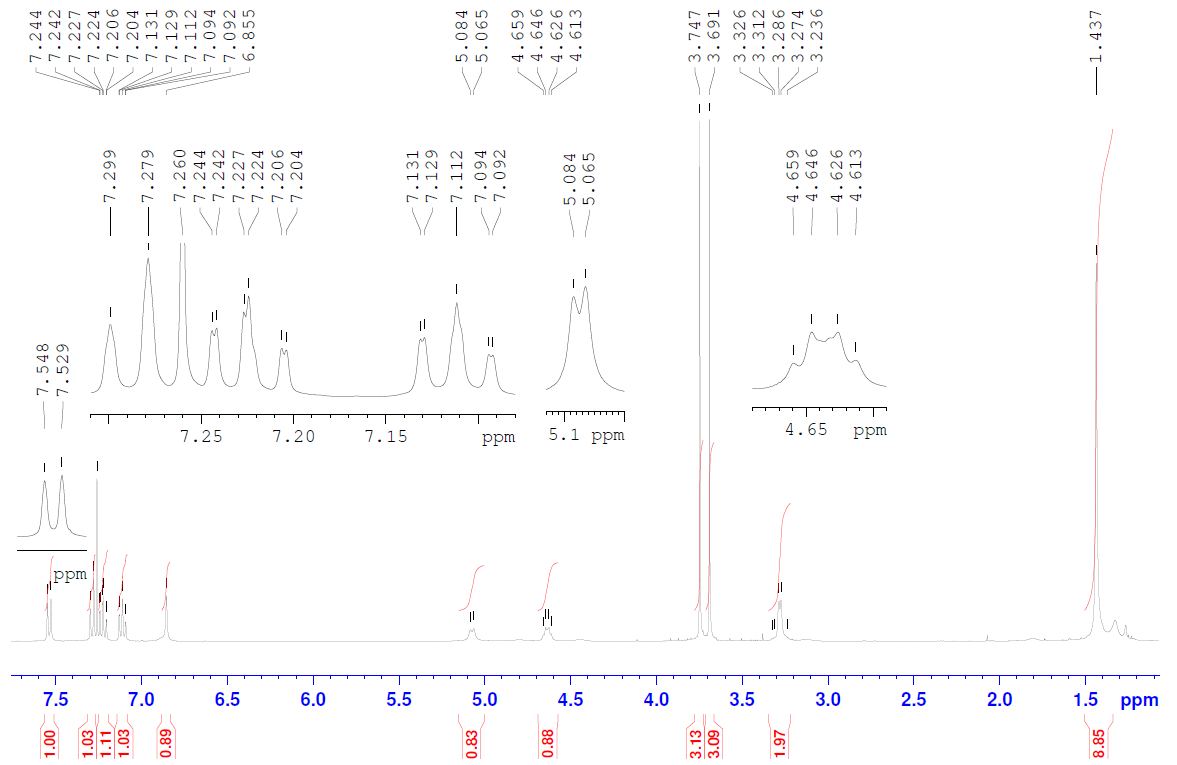


Figure S2b. Expanded ^1^H NMR (400 MHz, CDCl_3_) spectra for Boc-D-Trp(N-Methyl)-O-Methyl (3)


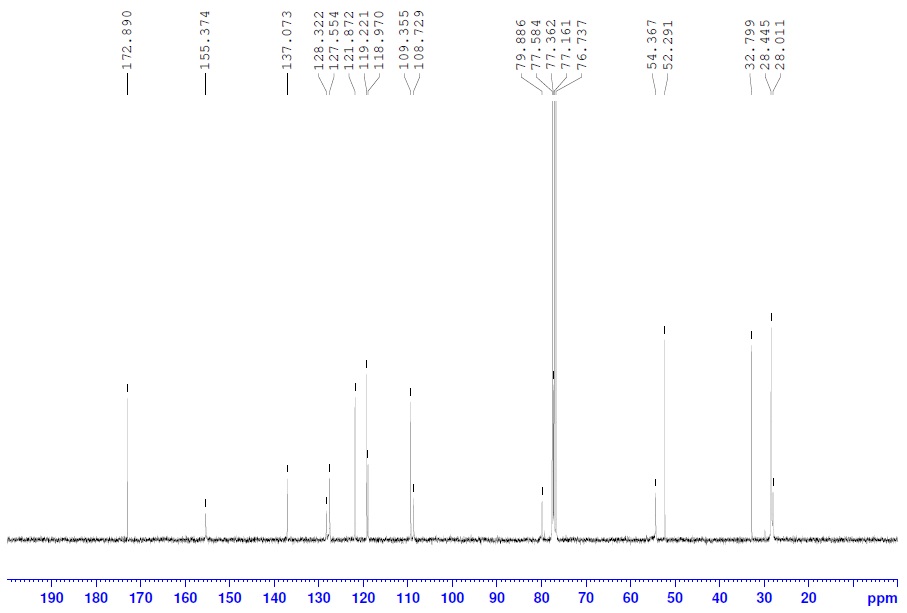

Figure S2c. ^13^C NMR (75 MHz, CDCl_3_) spectra for Boc-D-Trp(N-Methyl)-O-Methyl (3)


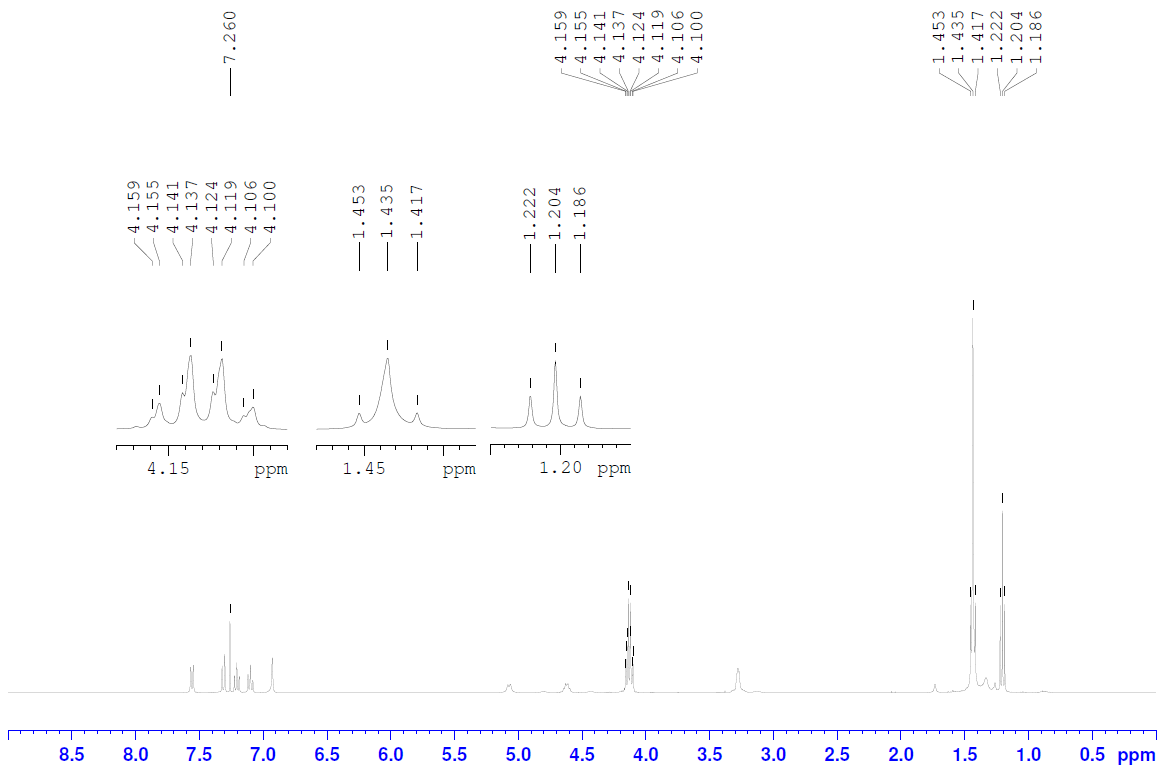

Figure S3a. ^1^H NMR (400 MHz, CDCl_3_) spectra for Boc-D-Trp(N-Ethyl)-O-Ethyl (4)


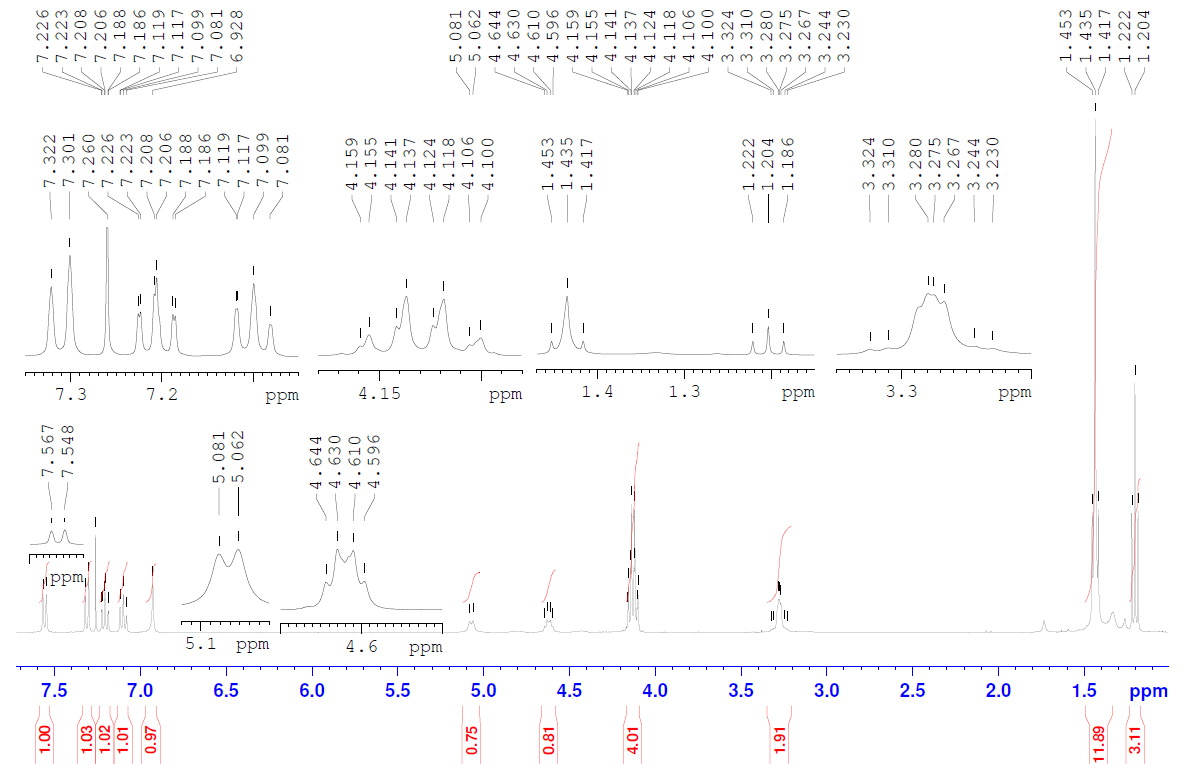


Figure S3b. Expanded ^1^H NMR (400 MHz, CDCl_3_) spectra for Boc-D-Trp(N-Ethyl)-O-Ethyl (4)


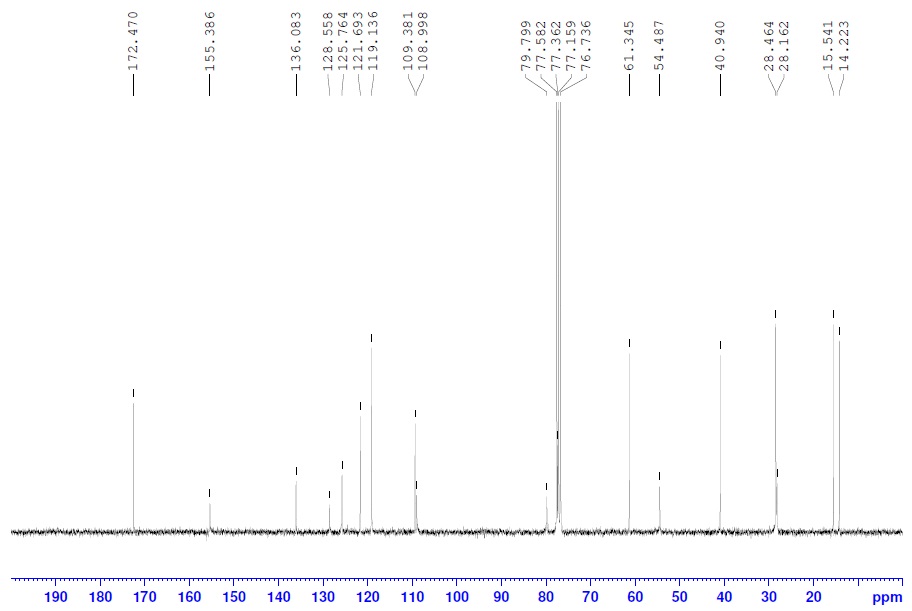

Figure S3c. ^13^C NMR (75 MHz, CDCl_3_) spectra for Boc-D-Trp(N-Ethyl)-O-Ethyl (4)


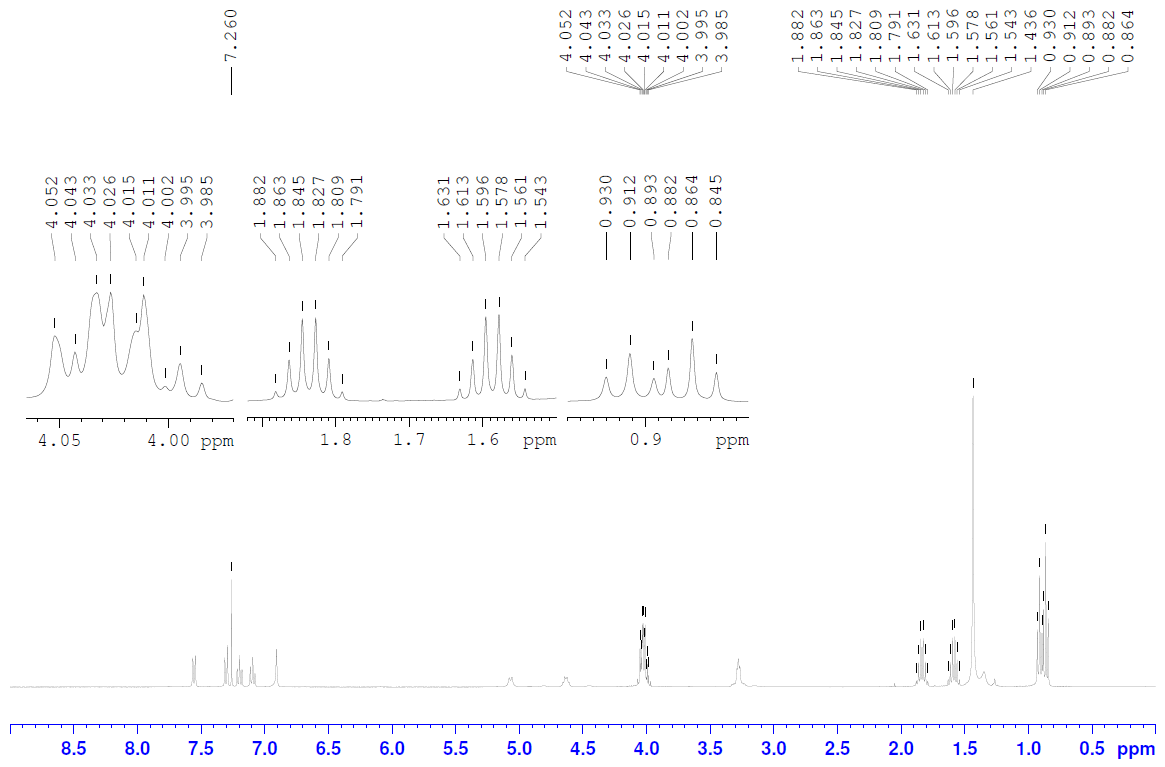

Figure S4a. ^1^H NMR (400 MHz, CDCl_3_) spectra for Boc-D-Trp(N-Propyl)-O-Propyl (5)


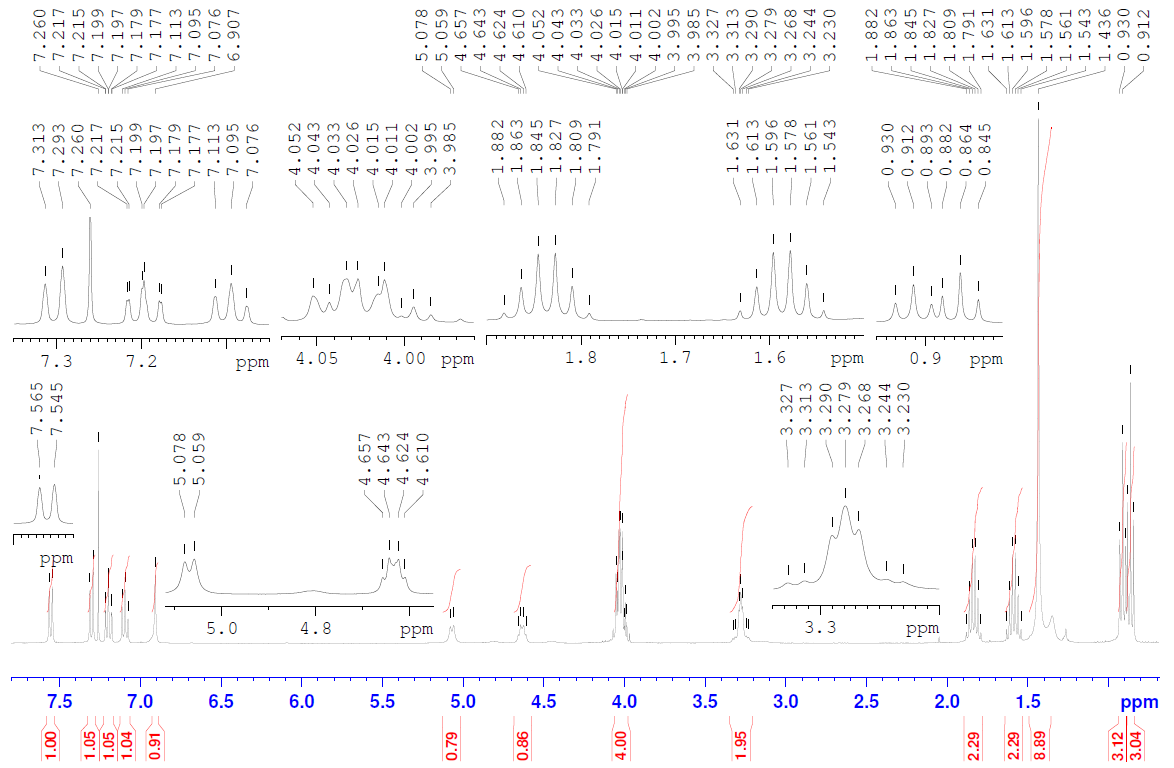


Figure S4b. Expanded ^1^H NMR (400 MHz, CDCl_3_) spectra for Boc-D-Trp(N-Propyl)-O-Propyl (5)


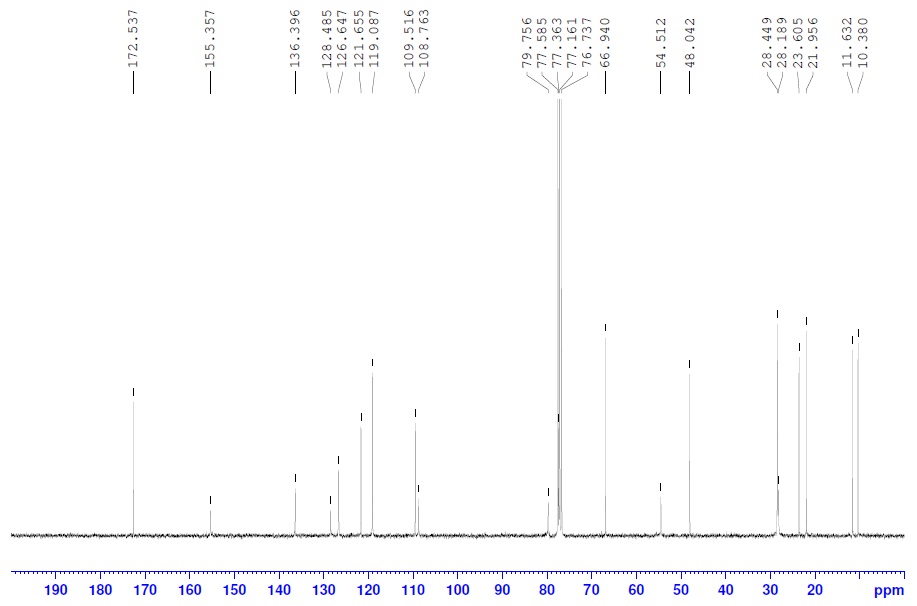

Figure S4c. ^13^C NMR (75 MHz, CDCl_3_) spectra for Boc-D-Trp(N-Propyl)-O-Propyl (5)


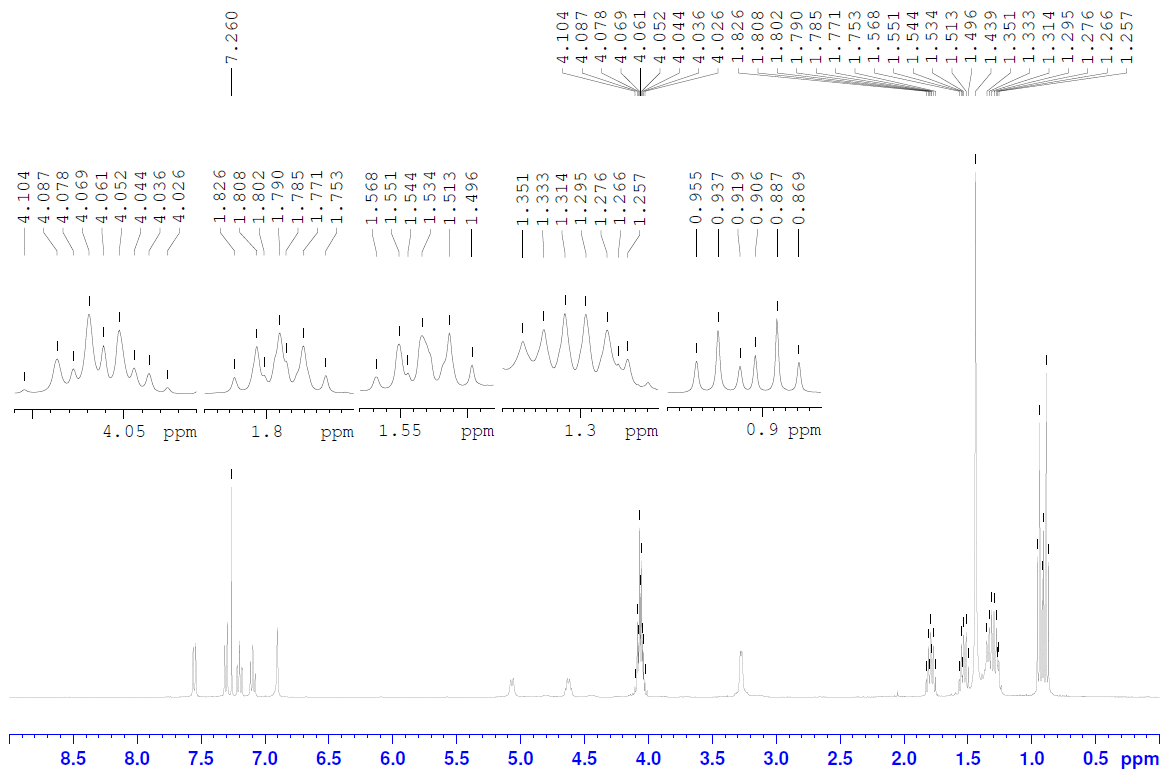

Figure S5a. ^1^H NMR (400 MHz, CDCl_3_) spectra for Boc-D-Trp(N-Butyl)-O-Butyl (6)


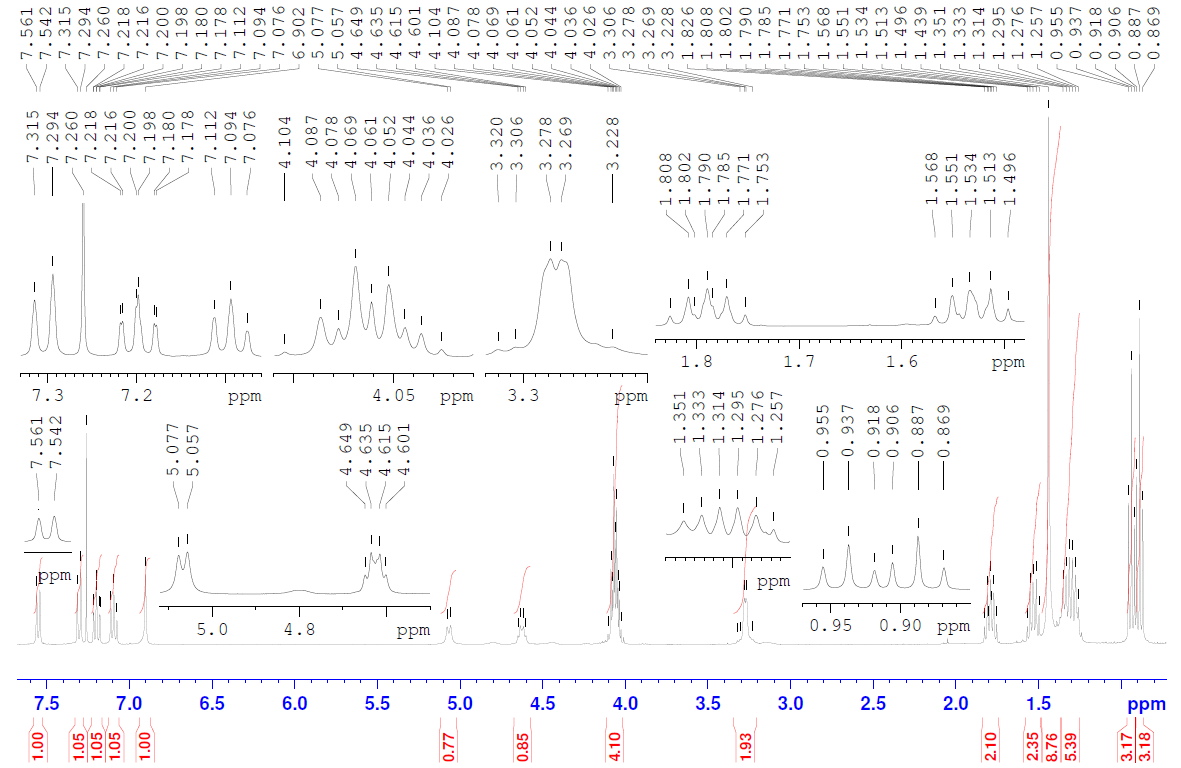


Figure S5b. Expanded ^1^H NMR (400 MHz, CDCl_3_) spectra for Boc-D-Trp(N-Butyl)-O-Butyl (6)


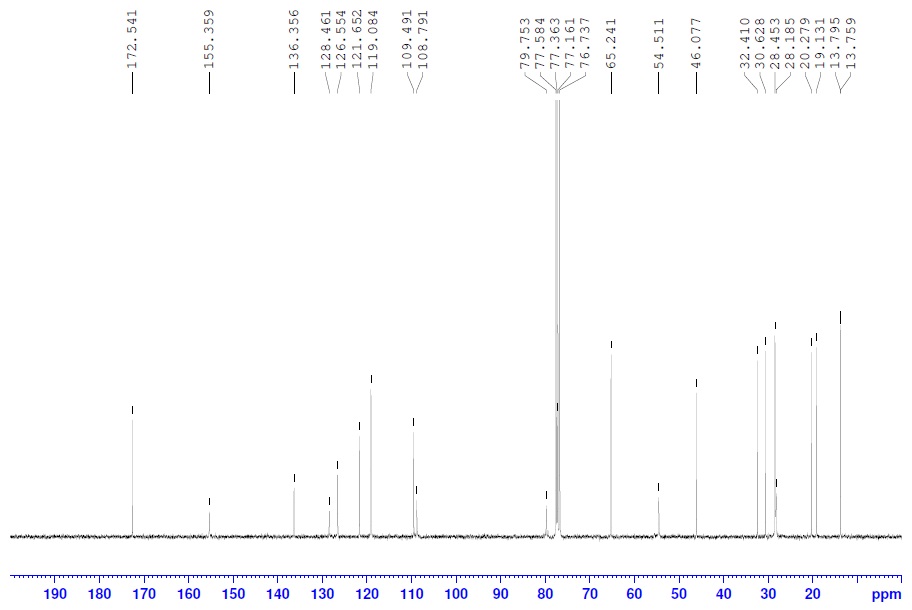

Figure S5c. ^13^C NMR (75 MHz, CDCl_3_) spectra for Boc-D-Trp(N-Butyl)-O-Butyl (6)


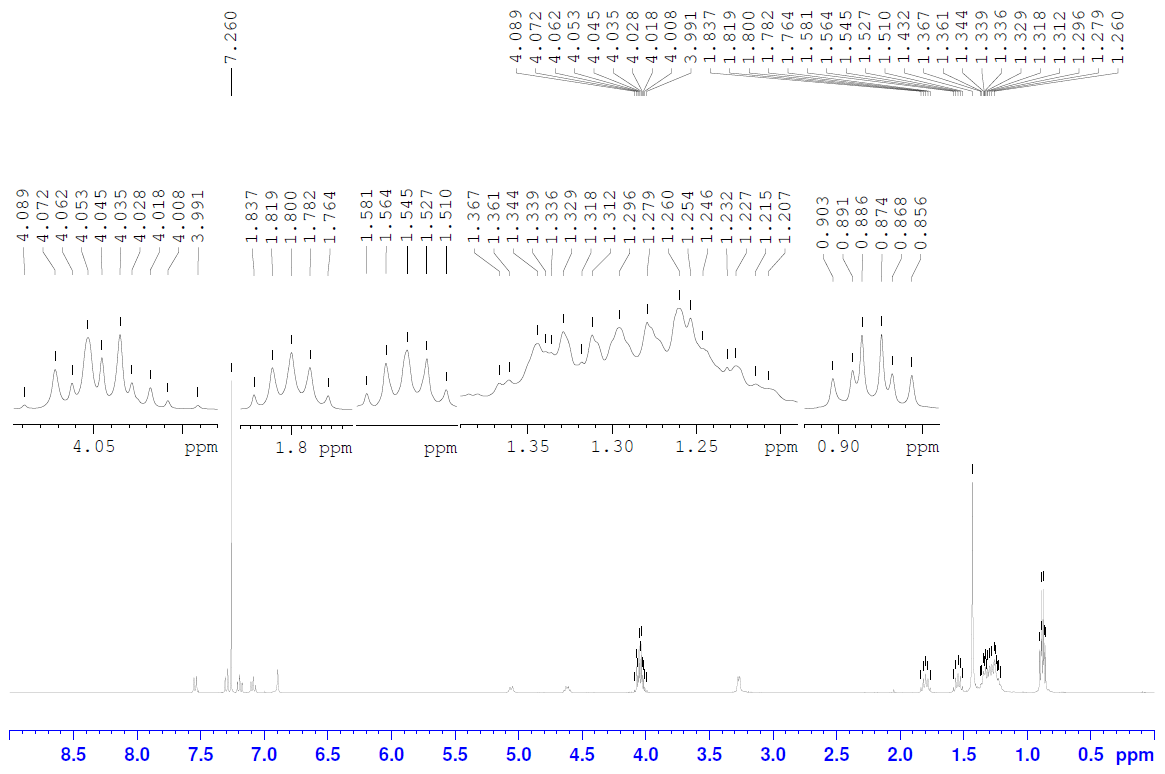

Figure S6a. ^1^H NMR (400 MHz, CDCl_3_) spectra for Boc-D-Trp(N-Pentyl)-O-Pentyl (7)


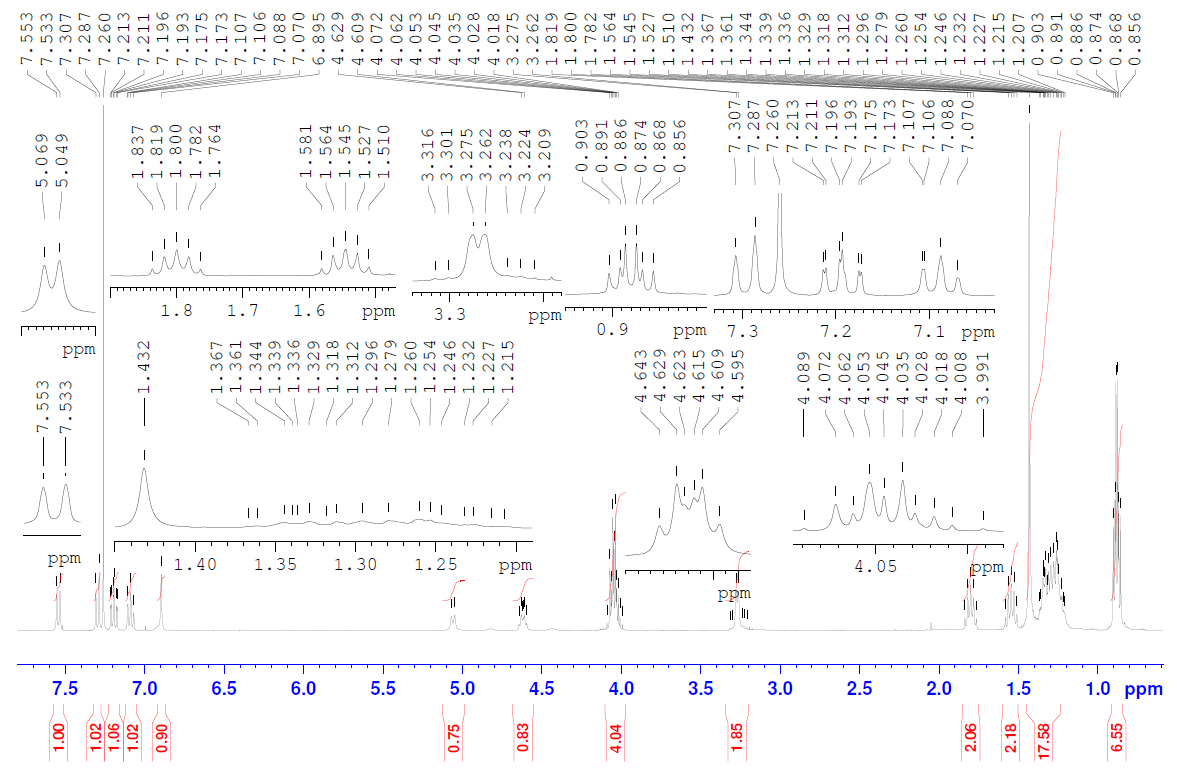


Figure S6b. Expanded ^1^H NMR (400 MHz, CDCl_3_) spectra for Boc-D-Trp(N-Pentyl)-O-Pentyl (7)


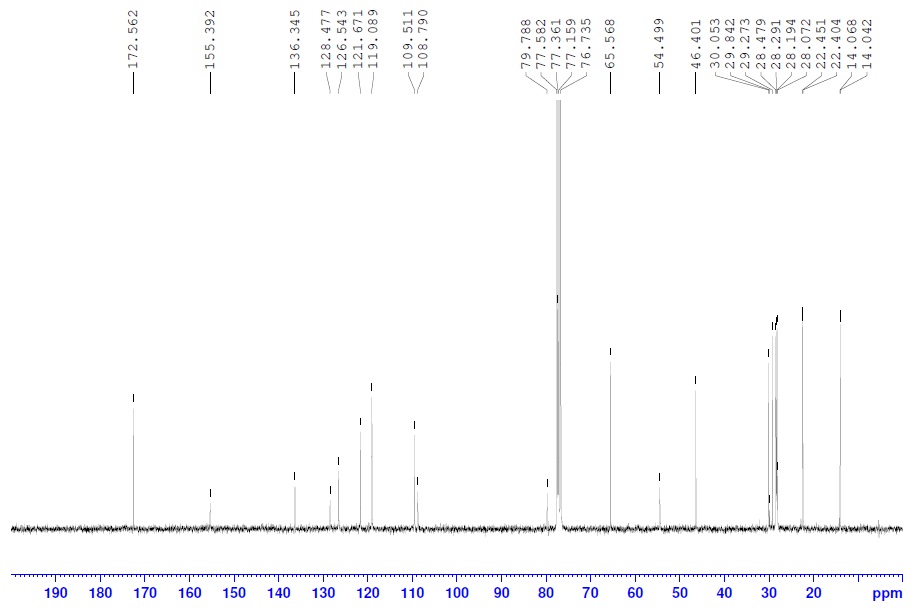

Figure S6c. ^13^C NMR (75 MHz, CDCl_3_) spectra for Boc-D-Trp(N-Pentyl)-O-Pentyl (7)


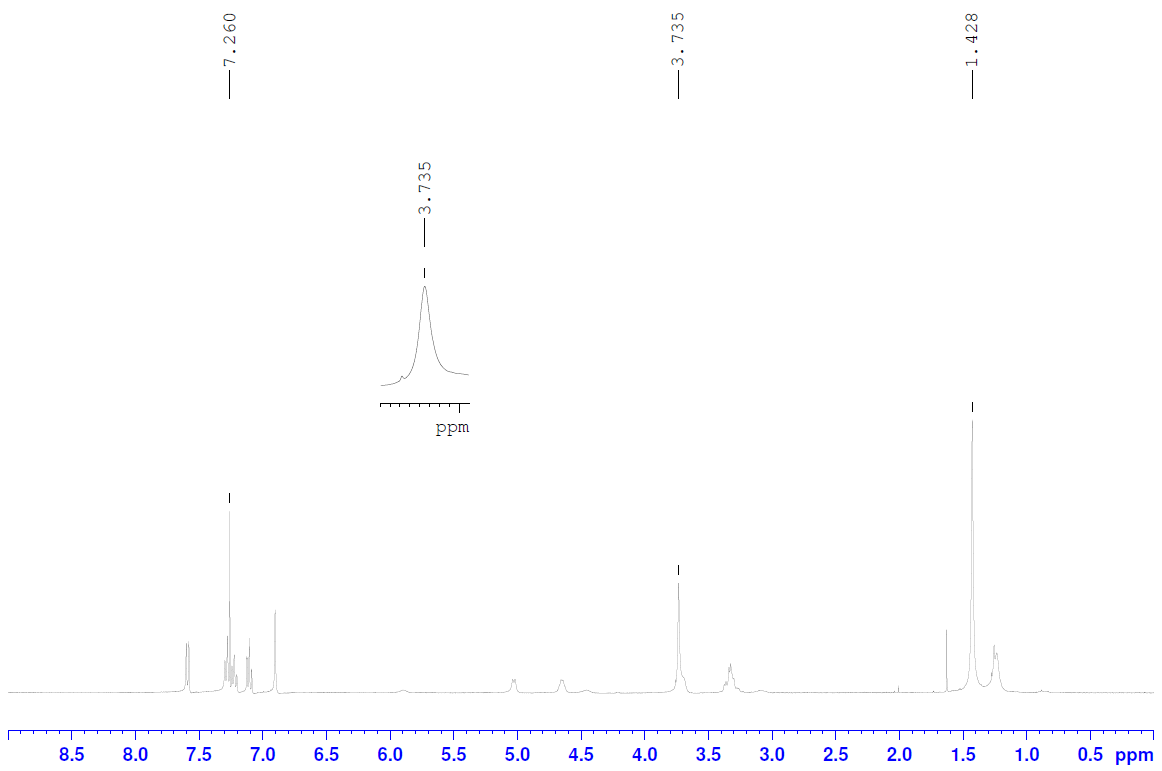

Figure S7a. ^1^H NMR (400 MHz, CDCl_3_) spectra for Boc-D-Trp(N-Methyl)-OH (8)


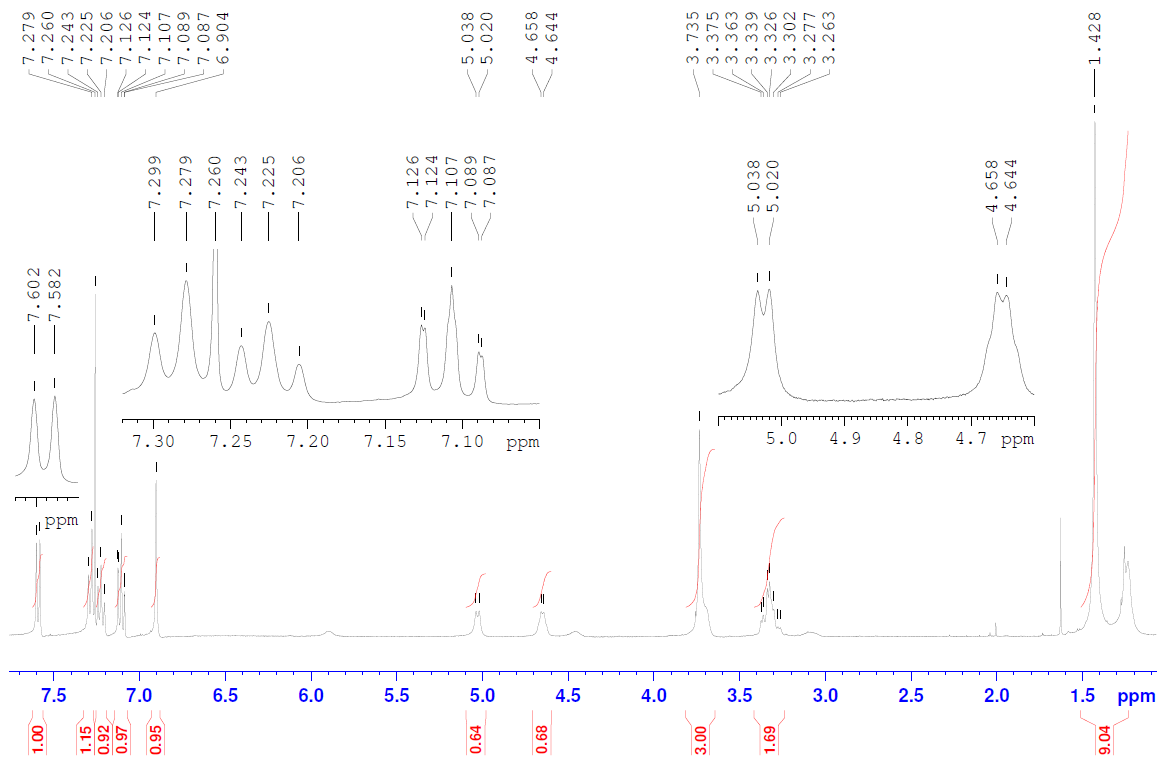


Figure S7b. Expanded ^1^H NMR (400 MHz, CDCl_3_) spectra for Boc-D-Trp(N-Methyl)-OH (8)


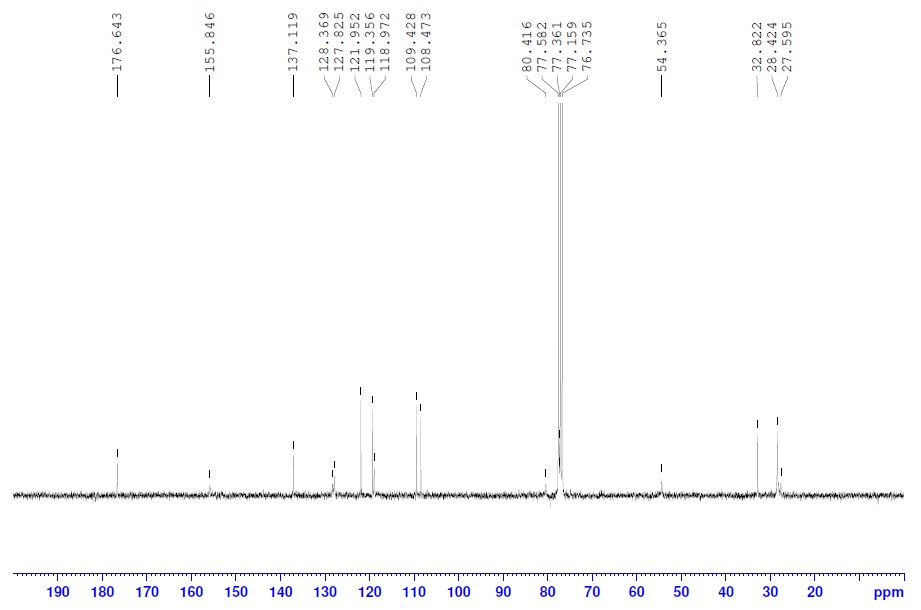

Figure S7c. ^13^C NMR (75 MHz, CDCl_3_) spectra for Boc-D-Trp(N-Methyl)-OH (8)


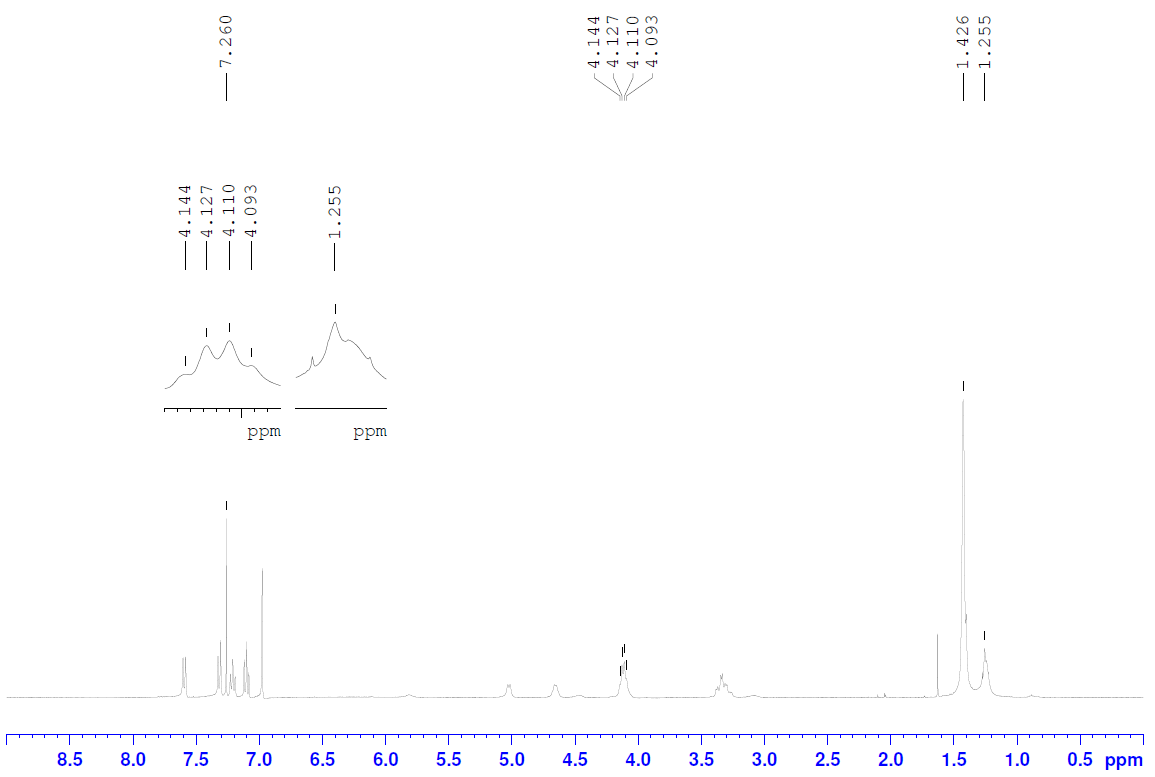

Figure S8a. ^1^H NMR (400 MHz, CDCl_3_) spectra for Boc-D-Trp(N-Ethyl)-OH (9)


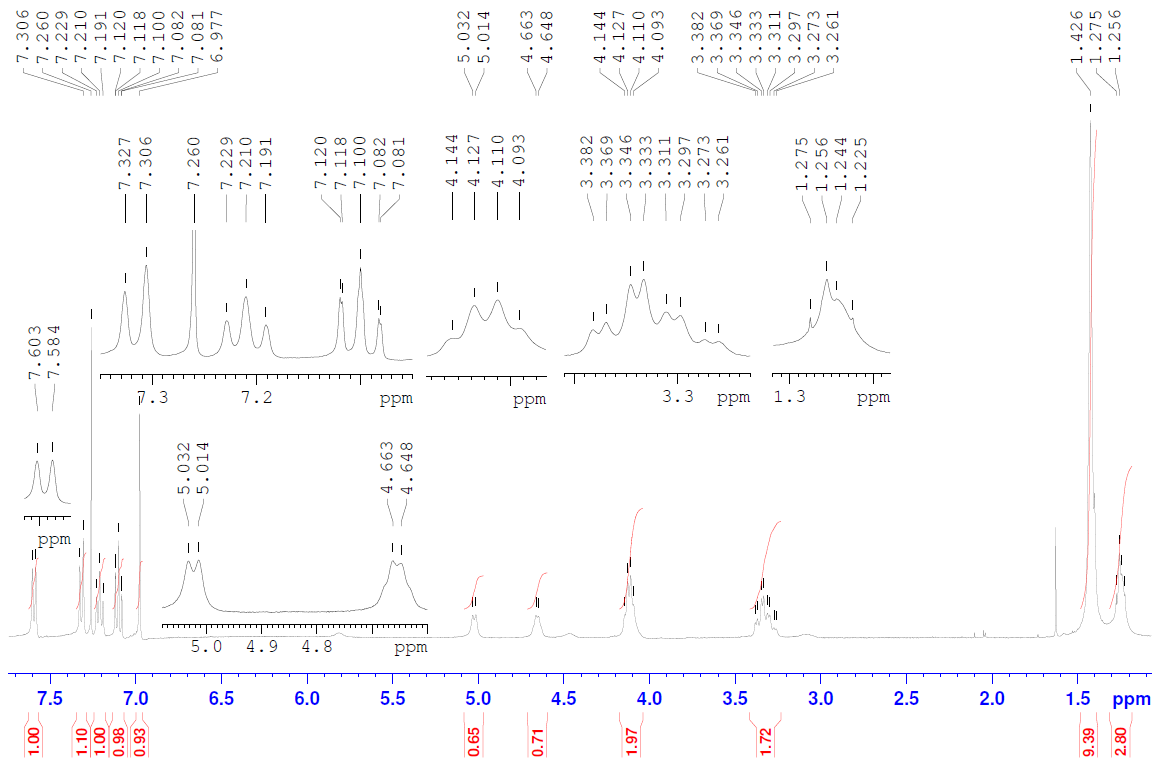


Figure S8b. Expanded ^1^H NMR (400 MHz, CDCl_3_) spectra for Boc-D-Trp(N-Ethyl)-OH (9)


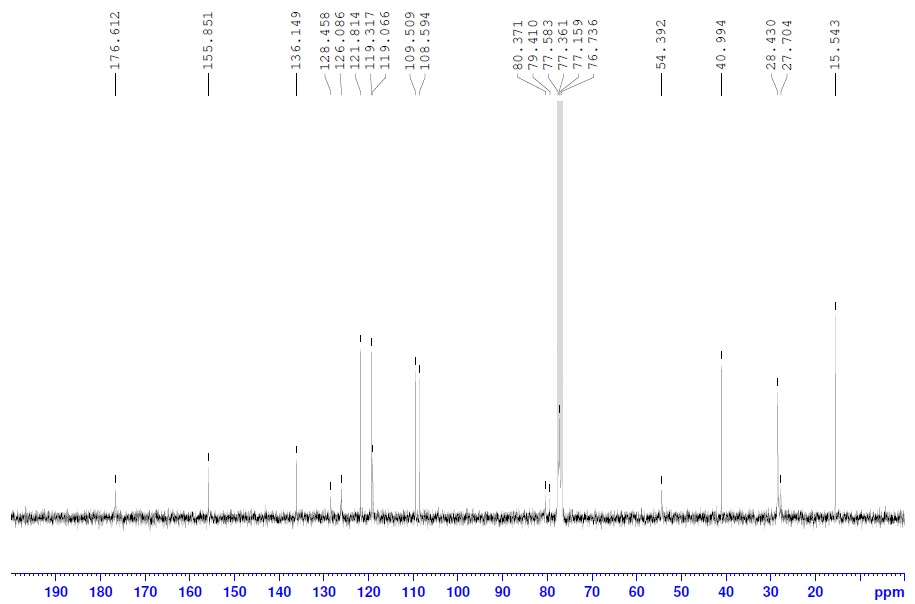

Figure S8c. ^13^C NMR (75 MHz, CDCl_3_) spectra for Boc-D-Trp(N-Ethyl)-OH (9)


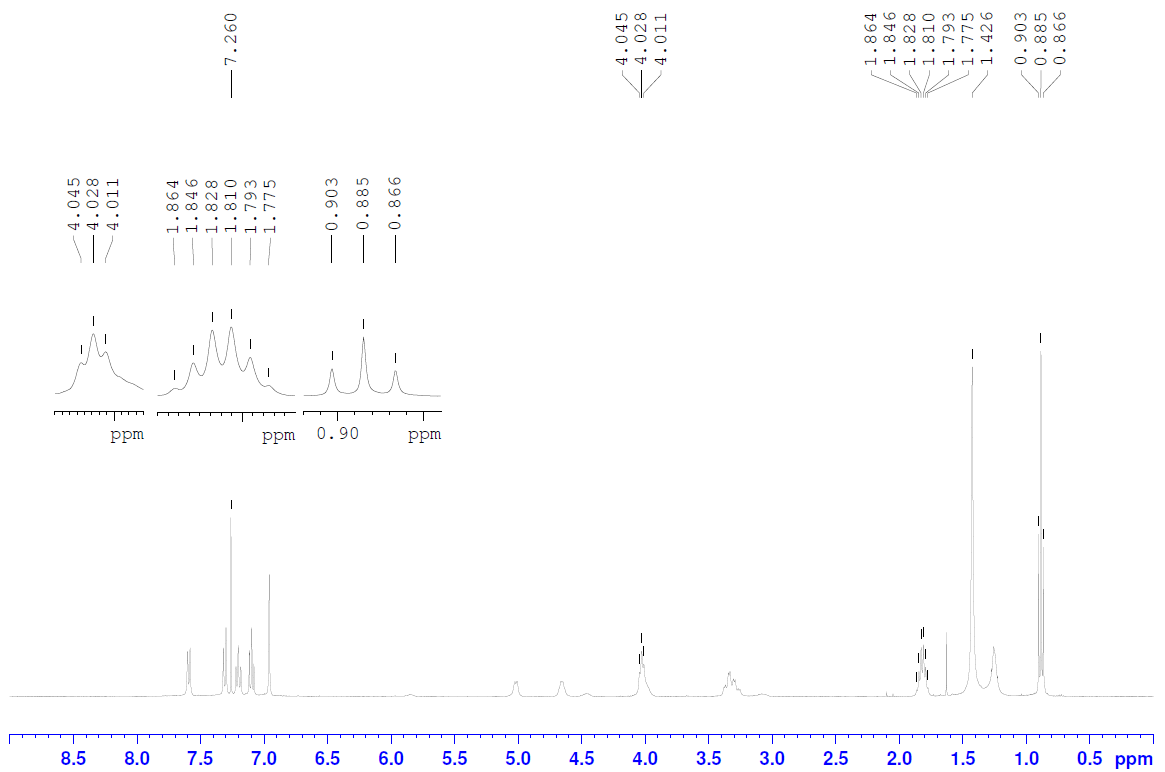

Figure S9a. ^1^H NMR (400 MHz, CDCl_3_) spectra for Boc-D-Trp(N-Propyl)-OH (10)


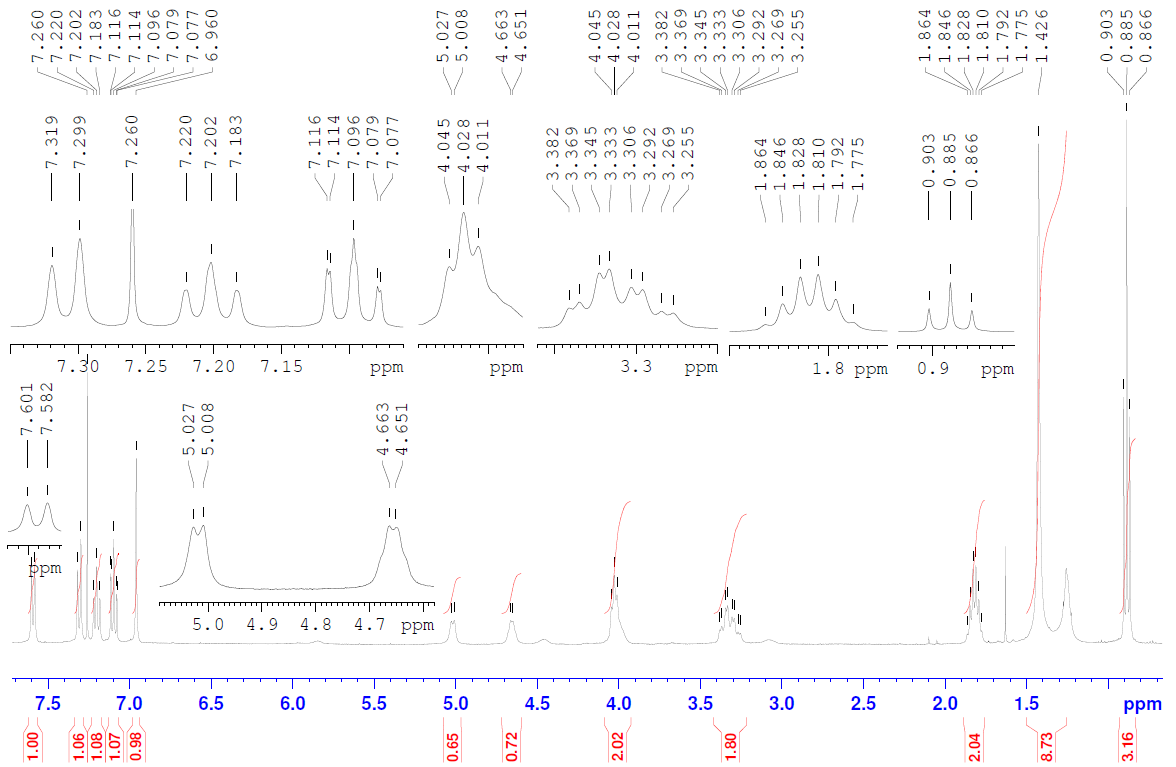


Figure S9b. Expanded ^1^H NMR (400 MHz, CDCl_3_) spectra for Boc-D-Trp(N-Propyl)-OH (10)


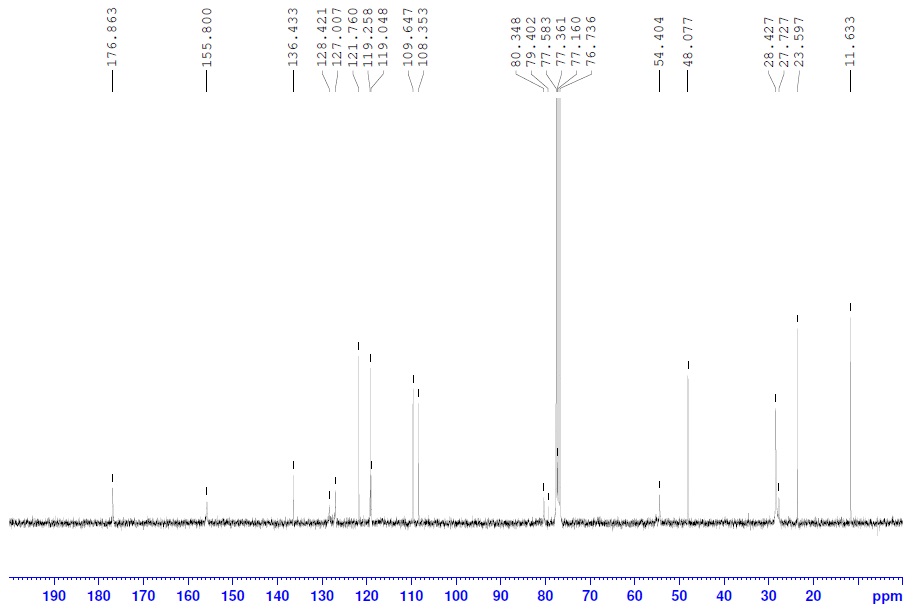

Figure S9c. ^13^C NMR (75 MHz, CDCl_3_) spectra for Boc-D-Trp(N-Propyl)-OH (10)


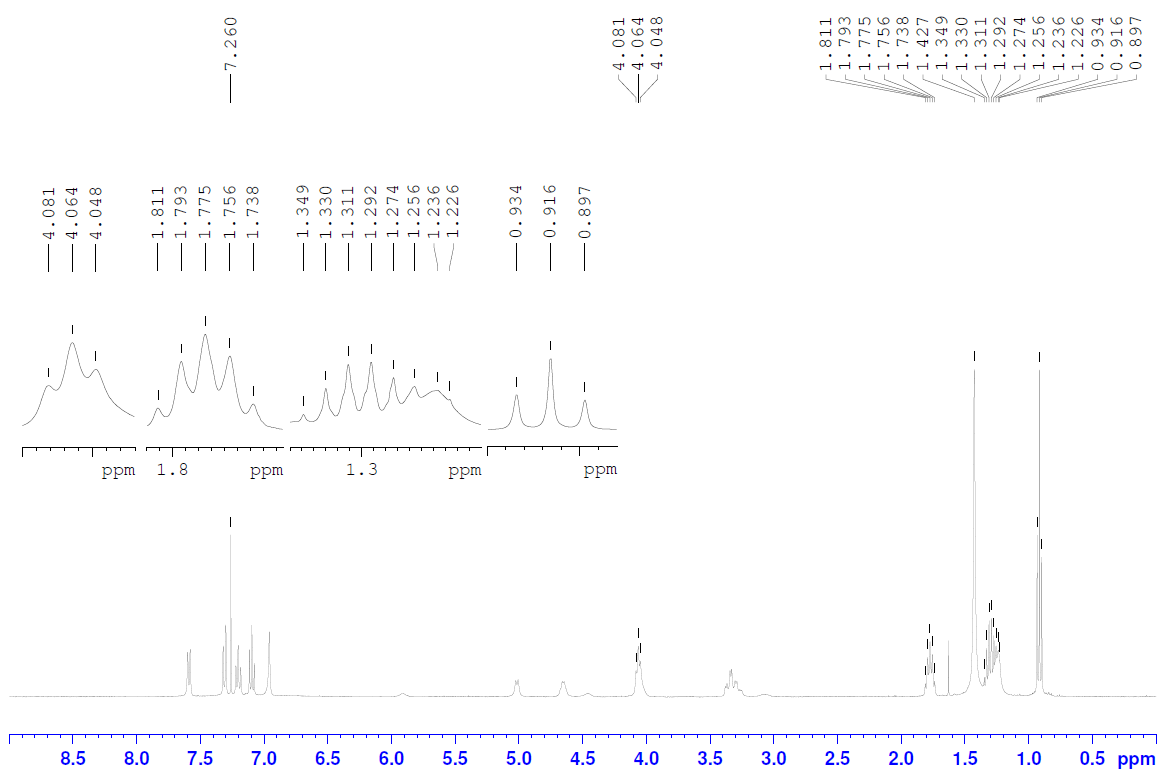

Figure S10a. ^1^H NMR (400 MHz, CDCl_3_) spectra for Boc-D-Trp(N-Butyl)-OH (11)


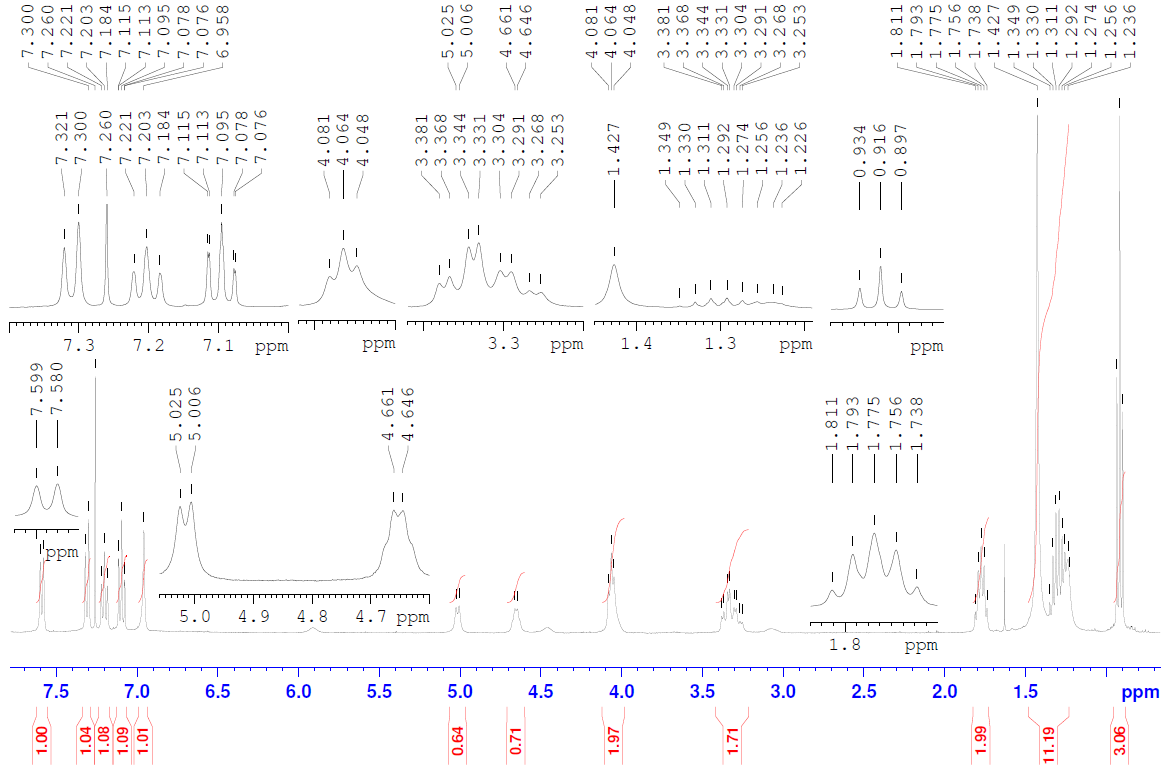


Figure S10b. Expanded ^1^H NMR (400 MHz, CDCl_3_) spectra for Boc-D-Trp(N-Butyl)-OH (11)


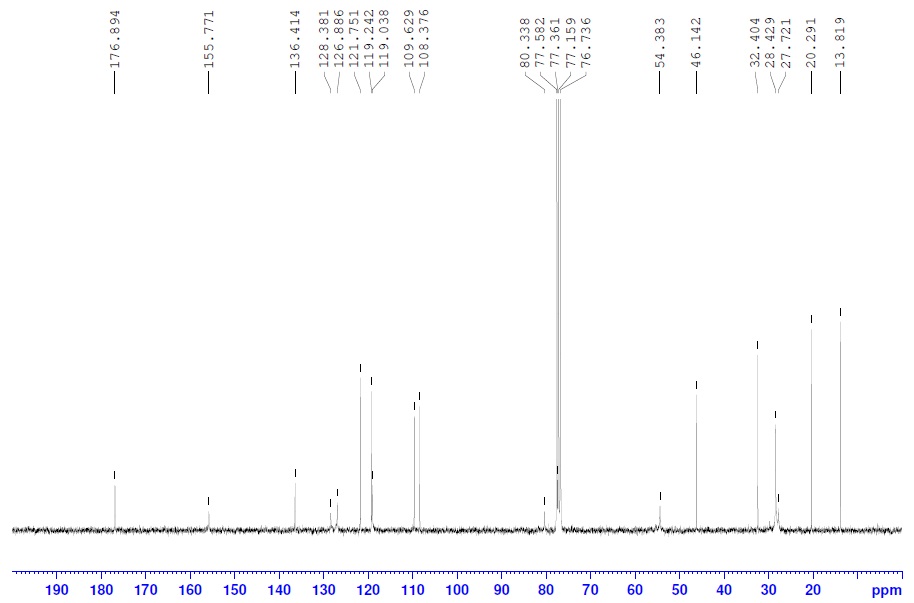

Figure S10c. ^13^C NMR (75 MHz, CDCl_3_) spectra for Boc-D-Trp(N-Butyl)-OH (11)


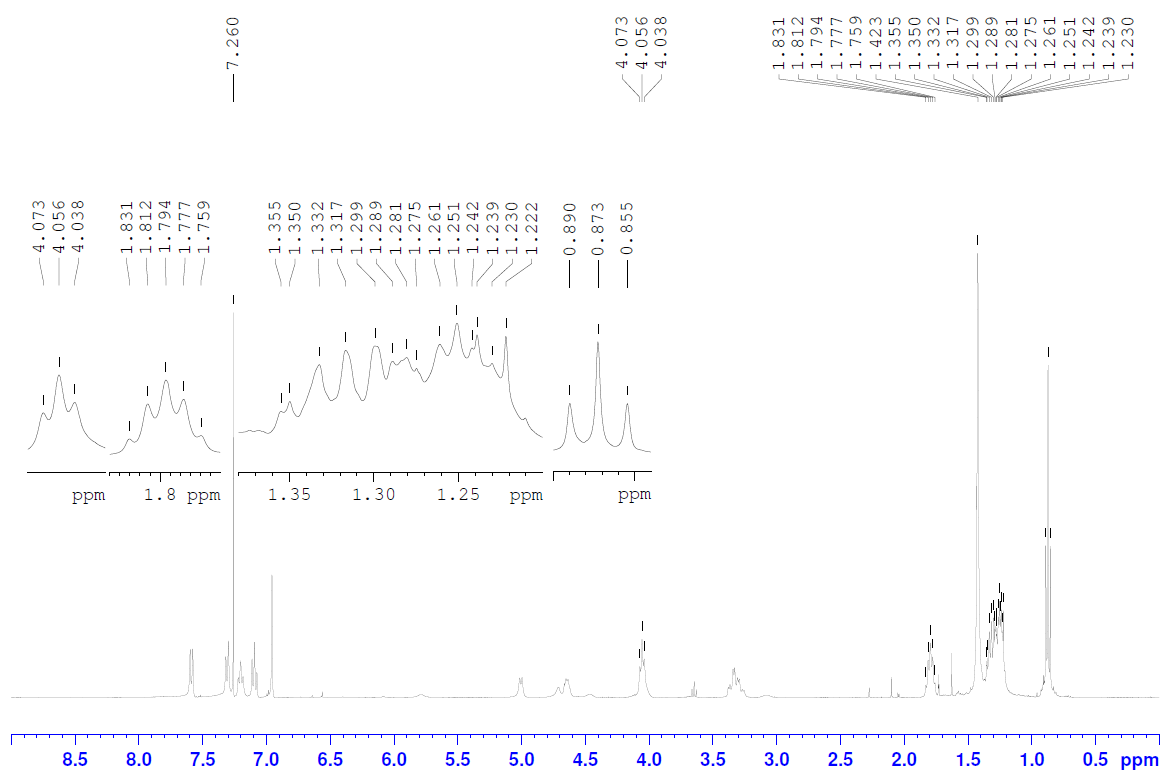

Figure S11a. ^1^H NMR (400 MHz, CDCl_3_) spectra for Boc-D-Trp(N-Pentyl)-OH (12)


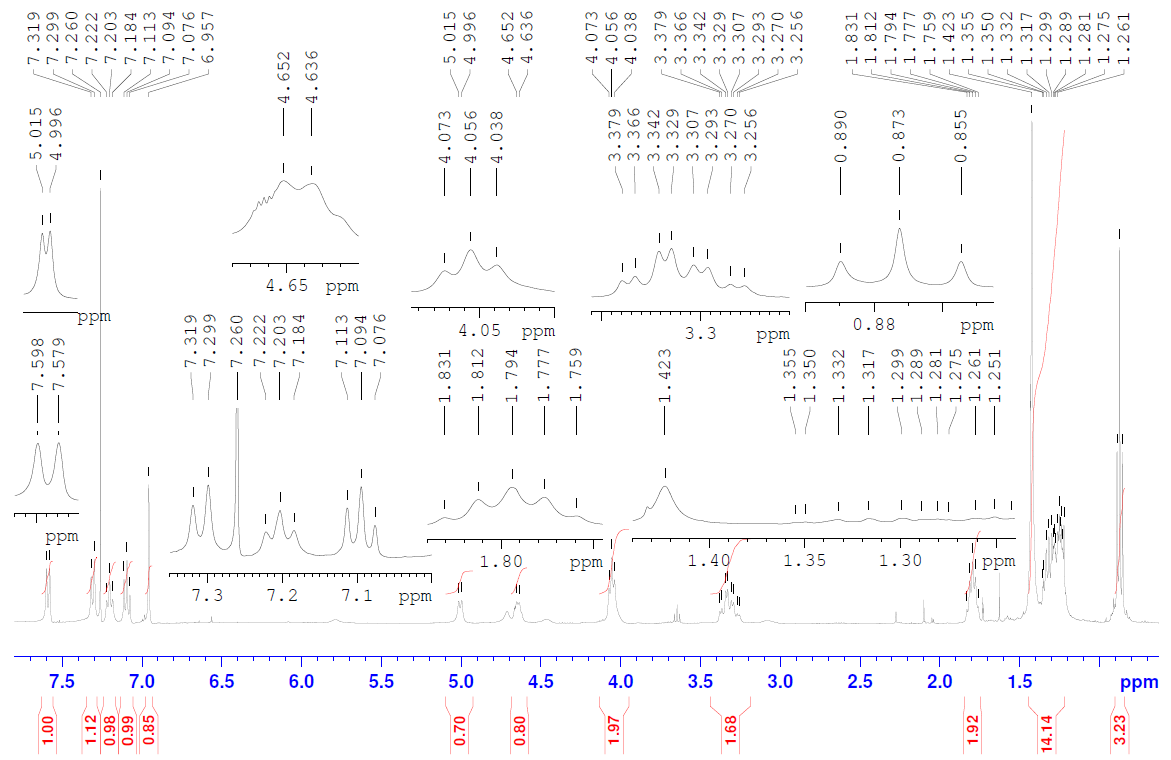


Figure S11b. Expanded ^1^H NMR (400 MHz, CDCl_3_) spectra for Boc-D-Trp(N-Pentyl)-OH (12)


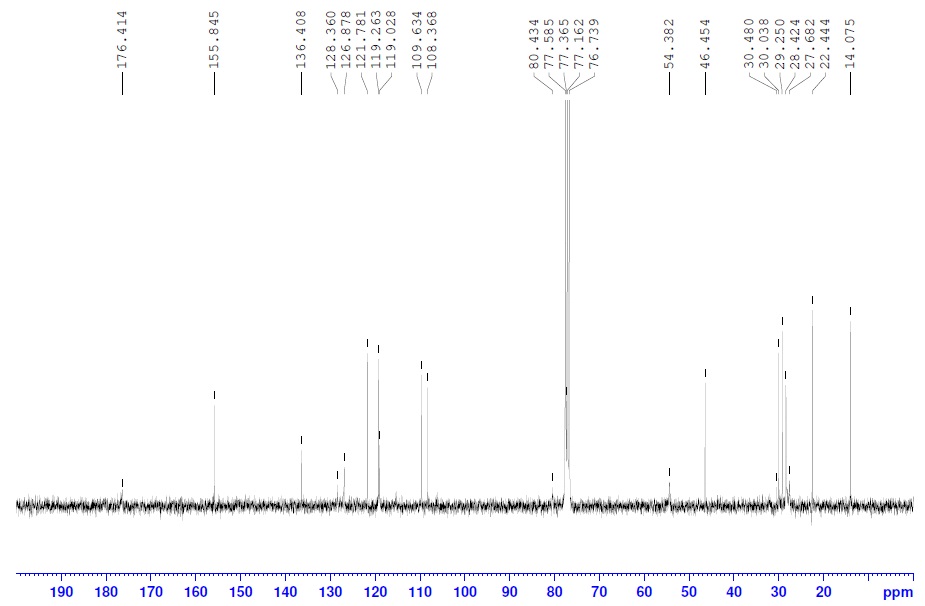

Figure S11c. ^13^C NMR (75 MHz, CDCl_3_) spectra for Boc-D-Trp(N-Pentyl)-OH (12)


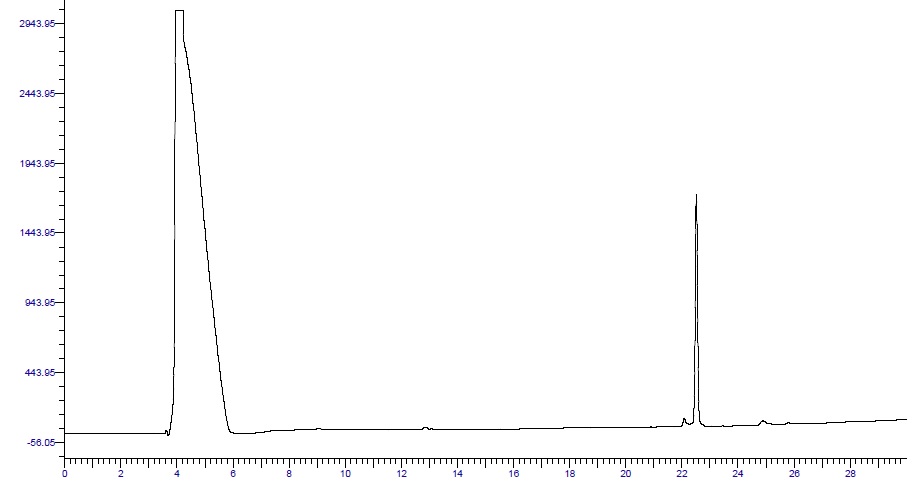

Figure S12. RP-HPLC trace for Methyl^4th^-NH_2_ (13). *t_R_* = 22.51 min, *λ_max_* 220 nm, C8 column, acetic acid/water solvent. Column was eluted with a linear gradient of 0.1% TFA in water and 0.1%TFA in acetonitrile.


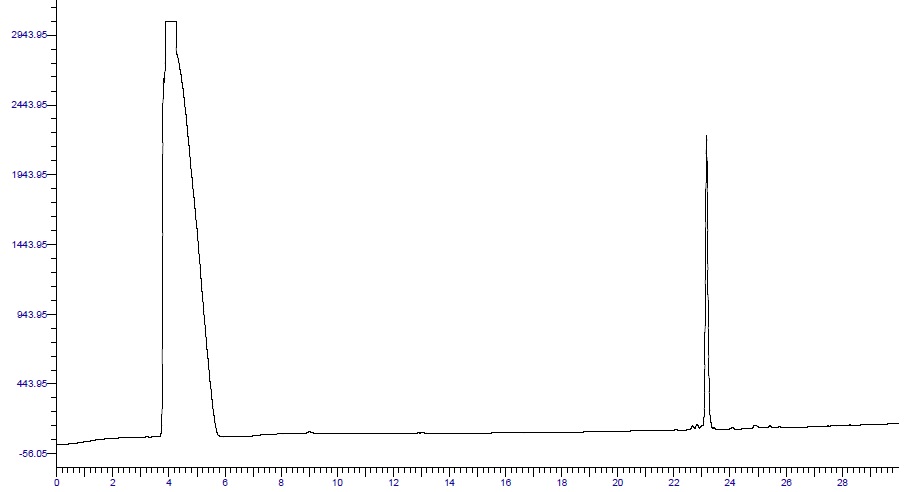

Figure S13. RP-HPLC trace for Ethyl^4th^-NH_2_ (14). *t_R_* = 23.16 min, *λ_max_* 220 nm, C8 column, acetic acid/water solvent. Column was eluted with a linear gradient of 0.1% TFA in water and 0.1%TFA in acetonitrile.


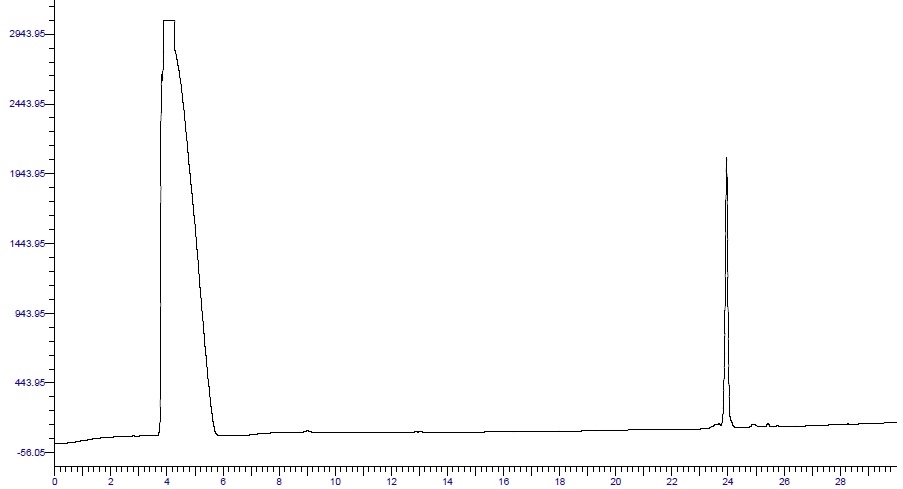

Figure S14. RP-HPLC trace for Propyl^4th^-NH_2_ (15). *t_R_* = 23.95 min, *λ_max_* 220 nm, C8 column, acetic acid/water solvent. Column was eluted with a linear gradient of 0.1% TFA in water and 0.1%TFA in acetonitrile.


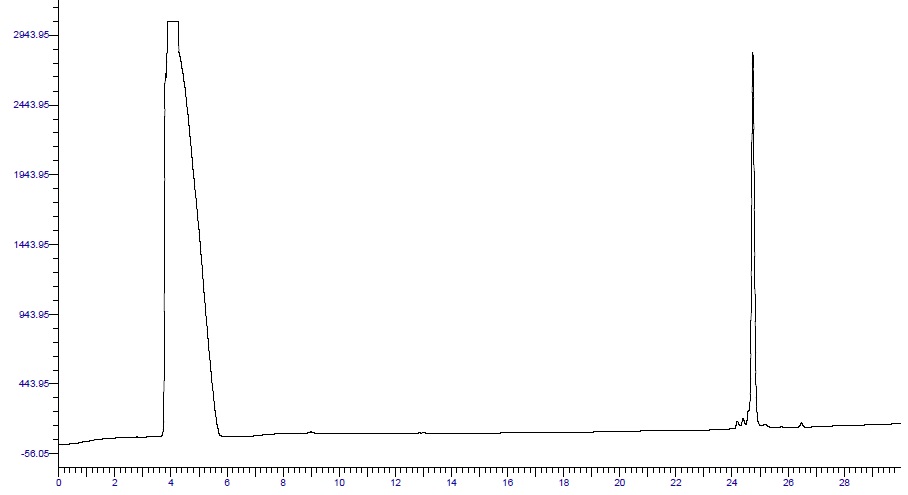

Figure S15. RP-HPLC trace for Butyl^4th^-NH_2_ (16). *t_R_* = 24.74 min, *λ_max_* 220 nm, C8 column, acetic acid/water solvent. Column was eluted with a linear gradient of 0.1% TFA in water and 0.1%TFA in acetonitrile.


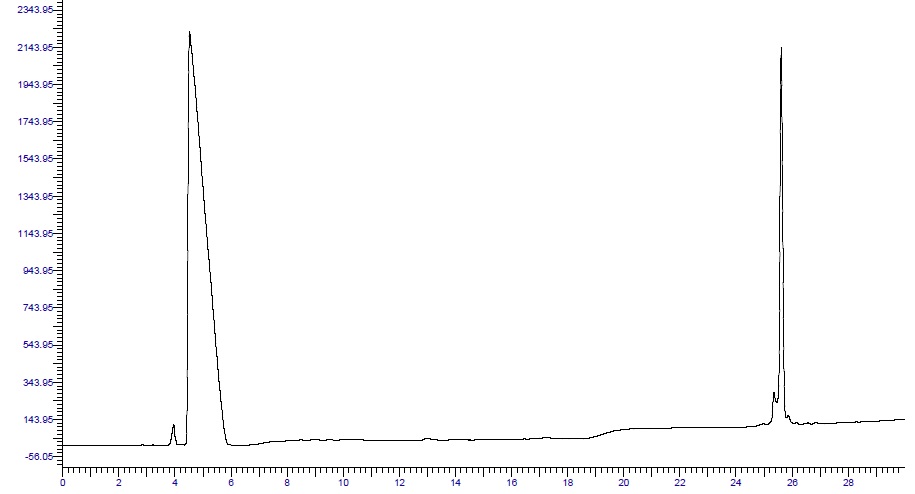

Figure S16. RP-HPLC trace for Pentyl^4th^-NH_2_ (17). *t_R_* = 25.61 min, *λ_max_* 220 nm, C8 column, acetic acid/water solvent. Column was eluted with a linear gradient of 0.1% TFA in water and 0.1%TFA in acetonitrile.


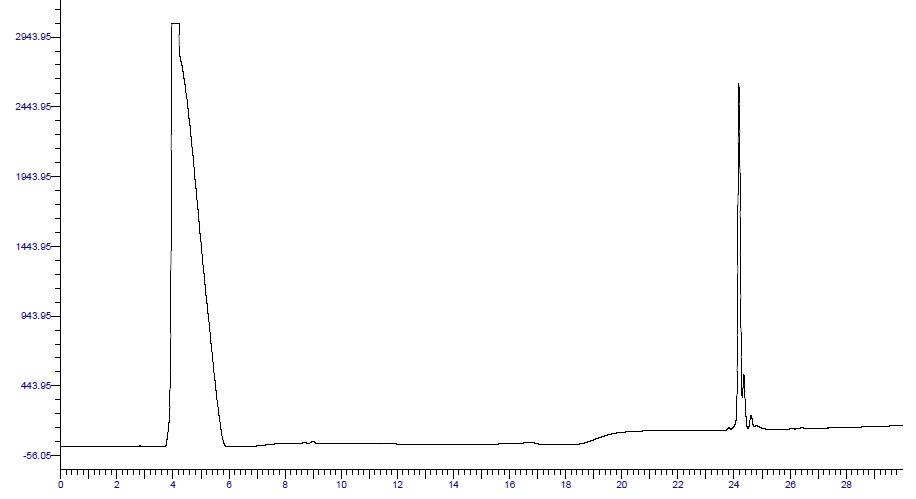

Figure S17. RP-HPLC trace for Butyl^2nd^-NH_2_ (18). *t_R_* = 24.17 min, *λ_max_* 220 nm, C8 column, acetic acid/water solvent. Column was eluted with a linear gradient of 0.1% TFA in water and 0.1%TFA in acetonitrile.


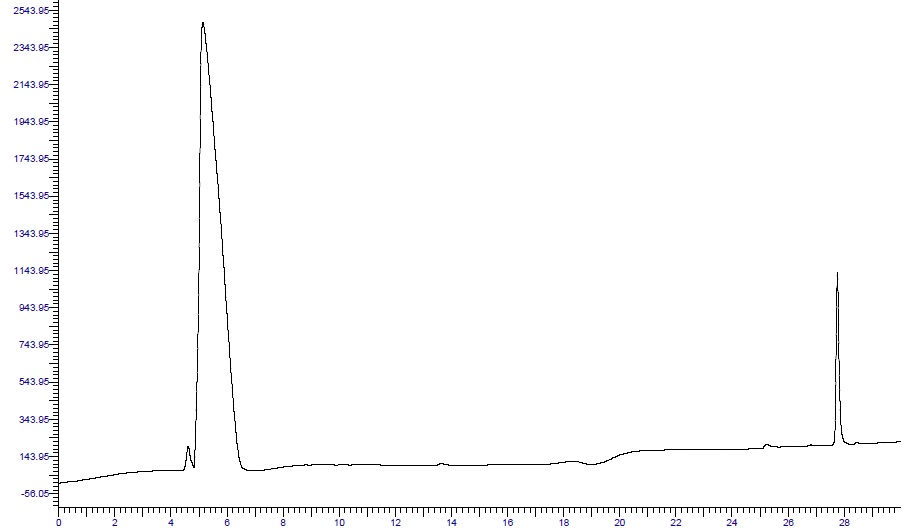

Figure S18. RP-HPLC trace for Butyl^2nd,4th^-NH_2_ (19). *t_R_* = 27.75 min, *λ_max_* 220 nm, C8 column, acetic acid/water solvent. Column was eluted with a linear gradient of 0.1% TFA in water and 0.1%TFA in acetonitrile.


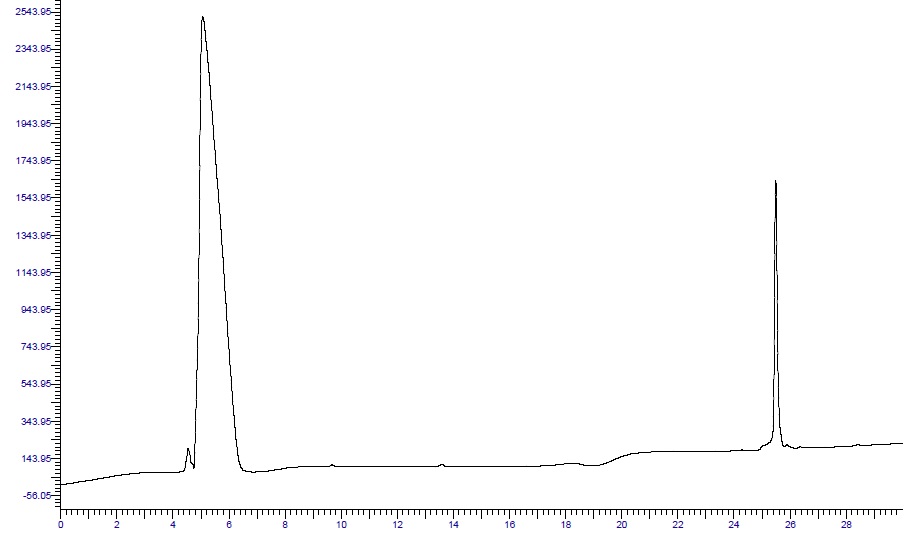

Figure S19. RP-HPLC trace for Butyl^4th^-OH (20). *t_R_* = 25.49 min, *λ_max_* 220 nm, C8 column, acetic acid/water solvent. Column was eluted with a linear gradient of 0.1% TFA in water and 0.1%TFA in acetonitrile.

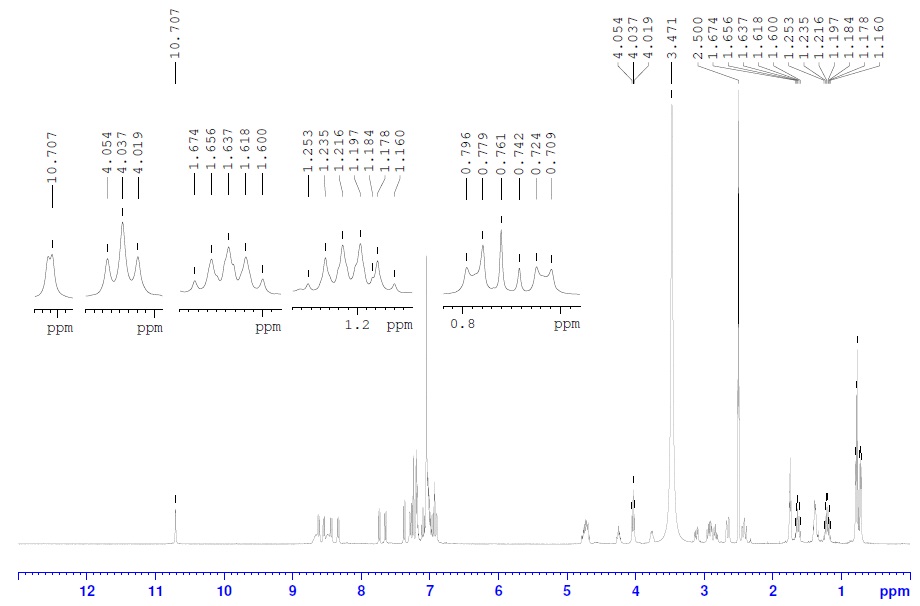


Figure S20a. ^1^H NMR (400 MHz, DMSO-d_6_) spectrum for Butyl^4th^-NH_2_ (16)


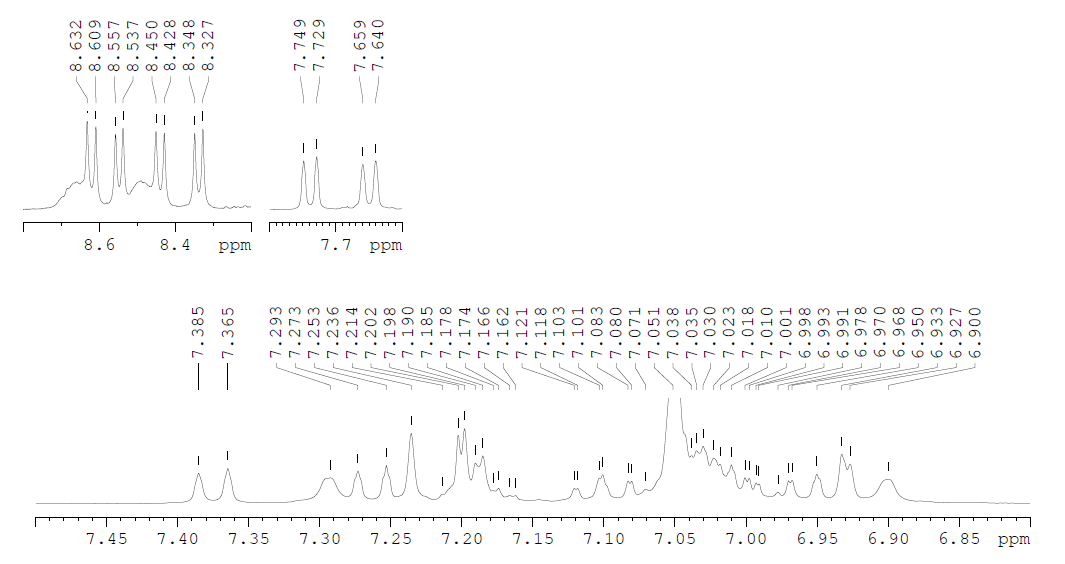

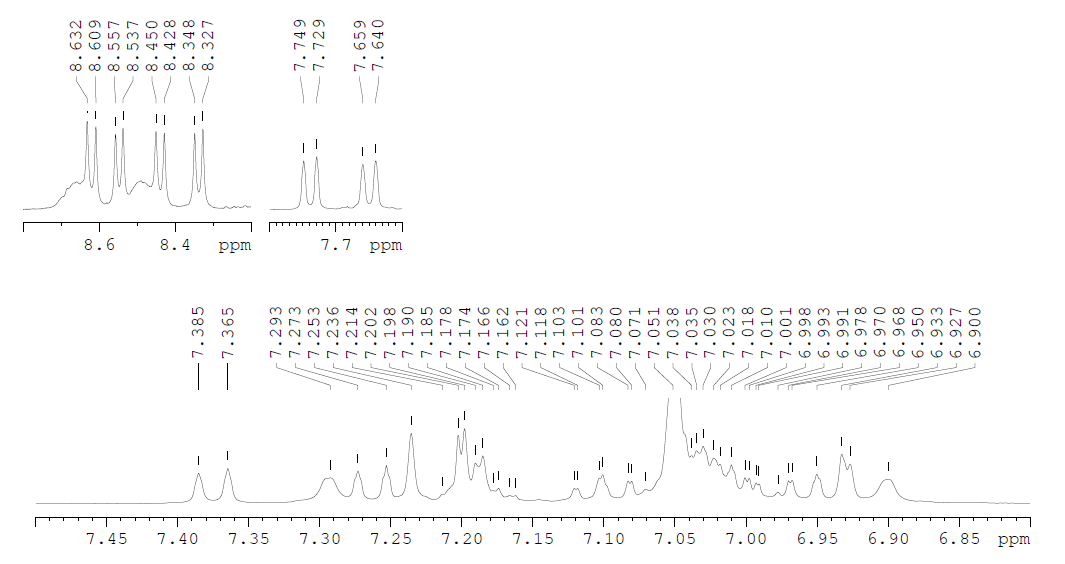


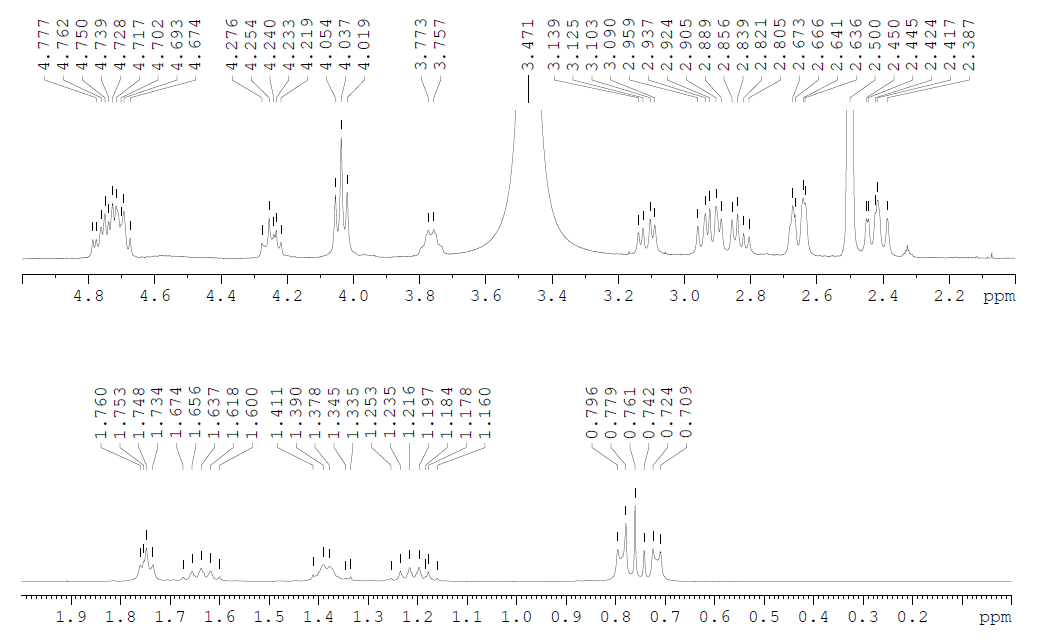


Figure S20b. Expanded ^1^H NMR (400 MHz, DMSO-d_6_) spectrum for Butyl^4th^-NH_2_ (16)

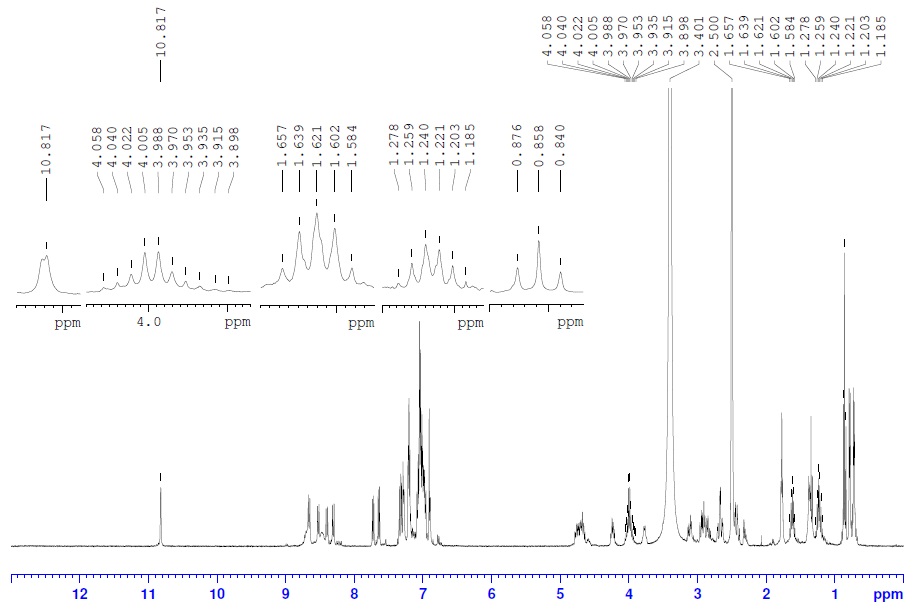


Figure S21a. ^1^H NMR (400 MHz, DMSO-d_6_) spectrum for Butyl^2nd^-NH_2_ (18)


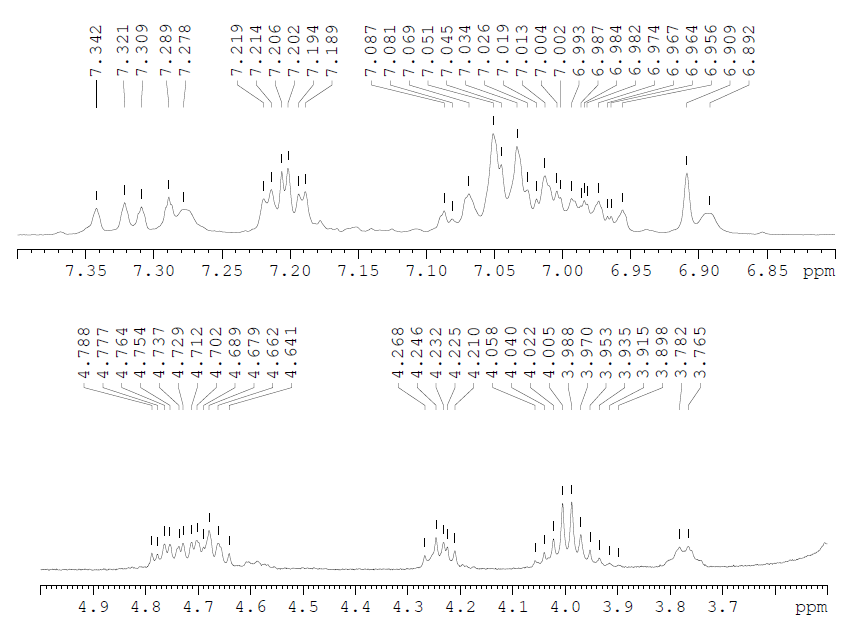


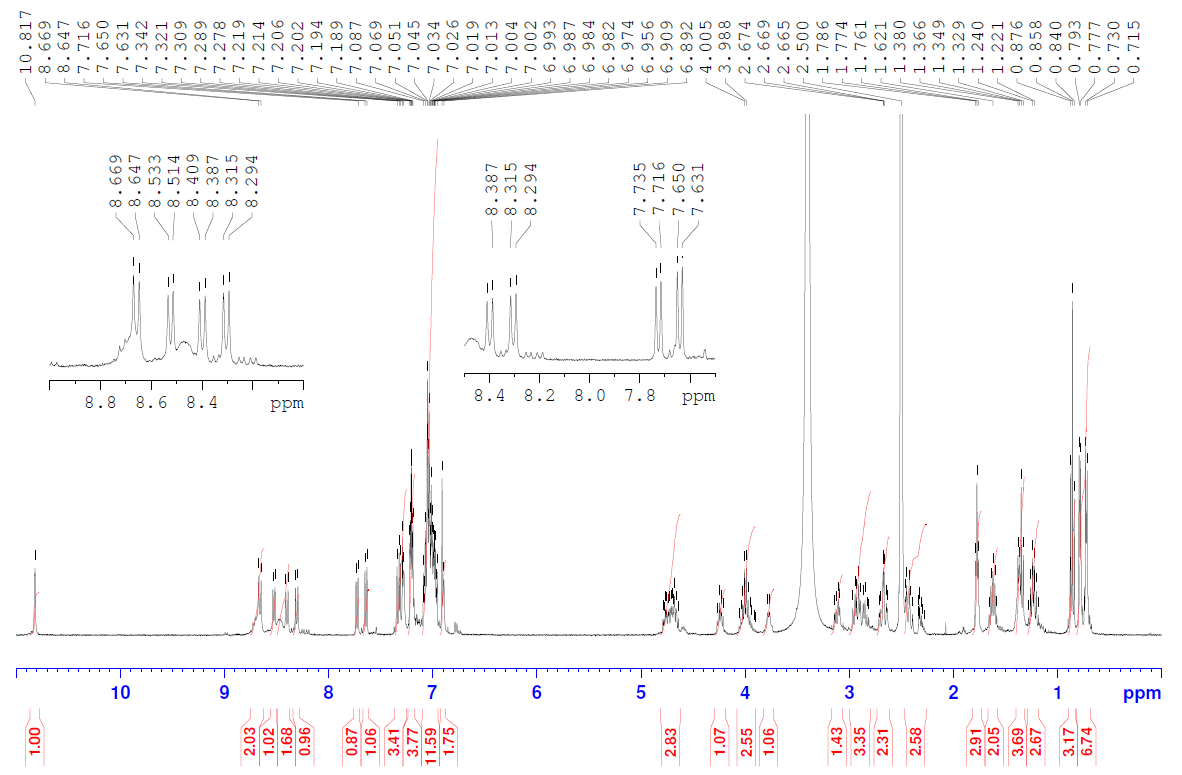

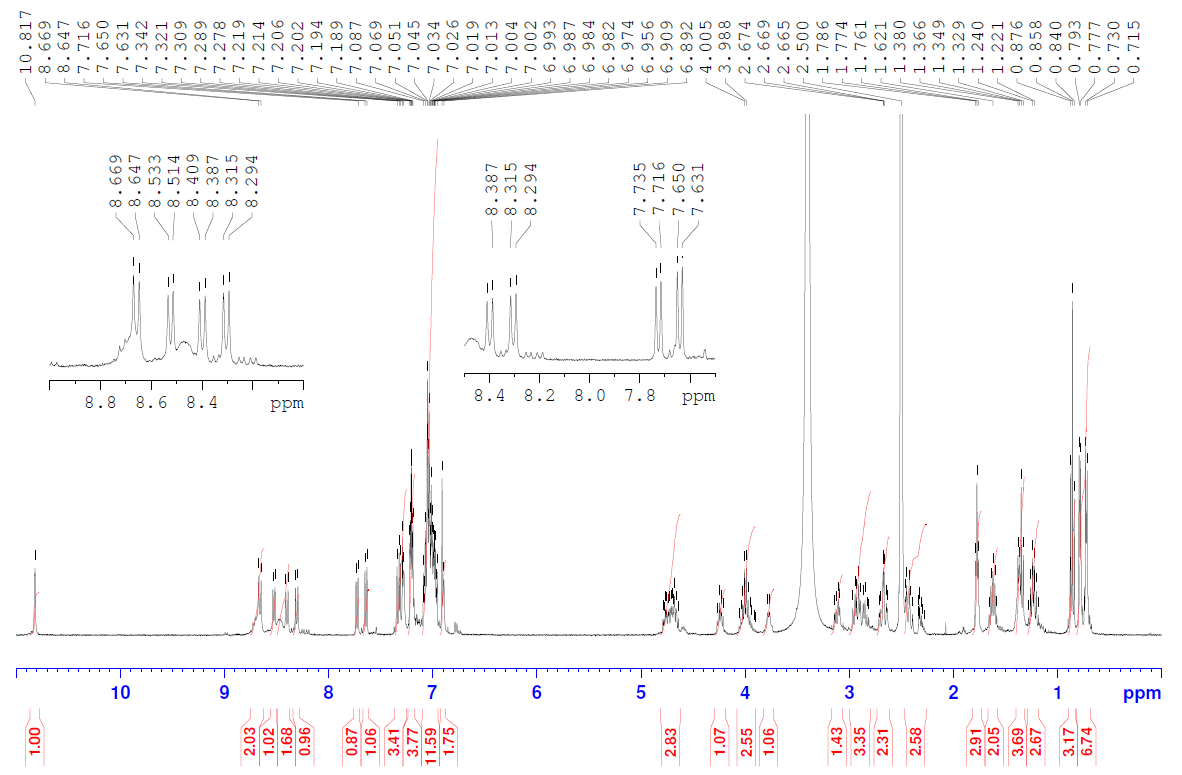


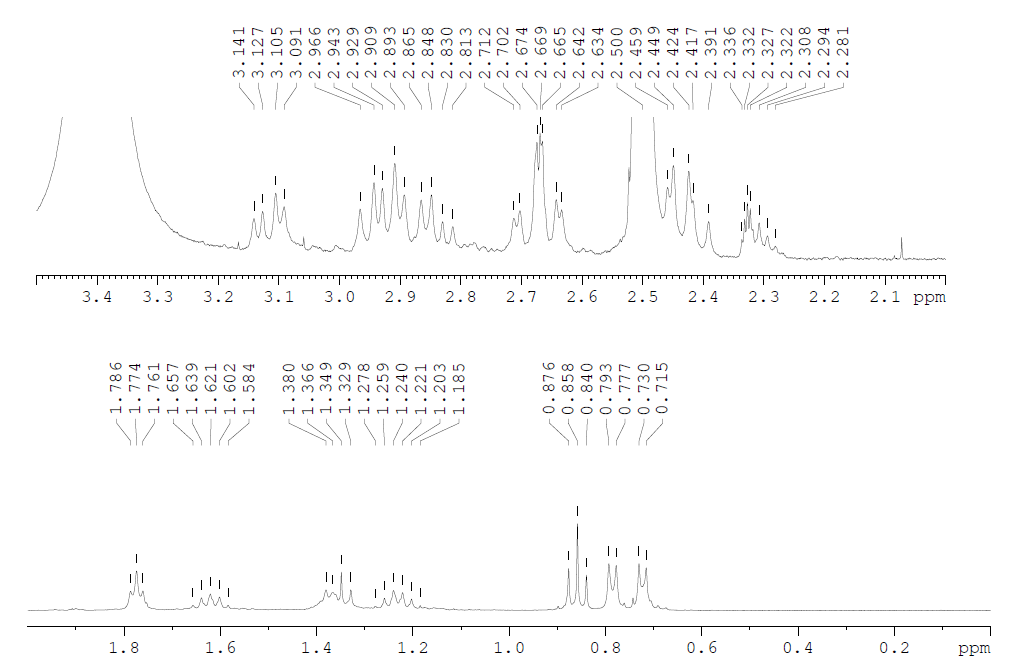

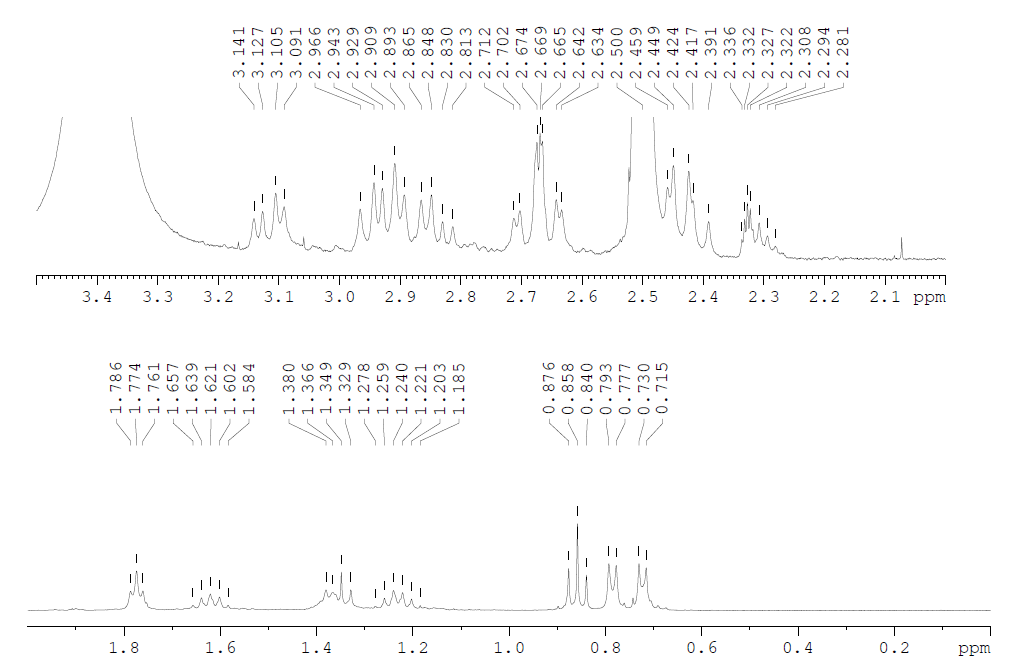


Figure S21b. Expanded ^1^H NMR (400 MHz, DMSO-d_6_) spectrum for Butyl^2nd^-NH_2_ (18)

**
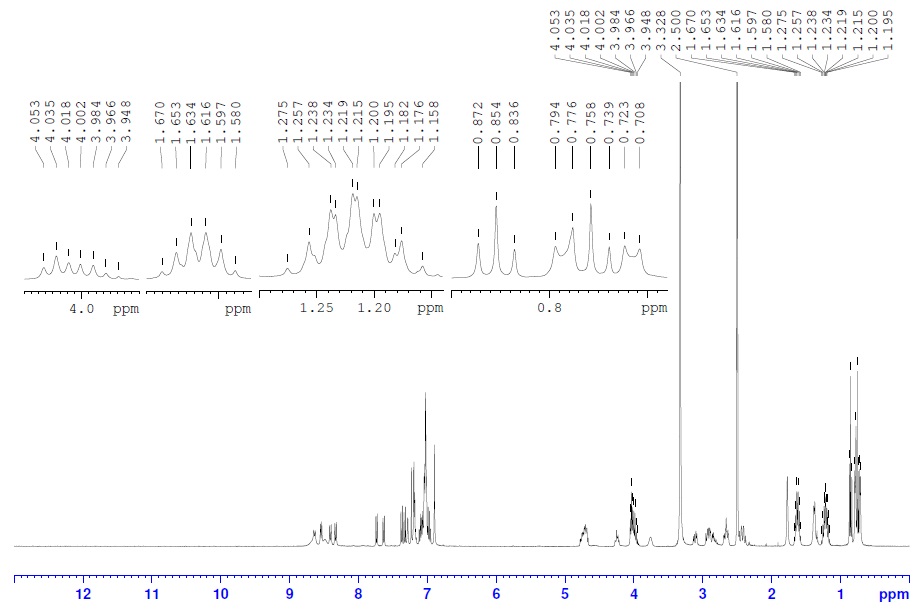
**

Figure S22a. ^1^H NMR (400 MHz, DMSO-d_6_) spectrum for Butyl^2nd,4th^-NH_2_ (19)


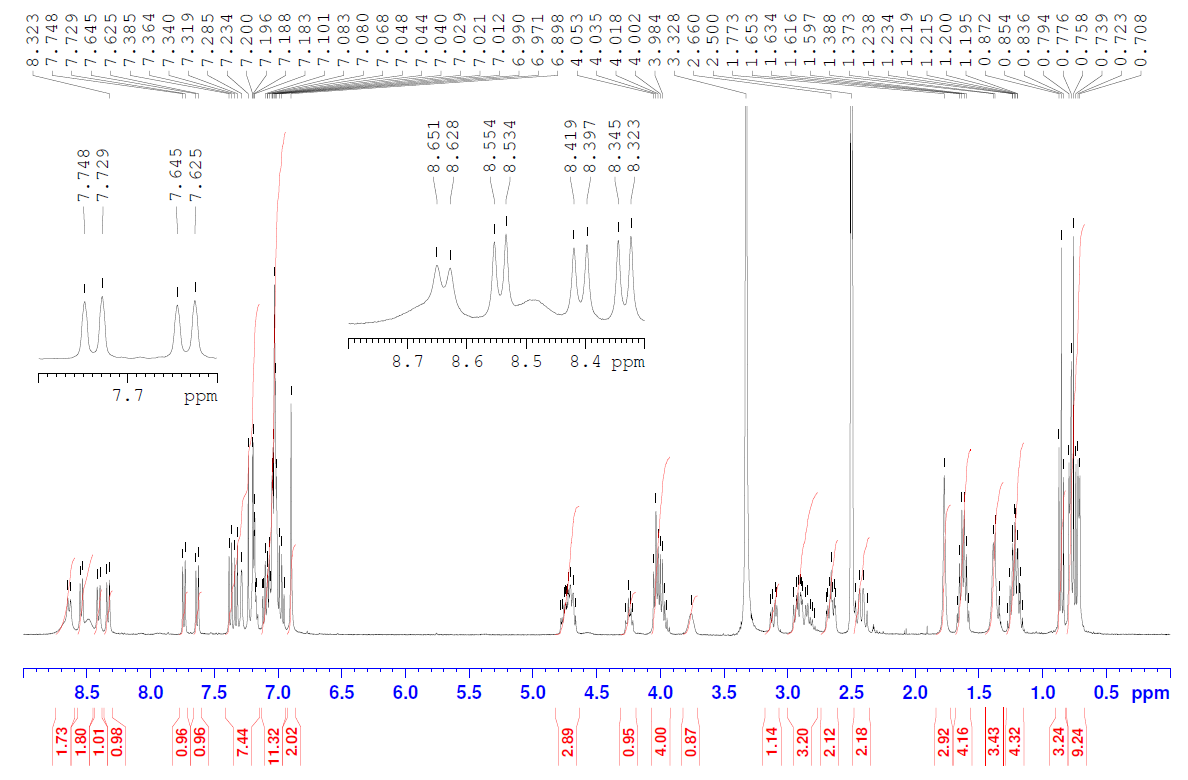

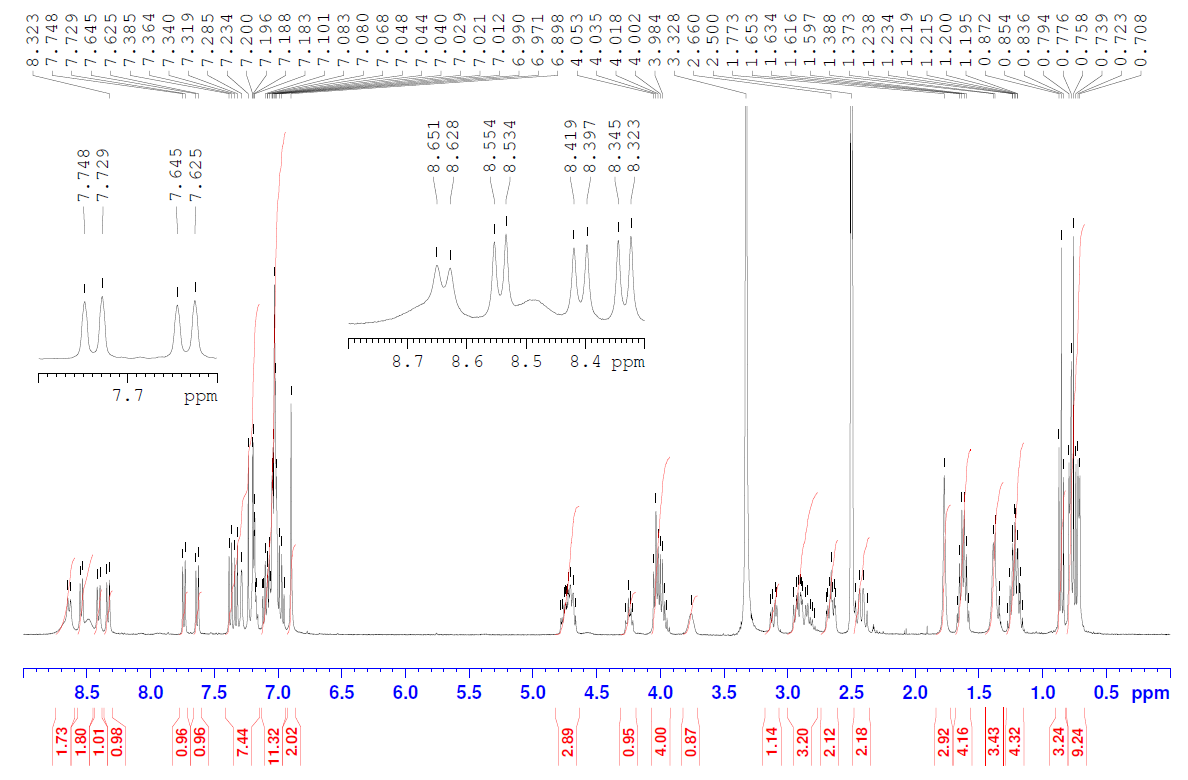

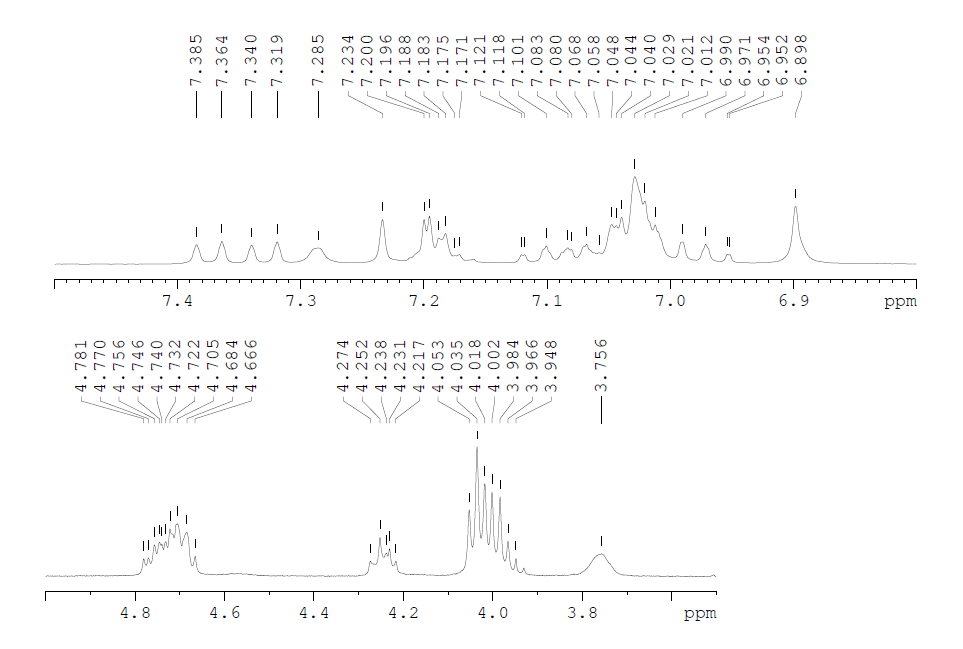


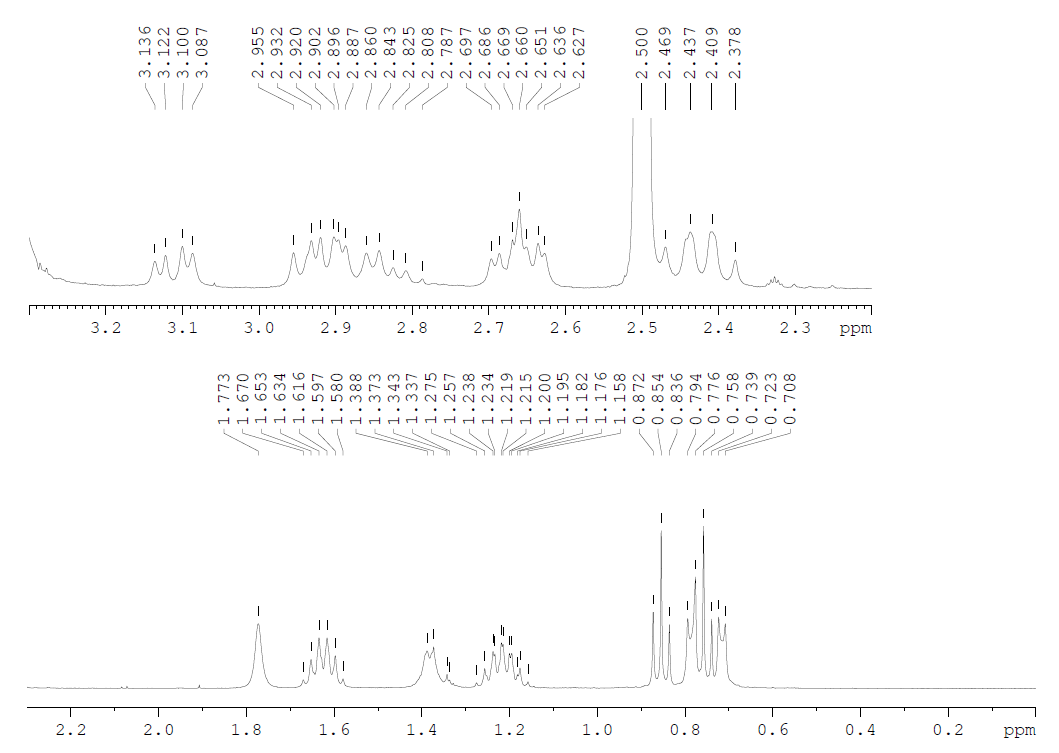

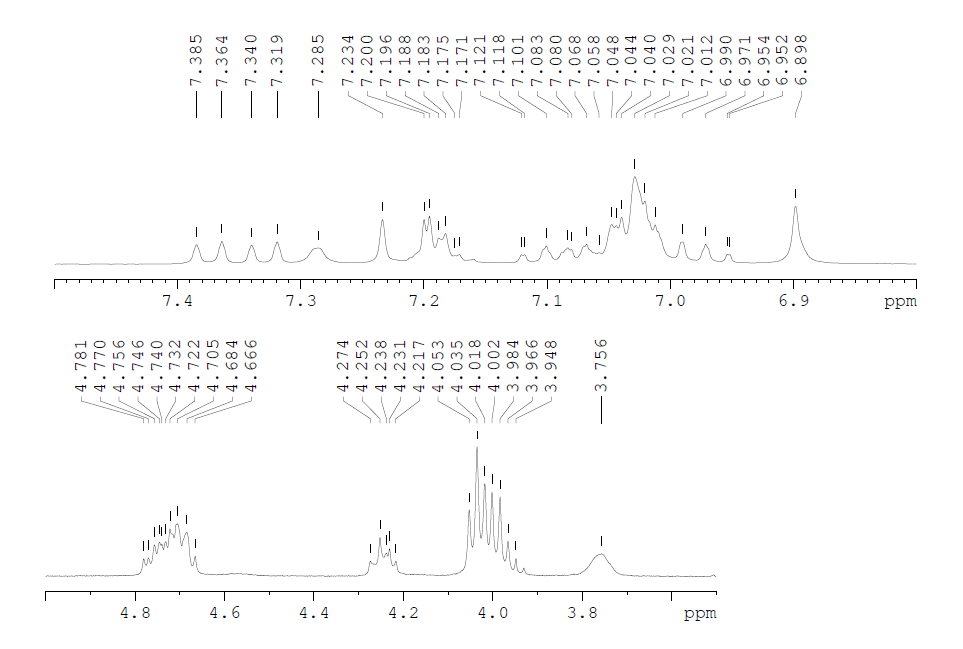


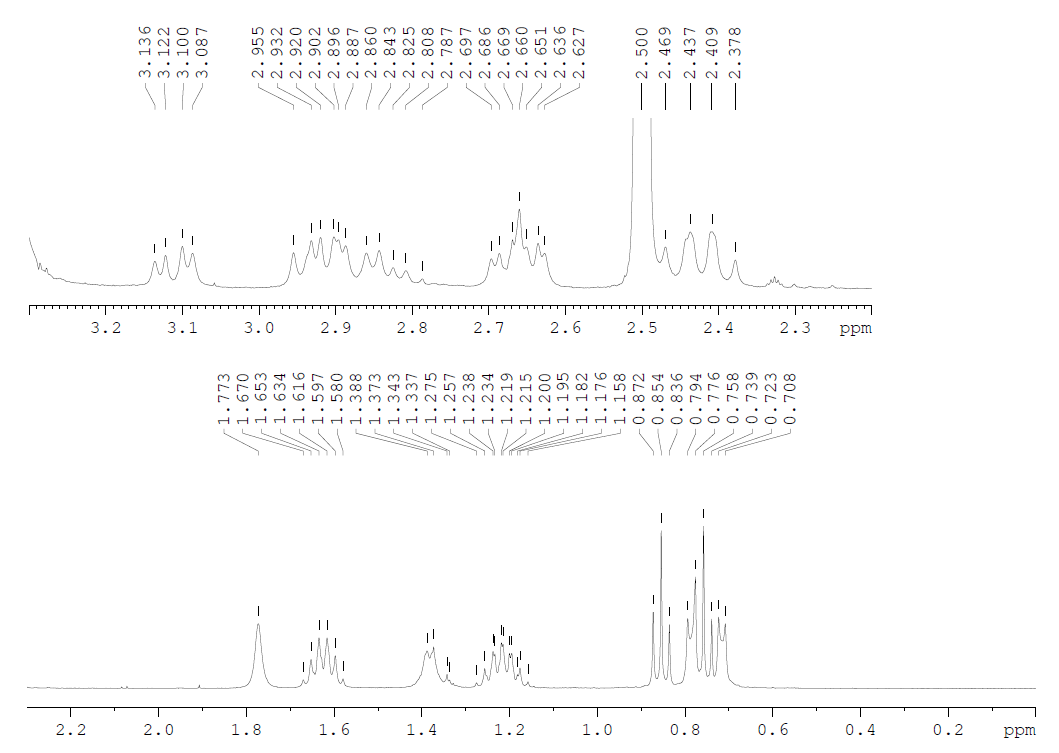


Figure S22b. Expanded ^1^H NMR (400 MHz, DMSO-d_6_) spectrum for Butyl^2nd,4th^-NH_2_ (19)

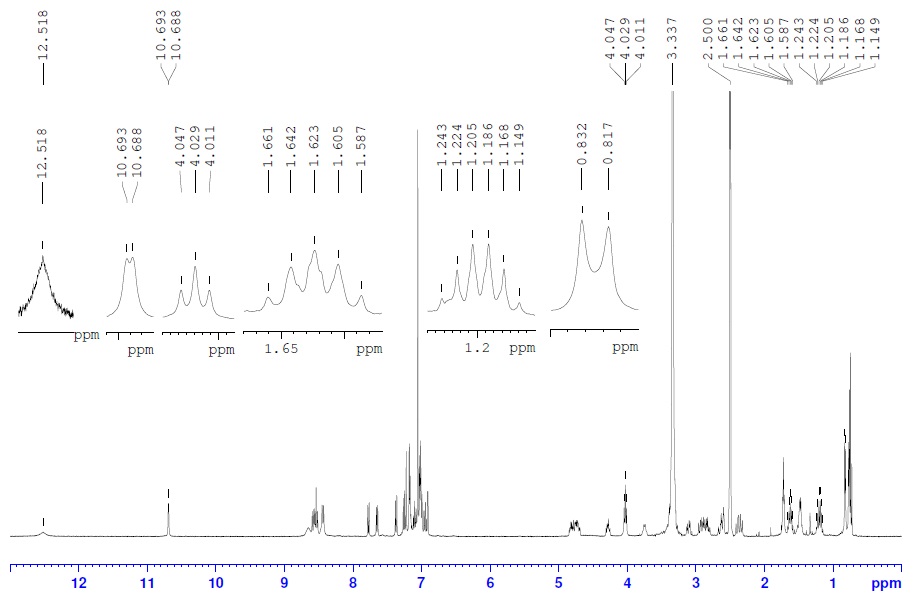


Figure S23a. ^1^H NMR (400 MHz, DMSO-d_6_) spectrum for Butyl^4th^-OH (20)


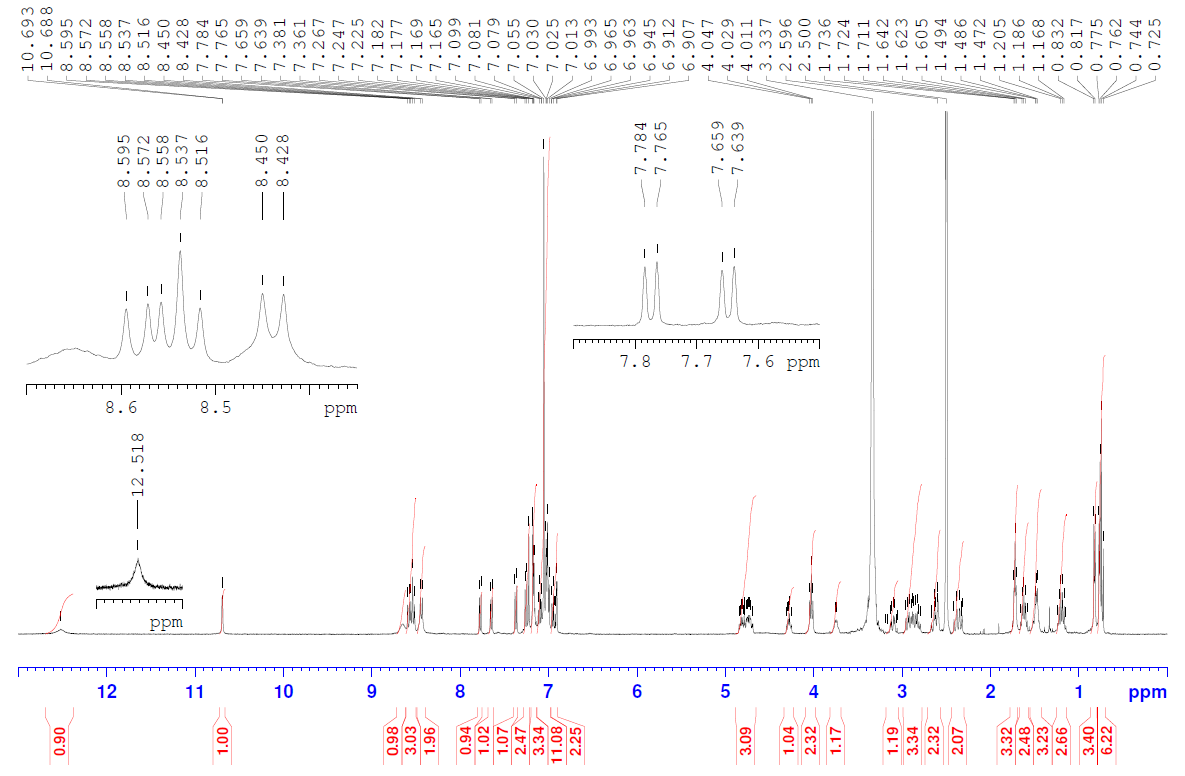

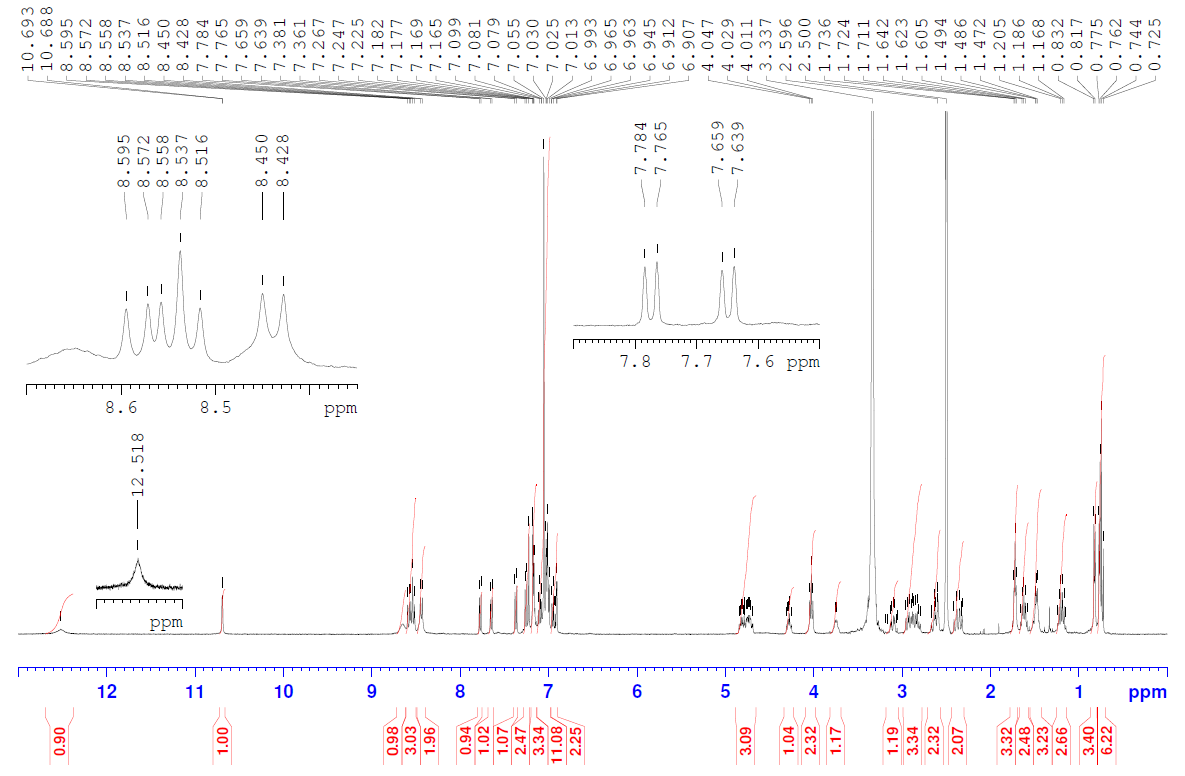

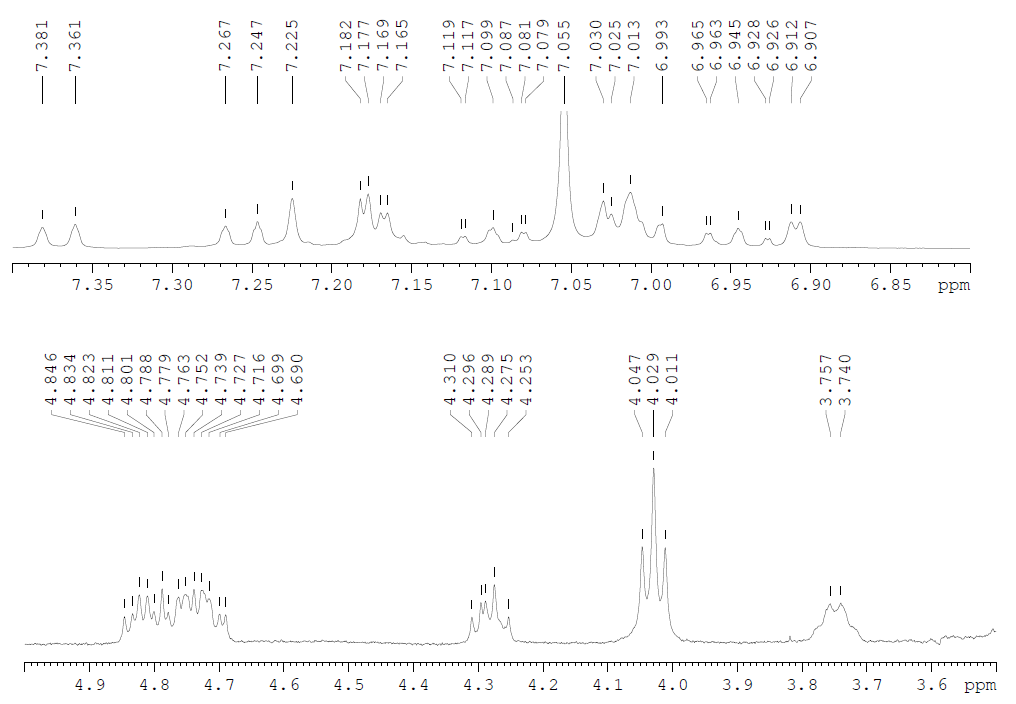


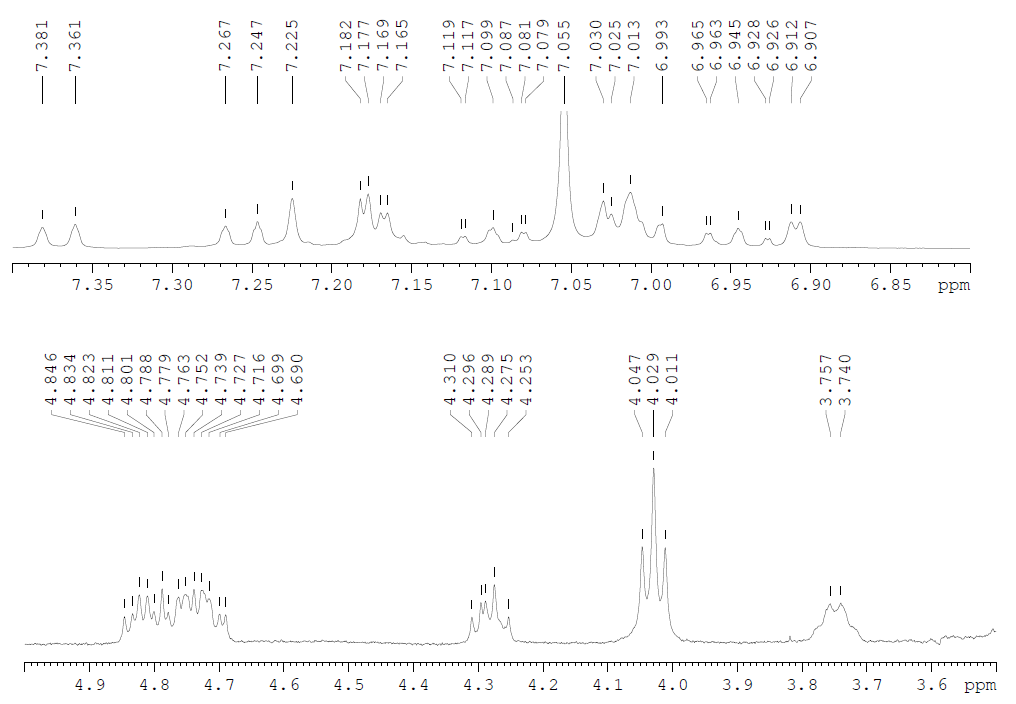

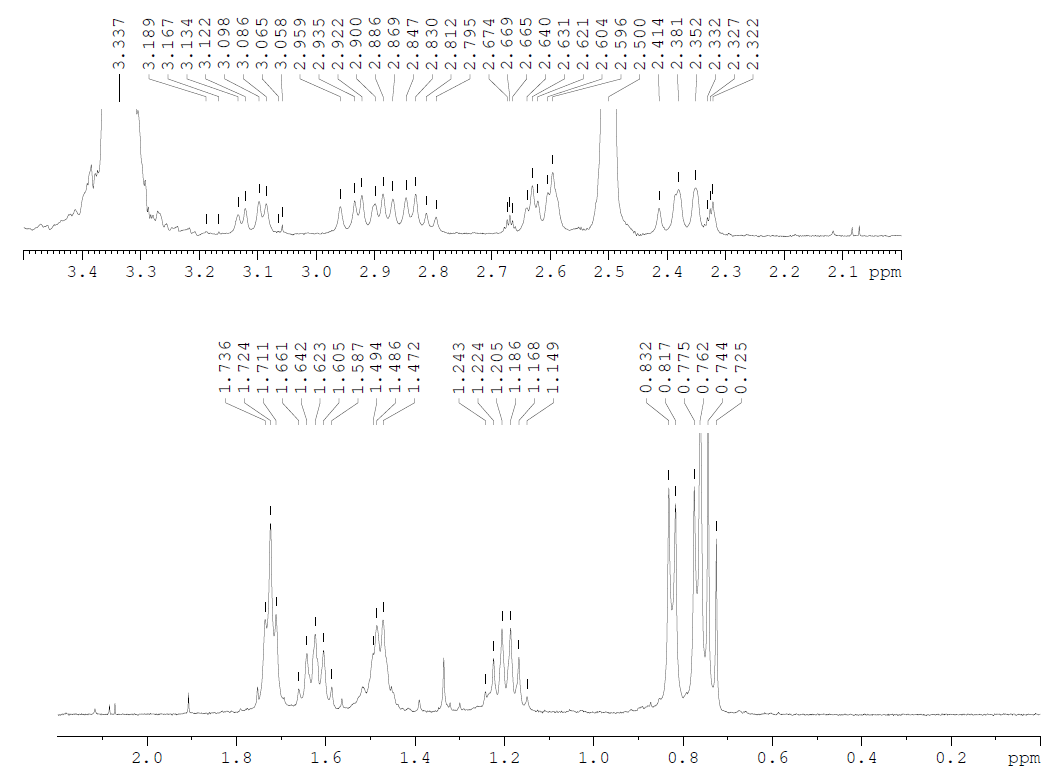


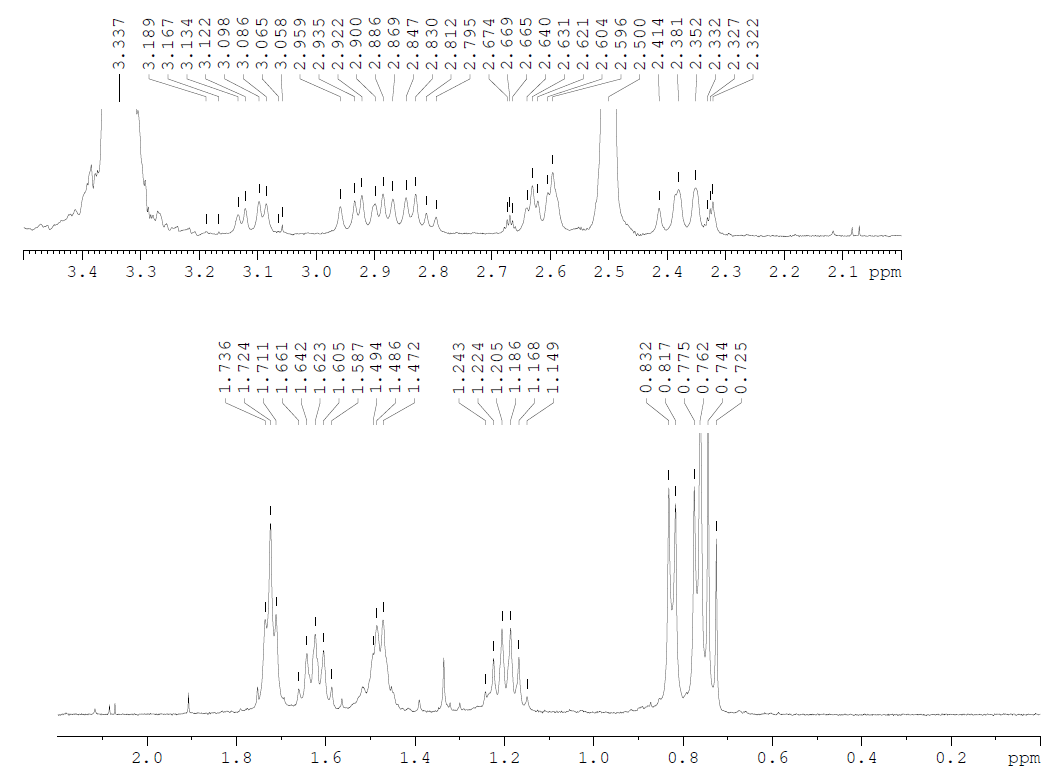


Figure S23b. Expanded ^1^H NMR (400 MHz, DMSO-d_6_) spectrum for Butyl^4th^-OH (20)

Figure S24. Dose-response curves of the cell viability assays on H69 and DMS79 cells for

peptides 13-20


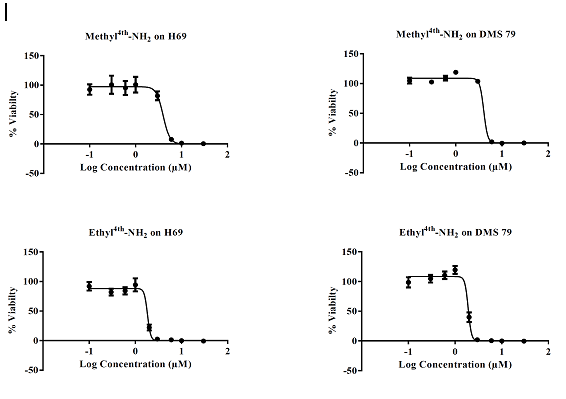


**13**

**13**


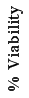

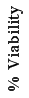


**IC_50_ = 4.03 µM**


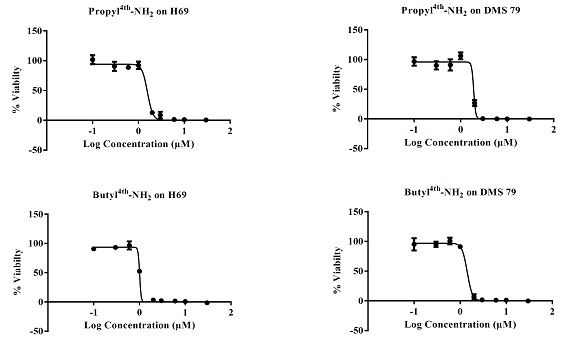


**IC_50_ = 3.98 µM**

**14**


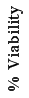


**14**


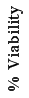


**IC_50_ = 1.92 µM**

**IC_50_ = 1.83 µM**

**15**

**15**


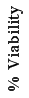

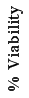


**IC_50_ = 1.91 µM**

**IC_50_ = 1.57 µM**

**16**

**16**


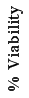

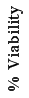


**IC_50_ = 1.43 µM**

**IC_50_ = 1.01 µM**


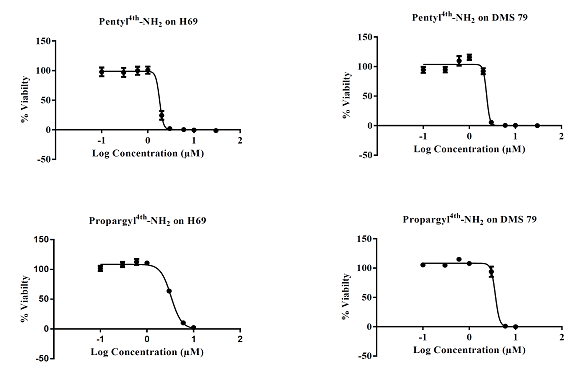


**17**

**17**

**17**


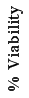

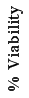


**IC_50_ = 1.80 µM**

**IC_50_ = 2.37 µM**


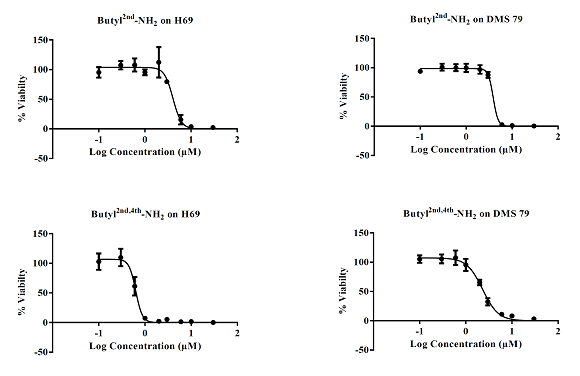


**18**

**18**


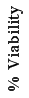

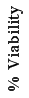


**IC_50_ = 3.88 µM**

**IC_50_ = 4.05 µM**

**19**

**19**


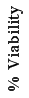

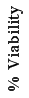


**IC_50_ = 0.63 µM**

**IC_50_ = 2.31 µM**


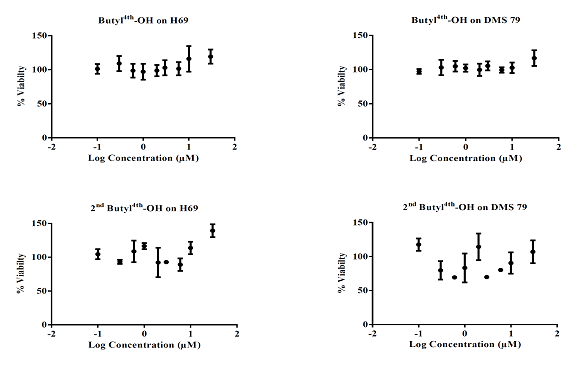


**20**

**20**


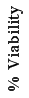

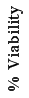


**IC_50_ > 30 µM**

**IC_50_ > 30 µM**

**The stability of selected peptides in mouse plasma**

**Table S1.**

**Relative % (± SE) of peptides 2, 16, 19 and 20 remained after each time interval during plasma stability studies.**

|  | **% (± SE) of parent peptide**  **to degradation product(s) in plasma studies** | | | |
| --- | --- | --- | --- | --- |
|  | **0 hr** | **3 hrs** | **24 hrs** | **48 hrs** |
| **2** | 100 | 87.77 ± 1.47 | 57.73 ± 0.83 | 35.87 ± 1.74 |
| **16** | 100 | 94.30 ± 0.20 | 80.30 ± 0.32 | 68.47 ± 0.23 |
| **19** | 100 | 98.67 ± 0.69 | 89.73 ± 0.68 | 81.00 ± 2.25 |
| **20** | 100 | 100 | 100 | 100 |

***tert*-Prenyl^4th^-NH_2_ in mouse plasma**

**
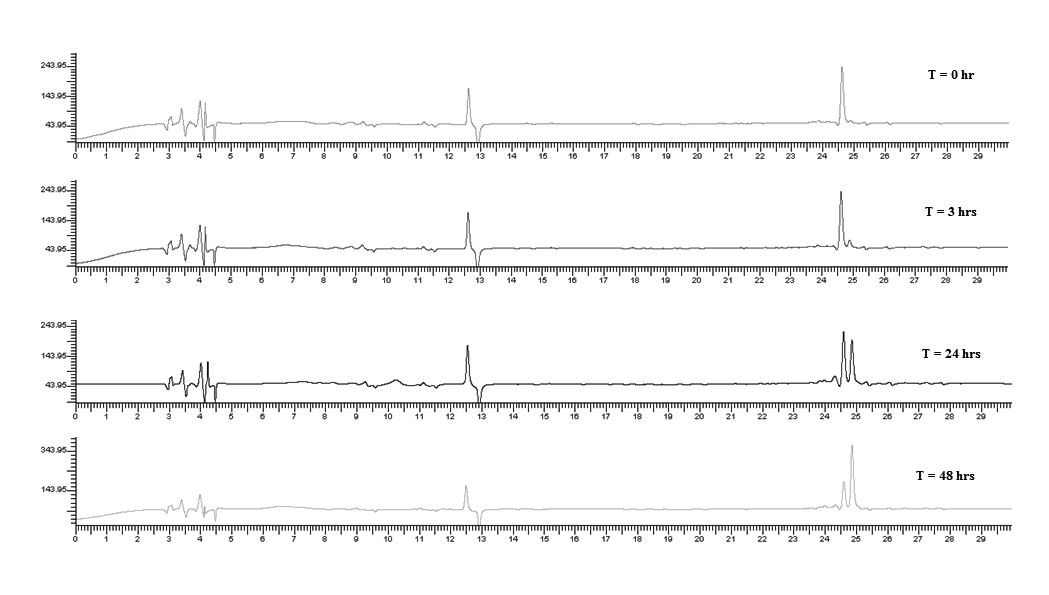
**

Figure S25. RP-HPLC traces for *tert*-Prenyl^4th^-NH_2_ peptide (2) at 0/3/24/48 hrs for mouse plasma stability study.

**Butyl^4th^-NH_2_ in mouse plasma**

**
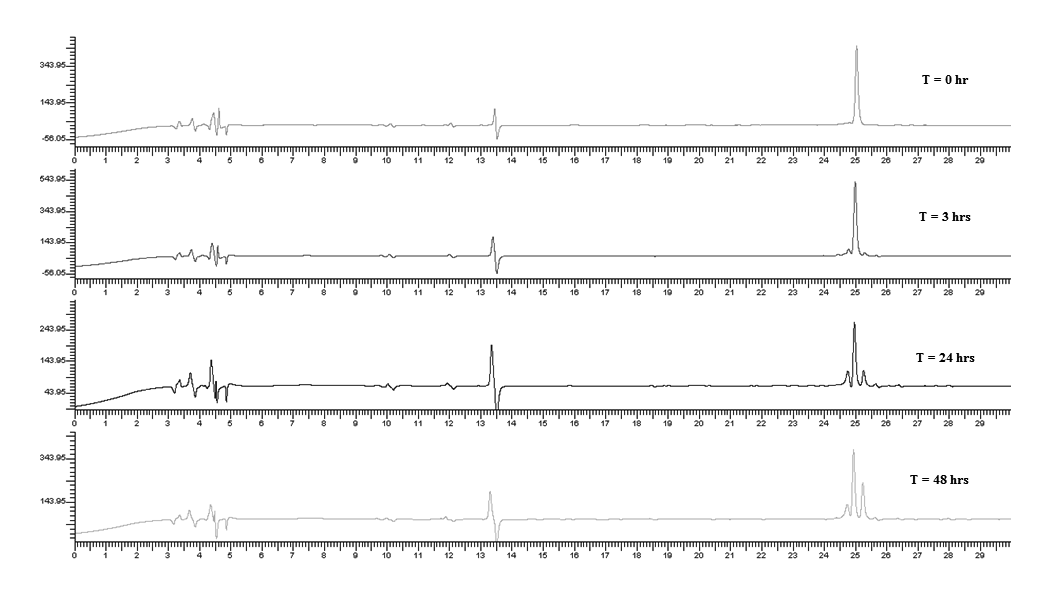
**

Figure S26. RP-HPLC traces for Butyl^4th^-NH_2_ peptide (16) at 0/3/24/48 hrs for mouse plasma stability study.

**Butyl^2nd,4th^-NH_2_ in mouse plasma**


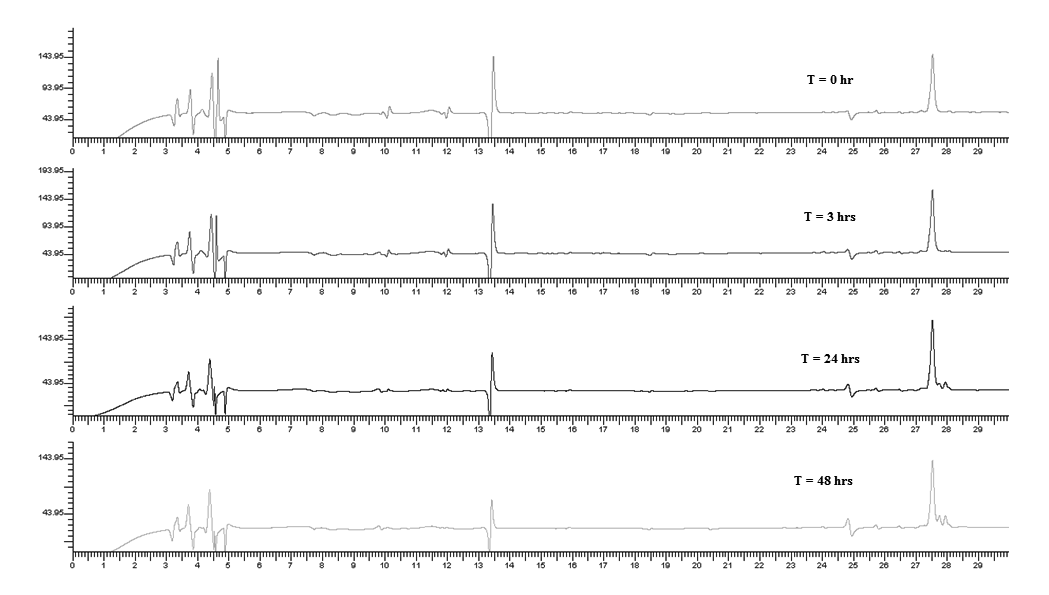


Figure S27. RP-HPLC traces for Butyl^2nd,4th^-NH_2_ peptide (19) at 0/3/24/48 hrs for mouse plasma stability study.

**Butyl^4th^-OH in mouse plasma**

**
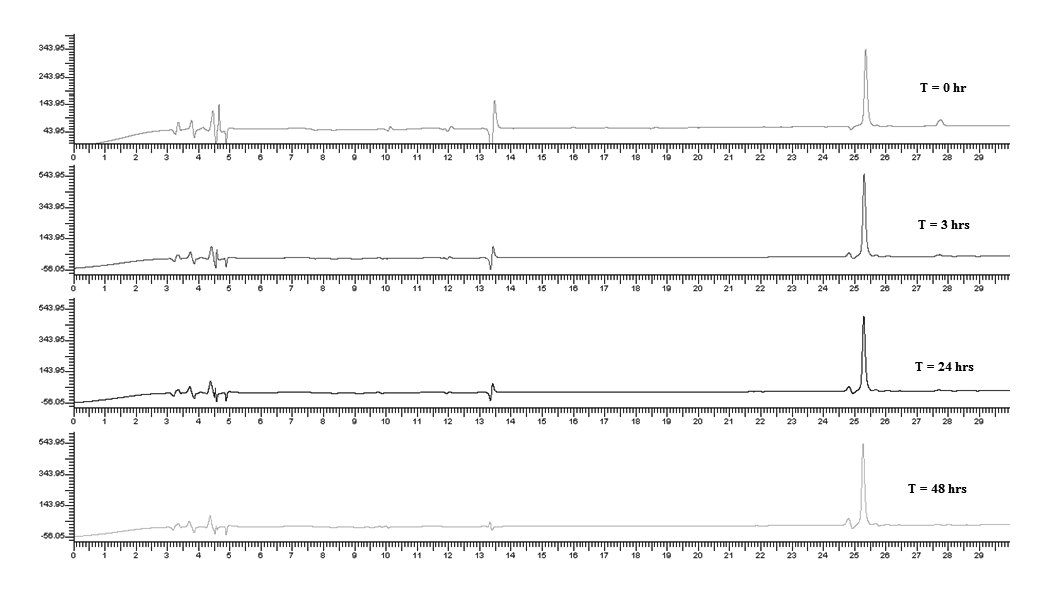
**

Figure S28. RP-HPLC traces for Butyl^4th^-OH peptide (20) at 0/3/24/48 hrs for mouse plasma stability study.

**Butyl^4th^-NH_2_ and Butyl^4th^-OH mixture analysis**

From 400 µg/ml for both peptides, 200 µl of Butyl^4th^-NH_2_ **(16)** and 80 µl of Butyl^4th^-OH **(20)** were mixed and processed as in the plasma stability studies for analysis by RP-HPLC.

**
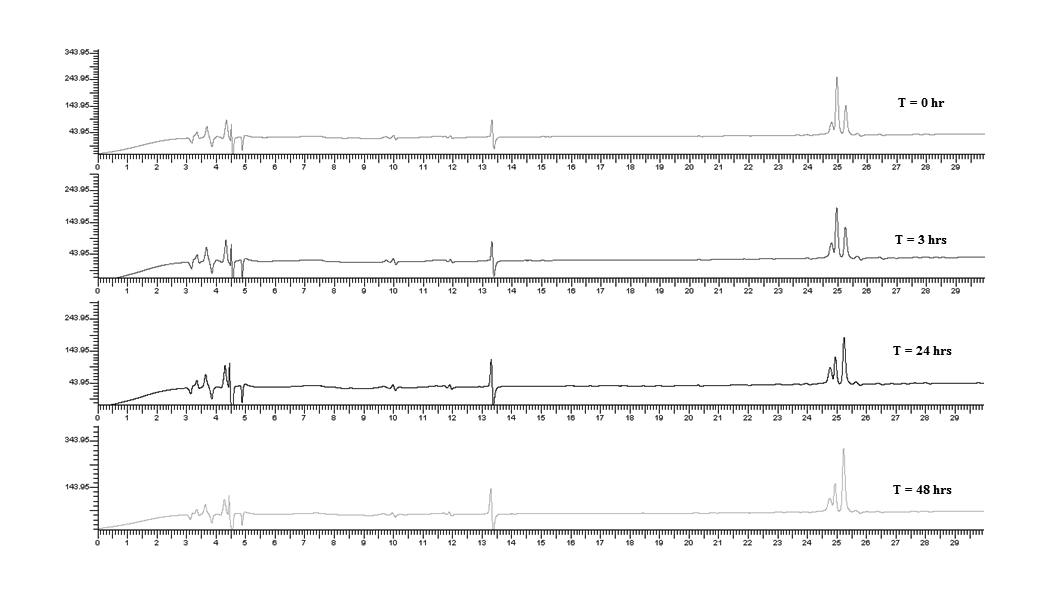
**

Figure S29. RP-HPLC traces for Butyl^4th^-NH_2_ (16) and a lower amount of Butyl^4th^-OH (20) mixture at 0/3/24/48 hrs for mouse plasma stability study.

Butyl^4th^-NH_2_ **(16)** and low Butyl^4th^-OH **(20)** mixture / Mouse plasma stability study


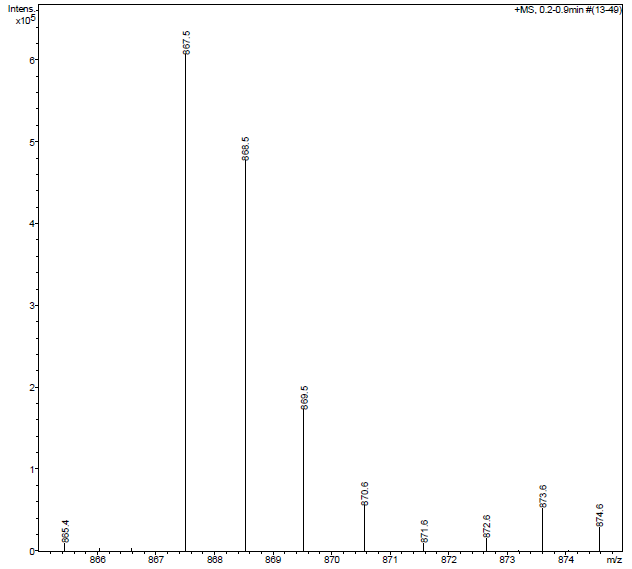

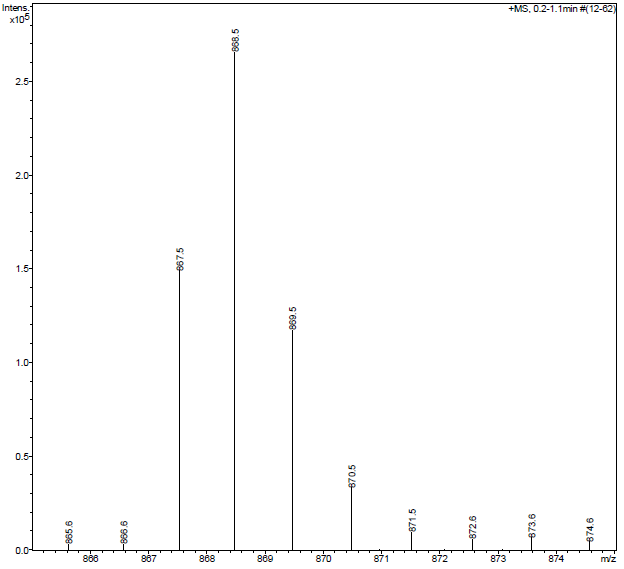


**T = 0 hr**

**Most abundant ion**

***m/z*: 867.5**

**T = 48 hrs**

**Most abundant ion**

***m/z*: 868.5**

Calculated amide (M+H)^+^ = 867.4916 / observed = 867.5 (Most abundant ion at 0 hr)

Calculated carboxylic acid (M+H)^+^ = 868.4756 / observed = 868.5 (Most abundant ion at 48 hrs)

Figure S30. ESIMS for Butyl^4th^-NH_2_ (16) and a lower amount of Butyl^4th^-OH (20) mixture at 0 and 48 hrs in mouse plasma stability study.

*tert*-Prenyl^4th^-NH_2_ **(2)** / Mouse plasma stability study

**
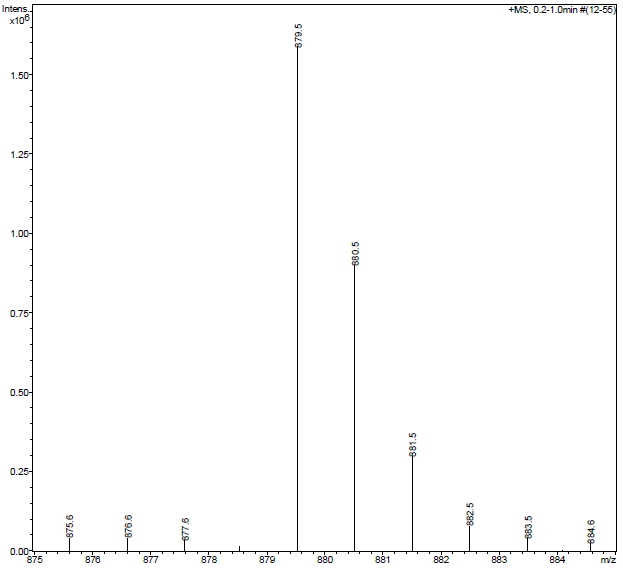

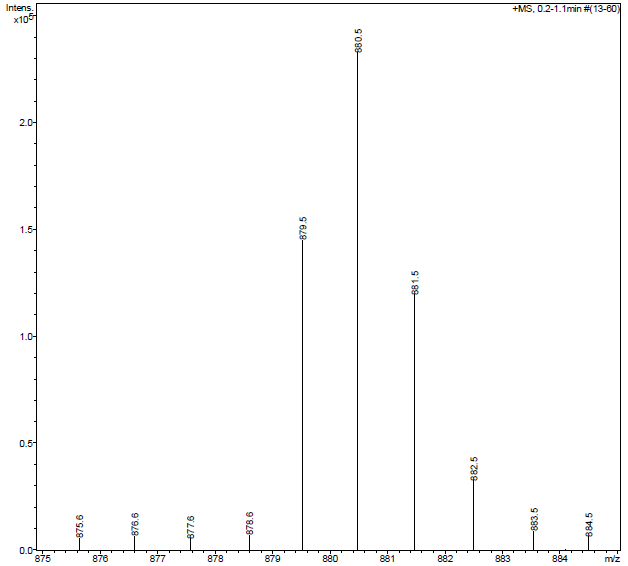
**

**T = 48 hrs**

**Most abundant ion**

***m/z*: 880.5**

**T = 0 hr**

**Most abundant ion**

***m/z*: 879.5**

Calculated amide (M+H)^+^ = 879.4916 / observed = 879.5 (Most abundant ion at 0 hr)

Calculated carboxylic acid (M+H)^+^ = 880.4756 / observed = 880.5 (Most abundant ion at 48 hrs)

Figure S31. ESIMS for *tert*-Prenyl^4th^-NH_2_ (2) at 0 and 48 hrs in mouse plasma stability study.

Butyl^4th^-OH **(20)** / Mouse plasma stability study


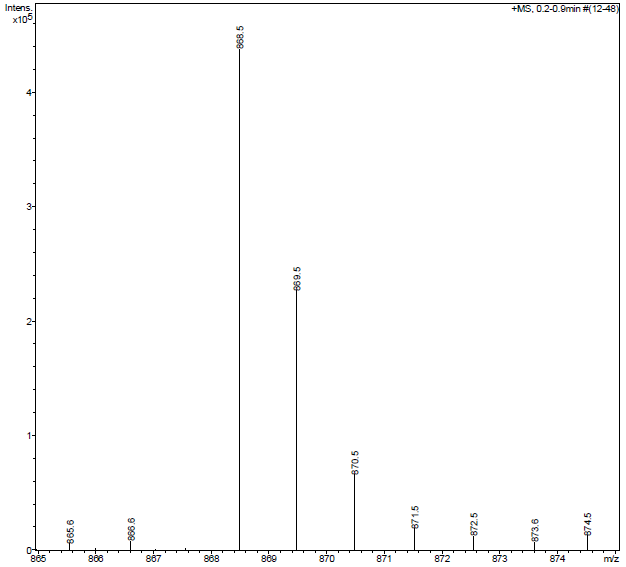

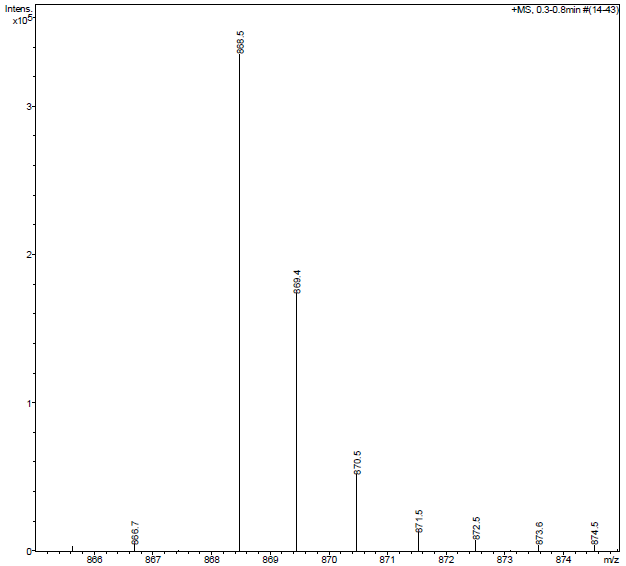


**T = 48 hrs**

**Most abundant ion**

***m/z*: 868.5**

**T = 0 hr**

**Most abundant ion**

***m/z*: 868.5**

Calculated carboxylic acid (M+H)^+^ = 868.4756 / observed = 868.5 (Most abundant ion at 0 and 48 hrs)

Figure S32. ESIMS for Butyl^4th^-OH (20) at 0 and 48 hrs in mouse plasma stability study.

Butyl^4th^-NH_2_ **(16)** / Mouse plasma stability study


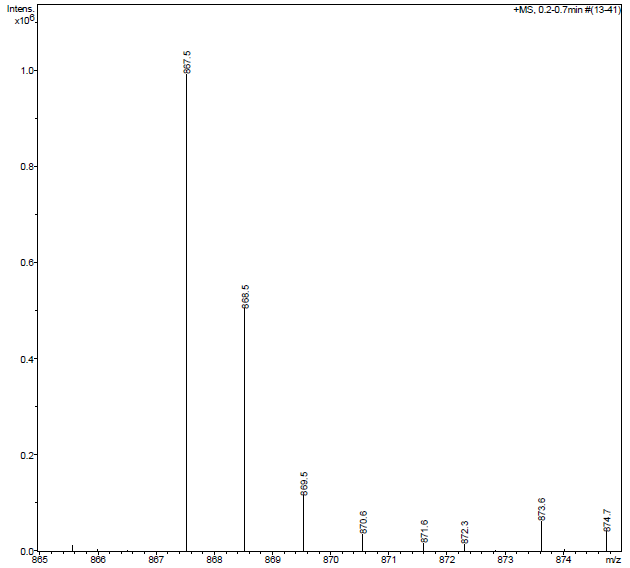

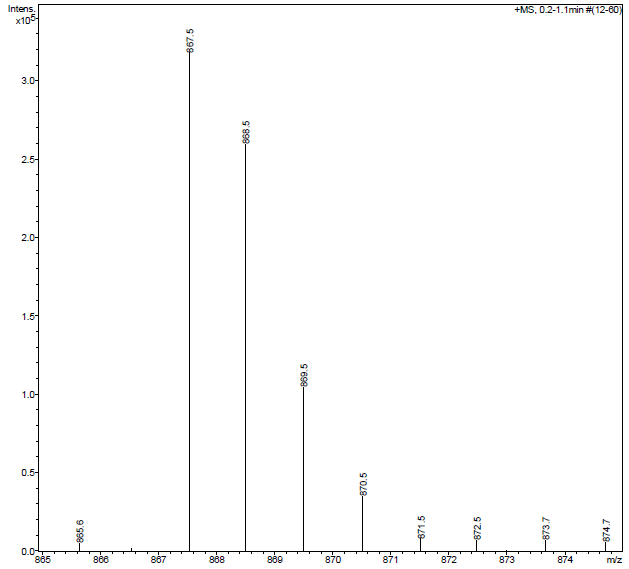


**T = 48 hrs**

**Most abundant ion**

***m/z*: 867.5**

**T = 0 hr**

**Most abundant ion**

***m/z*: 867.5**

Calculated amide (M+H)^+^ = 867.4916 / observed = 867.5 (Most abundant ion at 0 and 48 hrs)

Figure S33. ESIMS for Butyl^4th^-NH_2_ (16) at 0 and 48 hrs in mouse plasma stability study.

Butyl^2nd,4th^-NH_2_ **(19)** / Mouse plasma stability study


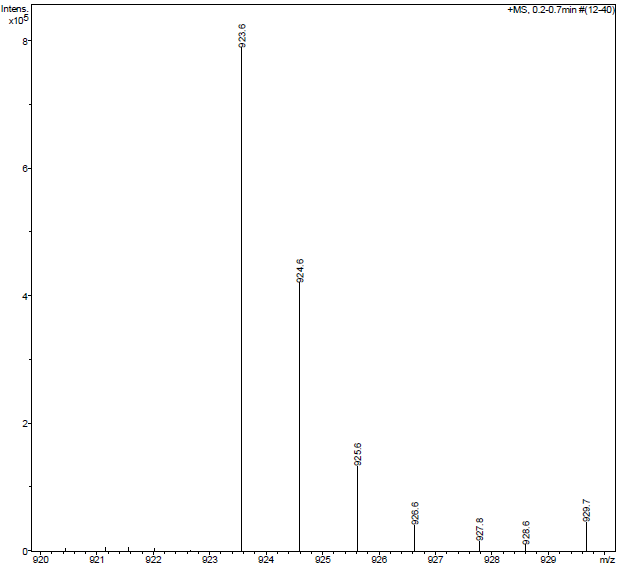

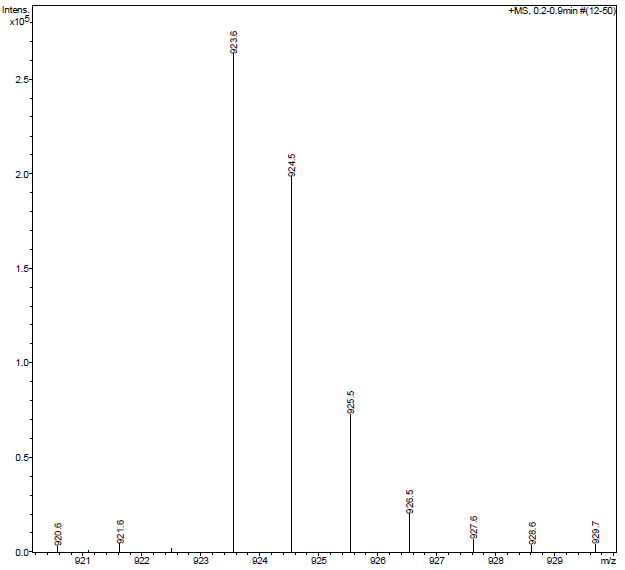


**T = 48 hrs**

**Most abundant ion**

***m/z*: 923.6**

**T = 0 hr**

**Most abundant ion**

***m/z*: 923.6**

Calculated amide (M+H)^+^ = 923.5542 / observed = 923.6 (Most abundant ion at 0 and 48 hrs)

Figure S34. ESIMS for Butyl^2nd,4th^-NH_2_ (19) at 0 and 48 hrs in mouse plasma stability study.

**Butyl^4th^-NH_2_ in S9 liver fraction from mouse**


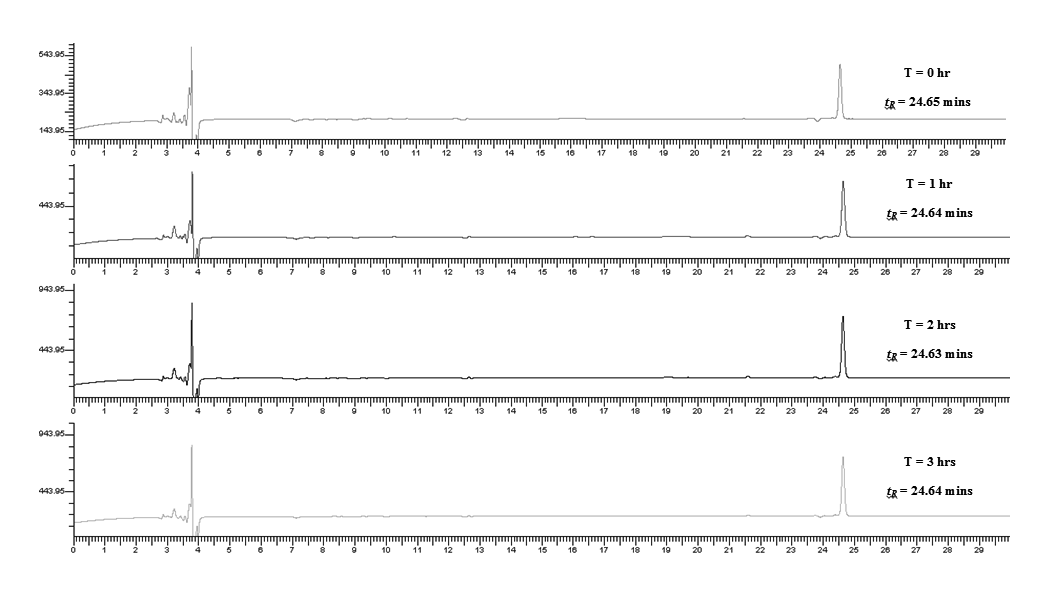


Figure S35. RP-HPLC traces for Butyl^4th^-NH_2_ peptide (16) at 0/1/2/3 hrs for S9 liver fraction from mouse stability study.

**Butyl^2nd,4th^-NH_2_ in S9 liver fraction from mouse**

Figure S36. RP-HPLC traces for Butyl^2nd,4th^-NH_2_ peptide (19) at 0/1/2/3 hrs for S9 liver fraction from mouse stability study.

**B C**

**3.3%**

**± 0.6%**

**23.9%**

**± 2.5%**

**2.3%**

**± 0.2%**

**28.6%**

**± 8.2%**

**6.4%**

**± 0.4%**

**61.7%**

**± 9.1%**

**6.2%**

**± 0.4%**

**67.6%**

**± 3.0%**

**8.0%**

**± 0.6%**

**53.3%**

**± 11.8%**

**36.4%**

**± 11.8%**

**2.3%**

**± 0.2%**

**22.9%**

**± 3.4%**

**2.2%**

**± 0.3%**

**6.6%**

**± 0.1%**

**68.3%**

**± 3.7%**

**95.5%**

**± 0.4%**

**1.6%**

**± 0.2%**

**1.9%**

**± 0.3%**

**1.0%**

**± 0.1%**

**AnnV**

**AnnV**

**AnnV**

**SyB**

**SyB**

**A**

**SyB**

**AnnV**

**AnnV**

**D E**

**SyB**

**SyB**

Figure S37. Dot blots of flow cytometric analysis for apoptosis detection using Annexin V Alexa Fluor® 555 (AnnV) conjugate and SYTOX® Blue (SyB) dead cell stain. DMS79 cells, untreated (A), incubated with 2 µM of 1 (B) and 16 (C) and with 6 µM of 1 (D) and 16 (E) for 24 hours in complete media in 12-well plates and analysed as described in the methods. Average percentage for each quadrant is presented with standard error (SE) (n = 3). On each dot blot, Q1 (top left) represents necrotic cells, AnnV negative (-) and SyB positive (+); Q2 (top right) represents late apoptotic cells, AnnV (+) and SyB (+); Q3 (bottom left) represents live cells, Annv (-) and SyB (-); Q4 (bottom right) represents early apoptotic cells, Ann (+) and SyB (-).
